# Hierarchical Embedded Sphere Model: An Interpretable ML‐Guided Multiscale Descriptor Engineering Decodes OER Activity on TM@MO_2_ Catalysts

**DOI:** 10.1002/advs.202518931

**Published:** 2025-12-19

**Authors:** Ziyuan Li, Shan Gao, Yunhan Wang, Yueyu Zhang, Weichao Wang, Xiangmei Duan

**Affiliations:** ^1^ School of Physical Science and Technology Ningbo University Ningbo China; ^2^ State Key Laboratory of Materials for Advanced Nuclear Energy & School of Materials Science and Engineering Shanghai University Shanghai China; ^3^ College of Electronic Information and Optical Engineering Nankai University Tianjin China

**Keywords:** descriptor engineering, interpretable machine learning, multiscale geometric‐electronic couplings, OER electrocatalys, site‐dependent adsorption behavior

## Abstract

Understanding and predicting catalytic activity in transition‐metal oxides remains challenging due to the multiscale interplay between geometric and electronic structure. Here, we propose a *Hierarchical Embedded Sphere Model* (*HESM*) integrating density functional theory (DFT) with interpretable machine learning (IML) to establish a generalizable descriptor framework for oxide electrocatalysis. By analyzing transition metal‐doped anatase MO2 (101) surfaces, *HESM* disentangles catalytic activity into three hierarchical contributions: Global electronic structure (***G‐*** class), atomic‐site intrinsic properties (***A‐*** class), and local coordination effect (***L‐*** class). The combined effect of these three classes leads to the emergence of two catalytic activation paradigms: Rh@MO_2_ optimizes activity via dopant‐induced electronic modulation (*η*_TD_ = 0.29–0.36 V), while Fe@ZrO_2_ enhances performance (*η*_TD_ = 0.49 V) by host‐site coordination field tuning. The hierarchical framework explains the competitive adsorption of OH^*^ across doped/host sites, reconciles the well‐known V‐shaped activity trends with site‐dependent deviations, and SHapley Additive exPlanation (SHAP) analysis identifies ***G‐*** class as a critical feature that governs the model's prediction. This work demonstrates that *HESM* bridges adsorption energetics with multiscale geometric‐electronic couplings, offering a generalizable methodology for descriptor discovery and catalyst design in complex systems.

## Graphical abstract

*Hierarchical Embedded Sphere Model* combines DFT and interpretable machine learning to decode catalytic activity on TM‐doped MO_2_. It disentangles global electronic, active‐site, and local coordination effects, revealing two activation mechanisms: dopant‐driven (Rh@MO_2_) and coordination‐mediated (Fe@ZrO_2_). This model unifies OH^*^ competition and V‐shaped adsorption trends. SHAP analysis identifies ***G‐*** class as the dominant descriptor for oxide electrocatalysis.

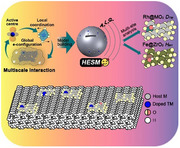

## 1 Introduction

The OER under acidic conditions remains a critical bottleneck for proton‐exchange membrane water electrolyzers, a key technology for sustainable hydrogen production [1, 2, 3, 4]. This limitation stems primarily from the sluggish kinetics of the four‐electron transfer process [5, 6]. While noble metal oxides like RuO_2_ and IrO_2_ exhibit promising intrinsic activity due to favorable electronic structures [7, 8], their large‐scale development is constrained by prohibitive cost (e.g., IrO_2_ at ∼$4600/oz) and scarcity [9, 10, 11]. To overcome these challenges, strategies such as alloying [12, 13, 14, 15, 16], development of alternative oxides (e.g., perovskites, spinels, and pyrochlores) [17, 18, 19, 20]. have been pursued. Additionally, selective heteroatom doping significantly enhances performance through tailored electronic structural modifications [21, 22]. For instance, Co doping in SrIrO_3_ perovskite preserves IrO_6_ octahedra while boosting OER activity [23], and Ru substitution in Co_3_O_4_ spinel enhances both acidic OER activity and stability [24]. Despite these advances in the enhancements of OER performance, a fundamental challenge remains: establishing universal descriptors to rationally select dopants for the target oxide catalyst.

Resolving these challenges requires clarifying the origins and regulation mechanisms of catalytic activity — a more formidable task for oxides than for metals. Conventional models like *d*‐band center theory rationalize transition metal activity through *d*‐orbital energy positions, where elevated *d*‐states strengthen adsorbate binding via enhanced electron donation/back‐donation [25, 26, 27]. For complex systems such as high‐entropy alloy catalysts, performance proves highly sensitive to local coordination, better captured by *d*‐band filling indices accounting for bandwidth effects [28]. In contrast, oxide catalysts feature metal‐oxygen coordination that breaks *d*‐orbital degeneracy through symmetry reduction, inducing energy‐level splitting and yielding intricate electronic structures. This complexity is amplified by diverse exposed surfaces and doping modifications, which trigger elusive structural rearrangements and electronic redistributions, obscuring structure‐activity relationships [29, 30, 31]. Therefore, individual descriptors, including O *p*‐band center [32, 33, 34, 35], *e_g_* orbital occupancy [36], metal‐oxygen coordination field [37], and metal oxidation states [38], remain limited‐specific and fail to universally describe doping‐induced geometric‐electronic couplings. Developing a generalizable descriptor framework thus requires machine learning approaches that can navigate complex electronic, atomic, and structural feature spaces to enable rational catalyst design [39].

In this study, we developed a Hierarchical Embedded Sphere Model (HESM) based on a hierarchical analysis strategy to clarify the multiscale origins of catalytic activity. The model categorizes descriptors into three physically interpretable classes: G‐class, which captures the global electronic characteristics of the oxide; A‐class, which represents the intrinsic properties of atomic sites; and L‐class, which describes the local coordination distortions associated with site centers. Using doped anatase MO2 (101) surfaces as a representative system, we combine density functional theory (DFT) with interpretable machine learning (IML) to evaluate nonequivalent configurations (R^2^ = 0.96). It is noted that the ***G‐***, ***A‐***, and ***L‐*** class features account for the total importance by 52.4%, 32.4%, and 15.2%, respectively, quantitively demonstrates the combined effect of global and local catalytic properties. SHapley Additive exPlanation (SHAP) analysis evidences two types of high‐performance catalysts: Rh@MO_2_ achieves optimal activity (*η*_TD_ = 0.29–0.36 V), while Fe@ZrO_2_ enhances host‐site performance (*η*_TD_ = 0.49 V). Critically, the coupling of site‐dependent OH^*^ adsorption (V‐shaped) with multiscale geometric‐electronic structures reveals the synergetic contribution of dopant/host to the catalytic performance, elucidating the OER activity trend on doped oxide surfaces. By establishing a theoretical framework based on hierarchical descriptors, *HESM* bridges catalytic activity with atomic‐scale regulations, offering a physically interpretable and generalizable toolkit for refining complex oxide surfaces and further designing high‐performance electrocatalysts.

## 2 Results and Discussion

### 2.1 Hierarchical Analysis of Catalytic Activity

**FIGURE 1:**
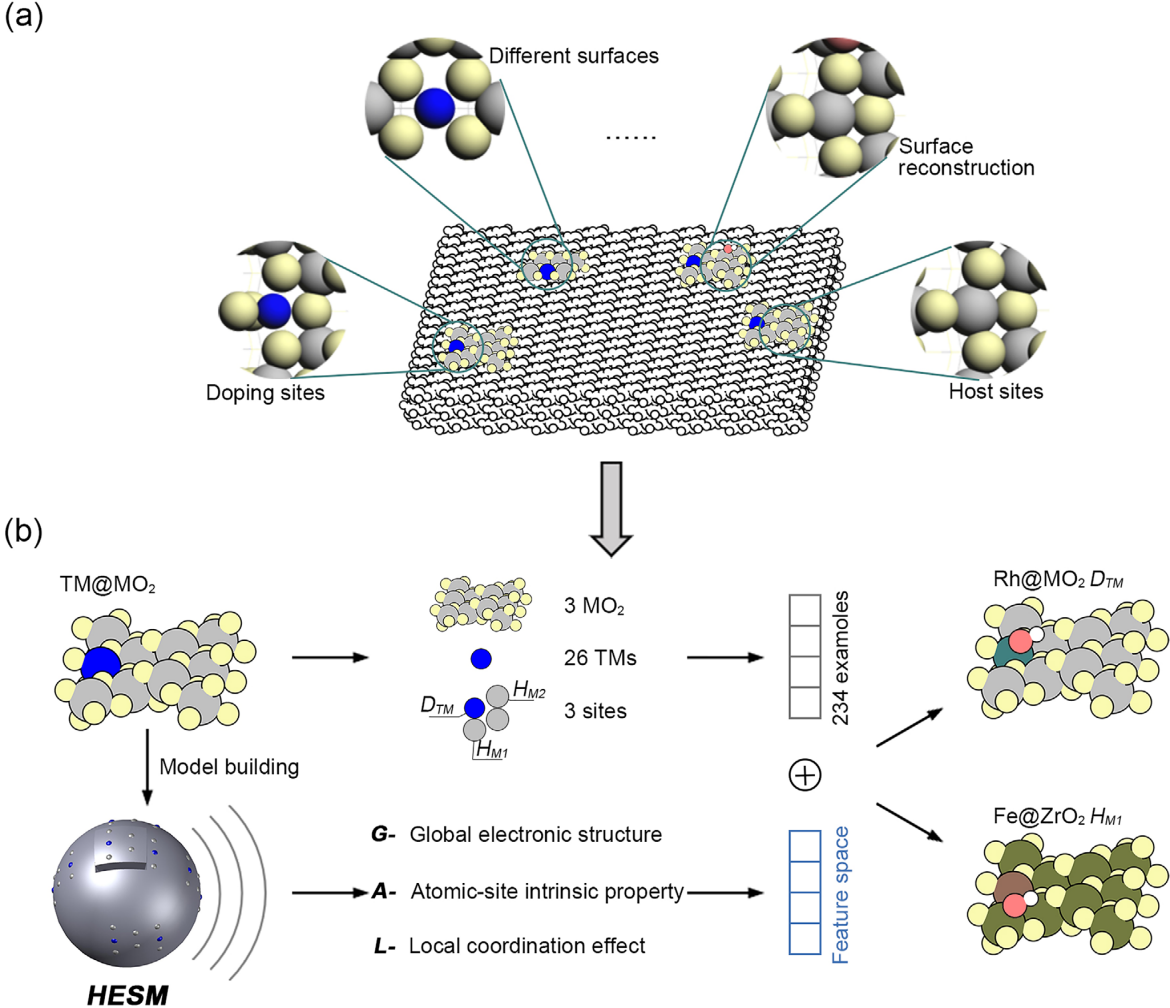
The schematic diagram of the hierarchical analysis of catalytic activity. (a) Complex surfaces of metal oxides; (b) For the constructed complex surfaces of metal oxides, high‐performance reaction sites are identified through *HESM*, and their active origins are decoded by hierarchical analysis.

Metal oxide surfaces — particularly when doped with transition metal (TM) — exhibit complex structural features, including distinct exposed facets, dynamic surface reconstruction, and different dopant and host site configurations (Figure 1a). These factors complicate structure‐activity relationships, necessitating a fundamental understanding of the underlying geometric‐electronic interactions.

Here, we proposed a hierarchical analysis strategy (Figure 1b) to interpret OER catalytic origins. This approach systematically addresses the complexity of single‐atom‐doped metal oxide surfaces across three levels: the MO_2_ matrix, doped TM atoms, and adsorption sites (*D_TM_* refers to TM dopants, *H_M1_* and *H_M2_* refer to two types of host sites). We generated and analyzed 234 nonequivalent surface configurations on the anatase MO_2_ (101) using single‐site doping. A *Hierarchical Embedded Sphere Model* was developed to quantify TM@MO_2_ catalytic activities through three feature categories: (i) ***G‐*** class (global electronic features of doped structures), (ii) ***A‐*** class (atomic properties of active centers), and (iii) ***L‐*** class (local coordination‐field distortions). By applying IML to correlate OER performance with feature space, we identified complex active sites.

### 2.2 Construction of *HESM* for TM@MO_2_

The traditional single‐site catalytic model generally includes three types of features: (I) intrinsic atomic features (labeled as AF), such as the valence electron number (N_d_), van der Waals radius (R_v_), etc.; (II) electronic structural properties obtained from DFT calculations, including intermediate charge transfer quantities (CT*_e_*), the *d*‐band center (*ε_d_*), etc.; and (III) simple descriptors derived from combinations of intrinsic properties, such as composite metrics for charge transfer capacity [40, 41]. While these features offer a fundamental framework for understanding catalytic activity in simple systems, they fall short in describing more complex catalytic environments. In the MO_2_ system, TM dopant induces irregular distortions in the surface coordination environment, necessitating the development of a generalizable catalytic model to address these complexities, as detailed in the following discussion.

#### 2.2.1 Dissection (*h‐*, *d‐*, *c‐*, *s‐* Type Features)

**FIGURE 2:**
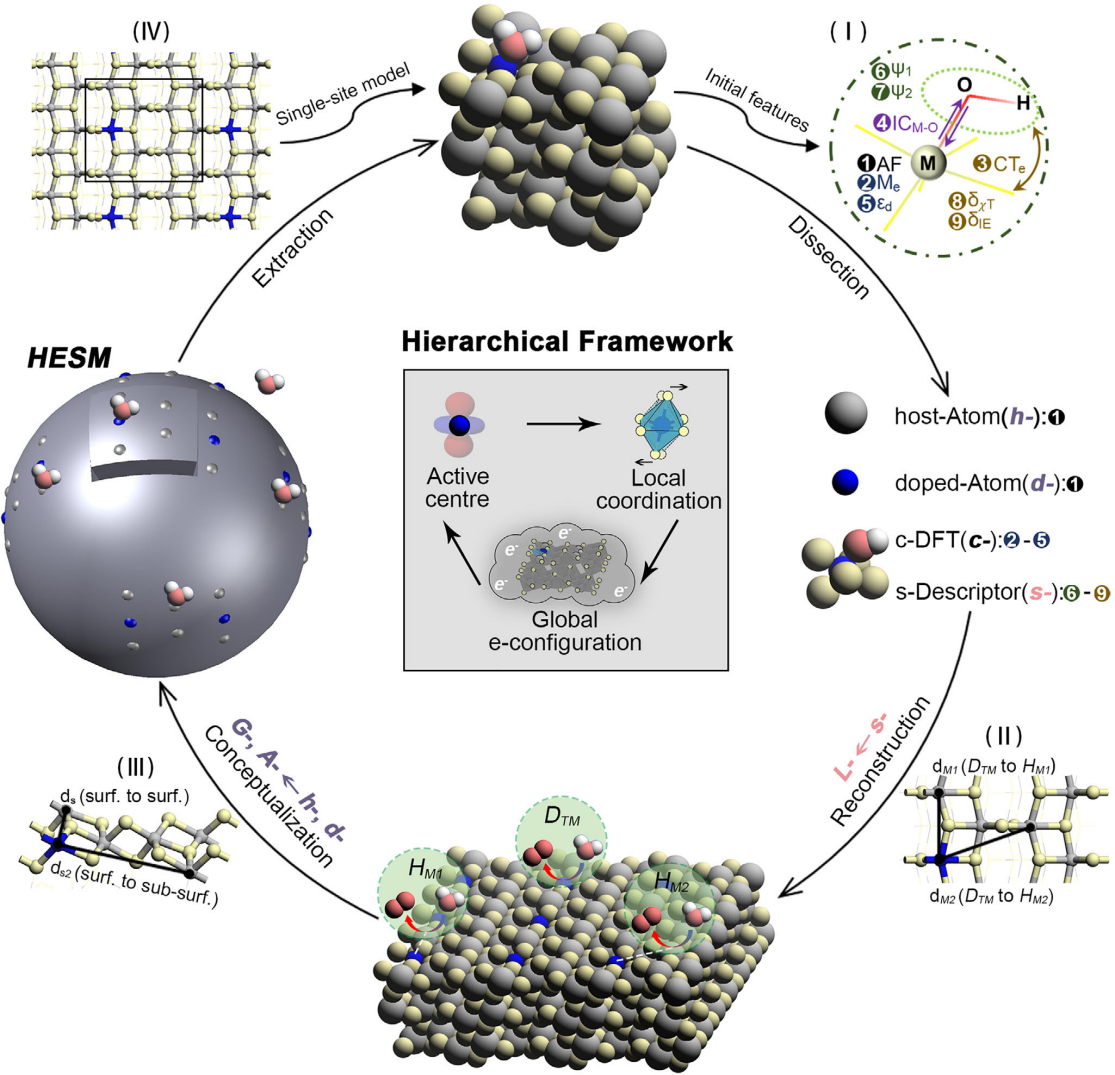
Conceptual diagram of ML feature engineering for *HESM*. Schematic illustration of multistage feature evolution in the catalytic model, highlighting the features contained in each model stage and the corresponding changes. The central part presents a hierarchical framework that influences the catalytic performance of doped surfaces, including three key factors: intrinsic properties of the active center, local coordination field distortion, and global electron redistribution. The four insets along the pathway respectively represent: (I) Nine initial features; (II) The parameters considered the geometric structure weights *w_L_* (d*_M1_* and d*_M2_*); (III) Further refinement of the model incorporates parameters into the geometric structure weight *w_G_* (d_s_ and d_s2_); (IV) A single‐site model extracted from the periodic structure. Gray and Blue spheres represent the host and doped atoms, respectively. Yellow and red spheres represent oxygen atoms within the MO_2_ lattice and oxygen intermediates, respectively.

Figure 2 illustrates our hierarchical framework, which builds upon the single‐site catalytic model (Text S1) by decomposing the surface into three components: host metal atoms (host‐Atom), dopant TM atoms (doped‐Atom), and the reaction zone. Host‐ and doped‐Atom are described by ***h‐*** type and ***d‐*** type atomic features, respectively. Interactions within the reaction zone are characterized using DFT‐calculated features (***c‐*** type) and simple descriptors (***s‐*** type) [Figure 2, inset (I)].

#### 2.2.2 Reconstruction (*L‐*Class Features)

To account for the differential influence of *D_TM_* on *H_M1_* and *H_M2_* sites due to their spatial positions, we introduced ***L‐*** class feature descriptors incorporating a geometric weighting factor *w_L_* and an adaptive factor *w_1_*. The factor *w_L_* is derived from the geometric distance d_M_ (M = *M1*, *M2*) between *H_M1_*/*H_M2_* and *D_TM_* [Figure 2, inset (II)], reflecting structure‐dependent effects. The factor *w_1_*, initially set to 0.05, is used to quantify the physical response of the adsorption sites perturbed by dopants to the reactants. (details in Text S1). These descriptors quantitatively capture the dopant‐induced local coordination modifications.

#### 2.2.3 Conceptualization (*G‐*, *A‐* Class Features)

To address the varying OER probabilities at *D_TM_*, *H_M1_*, or *H_M2_* sites, ***h‐*** and ***d‐*** type features were optimized to ***G‐*** class using a geometric weighting factor *w_G_* (based on distances d_s_ and d_s2_ between dopant and surface/sub‐surface host atoms) and an adaptive factor *w_2_* (initially 0.5) [Figure 2, inset (III)]. ***G‐*** class features incorporate host/doped atom counts on surface and sub‐surface layers to uniformly describe global electronic properties (represented as a large sphere). Site‐specific intrinsic properties are captured by ***A‐*** class features (original ***h‐*/*d‐*** types), forming an embedded small sphere. This *Hierarchical Embedded Sphere Model* integrates global and local effects. Details can be found in Text S1.

#### 2.2.4 Extraction

The *HESM* unifies simple and complex catalytic systems through hierarchical geometric coordination and feature optimization. By extracting active regions and reducing feature dimensions — particularly by eliminating geometric weights — it maintains predictive accuracy for single‐site catalysts [Figure 2, inset (IV)]. This approach bridges catalytic complexity gaps while ensuring consistent interpretability.

#### 2.2.5 *HESM* Defines Three Catalytic Scales

① ***G‐*** class (system‐level), which encompasses the global structural attributes of TM@MO_2_ systems, dictating the overall electronic‐geometric environment; ② ***A‐*** class (atomic‐level), representing the intrinsic chemical traits of surface metal atoms; ③ ***L‐*** class (site‐level), addressing localized site variations, providing atomic‐level corrections to active site performance. The hierarchical framework is inherently adaptable to different materials, surfaces, and catalytic reactions. The material‐specific geometric weighting factors (*w_L_*, *w_G_*) and adaptive factors (*w_1_*, *w_2_*) enable the model to capture both global and local reactivity on complex surfaces. Descriptors related to reaction centers (e.g., ***L‐*** ψ_2_, ***L‐*** δ_XT_) already integrate electronic structural information of key intermediates. Consequently, when applying the framework to different systems, only the relevant features need to be substituted.

### 2.3 Site‐dependent Catalytic Behaviors in OER

Guided by the *HESM* framework, we systematically investigated the OER catalytic performance of anatase‐phase MO_2_ (M = Ti, Fe, Zr, Nb, Ir, Sn) [42]. Surface Pourbaix diagram analysis identified TiO_2_, ZrO_2_, and SnO_2_ as stable under reaction potential and pH (Text S2). We constructed 1 × 2 × 1 supercells of the catalytically active (101) facet of MO_2_, which features unsaturated metal‐oxygen octahedral coordination, and enhanced OER activity by doping with 26 single‐atom TMs (Figure S8). All doped systems showed negative binding energies (*E_b_*) and satisfied the stability criterion *E_b_*/*E_c_* > 0.5, where *E_c_* is the cohesive energy of isolated TM (Figure S21) [43]. A*b initio* molecular dynamics (AIMD) simulations on selected systems — TM@TiO_2_ (TM = Mn, Rh, Pd, Ag), TM@ZrO_2_ (TM = Co, Zn, Ru, Rh), and TM@SnO_2_ (TM = Ti, Zr, Rh, Hf) — confirmed thermodynamic stability, with negligible energy/temperature fluctuations and no significant structural distortion (Figures S22–S24).

Competitive OH^*^ adsorption on *H_M1_*/*H_M2_* and *D_TM_* sites was systematically evaluated. Using minimum adsorption energy as the thermodynamic criterion [$Min(\Delta E_{OH*}^{S},S=D_{TM},H_{M1},H_{M2})$], predominant OH^*^ sites were identified (Figure S25). OH^*^ stabilizes preferentially on atoms with higher energy *d*‐states [44], favoring thermodynamically favorable configurations. When the dopant period is lower than the host substrate's, OH^*^ tends to adsorb at *H_M1_*/*H_M2_* sites due to their more delocalized *d*‐states near the Fermi level. The reverse holds for higher‐period dopants. For systems with matched *d*‐states, mid‐period dopants show stronger OH affinity, though trends remain unclear. Competition between *H_M1_* and *H_M2_* sites remains poorly understood due to local symmetry‐breaking effects.

**FIGURE 3:**
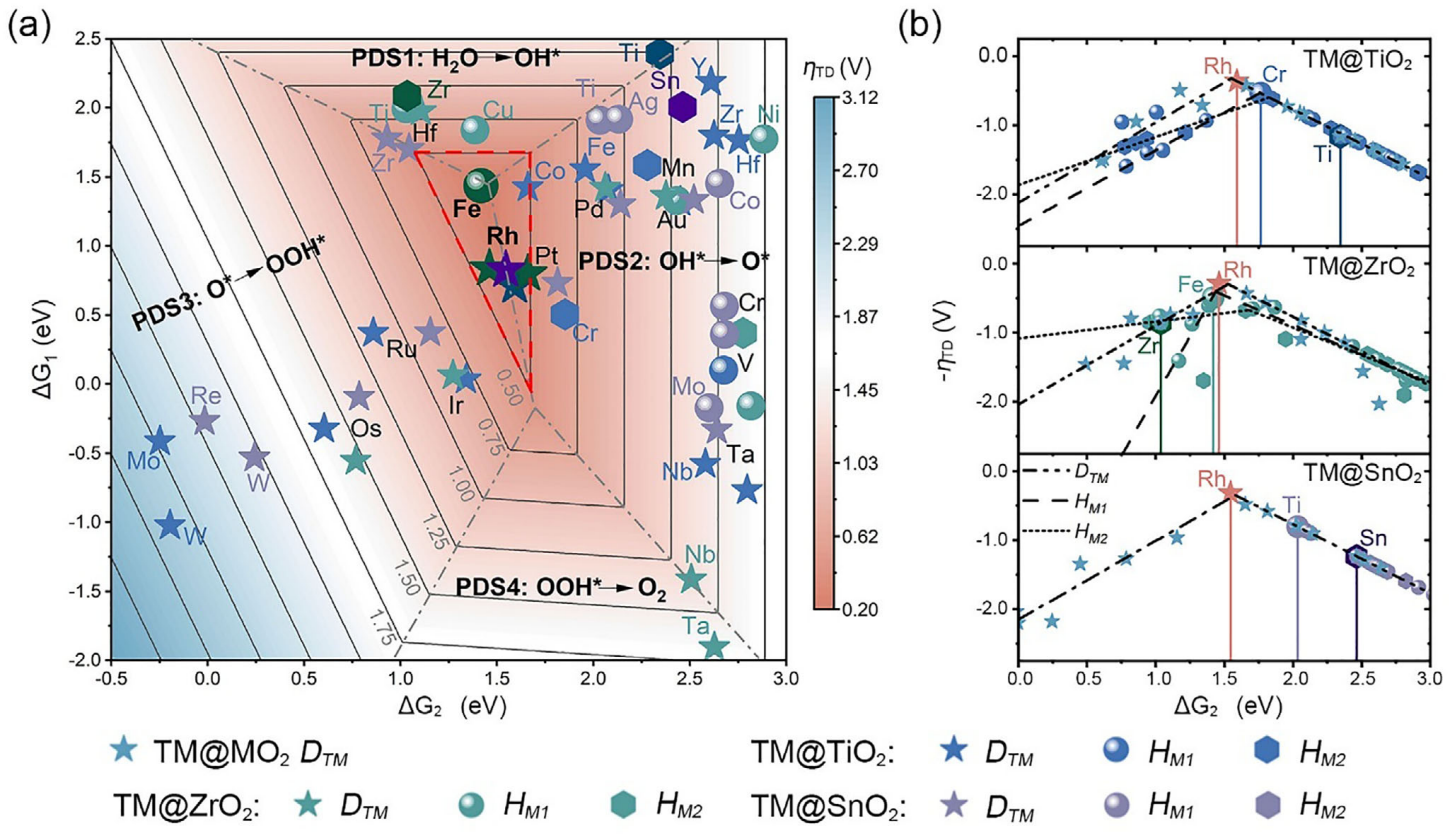
OER catalytic activity analysis in TM@MO_2_. (a) 2D theoretical overpotential (*η*_TD_) volcano plot based on ΔG_1_ and ΔG_2_. Gray dashed lines divide regions dominated by distinct PDSs, while red dashed lines mark the optimal catalytic activity zone. (b) Site‐dependent OER activity distribution: Volcano plot for TM@MO_2_ surfaces with heterogeneous active sites.

The 2D OER activity volcano (Figure 3a) integrates two fundamental relationships: (i) the ΔG_2_ (G_O*_–G_OH*_)‐*η*_TD_ volcano (Figure S26) [45, 46, 47, 48], with peak activity at ΔG_2_ ≈ 1.5 eV—consistent with optimal OH^*^ deprotonation energy (1.5–1.7 eV) [49, 50]—and (ii) ΔG_2_‐ΔG_1_ (G_OH*_–G_*_)‐ΔG_3_ (G_OOH*_–G_O*_) scaling trends (Figures S27–S29), resulting from *d*‐band center shifts under MO_2_ lattice constraints. This framework reveals two high‐performance regimes: *D_TM_*‐dominated catalysts (Rh@MO_2_, *η*_TD_ = 0.29–0.36 V; pathway in Figure S30) and host‐activated systems (Fe@ZrO_2_, *η*_TD_ = 0.49 V), both achieving near‐ideal energetics via distinct active sites.

The site‐dependent volcano plots (Figure 3b) show distinct catalytic behaviors across TM@MO_2_ systems. TM@TiO_2_ and TM@ZrO_2_ exhibit a triple volcano relationship with optimal ΔG_2_ values varying among *D_TM_*, *H_M1_*, and *H_M2_* sites, while TM@SnO_2_ displays single volcano behavior due to Sn^4+^ redox inertia. Host material properties influence OER activity at *Host‐S* sites: high ΔG_2_ of pristine TiO_2_ (2.35 eV) shifts its *Host‐S* peak rightward, reducing Cr@TiO_2_ activity (ΔG_2_ = 1.76 eV). Low ΔG_2_ of ZrO_2_ (1.04 eV) shifts its peak leftward, yielding optimal Fe@ZrO_2_ (ΔG_2_ = 1.42 eV). SnO_2_‘s higher ΔG_2_ (2.46 eV) confines its plot to the right, resulting in suboptimal Ti@SnO_2_ (ΔG_2_ = 2.03 eV). *Host‐S* sites can outperform *D_TM_* via coordination regulation, challenging the assumption of *D_TM_*‐dominated catalytic activity.

### 2.4 Discrimination of Contribution for Hierarchical Descriptors within IML

Given scaling relations constraints where ΔG_1_ systematically influences adsorption energies, we analyzed correlations between ΔG_1_ and electronic descriptors. Across TM@MO_2_ systems, ΔG_1_ and CT*_e_* show a strong correlation at *D_TM_* sites, while *H_M1_* and *H_M2_* deviate. Similar trend holds for ΔG_1_ vs IC_M‐O_ (Integral crystal orbital Hamilton population of the M‐O bond), IC_M‐O_ vs CT*_e_*, and IC_M‐O_ vs M*_e_* (metallic atom electron transfer) (Text S3; Figures S31–S33). These relationships break down at *H_M1_*/*H_M2_* sites. Single descriptors prove insufficient for capturing TM@MO_2_ surface complexity, motivating IML to resolve activity origins [51, 52, 53].

**FIGURE 4:**
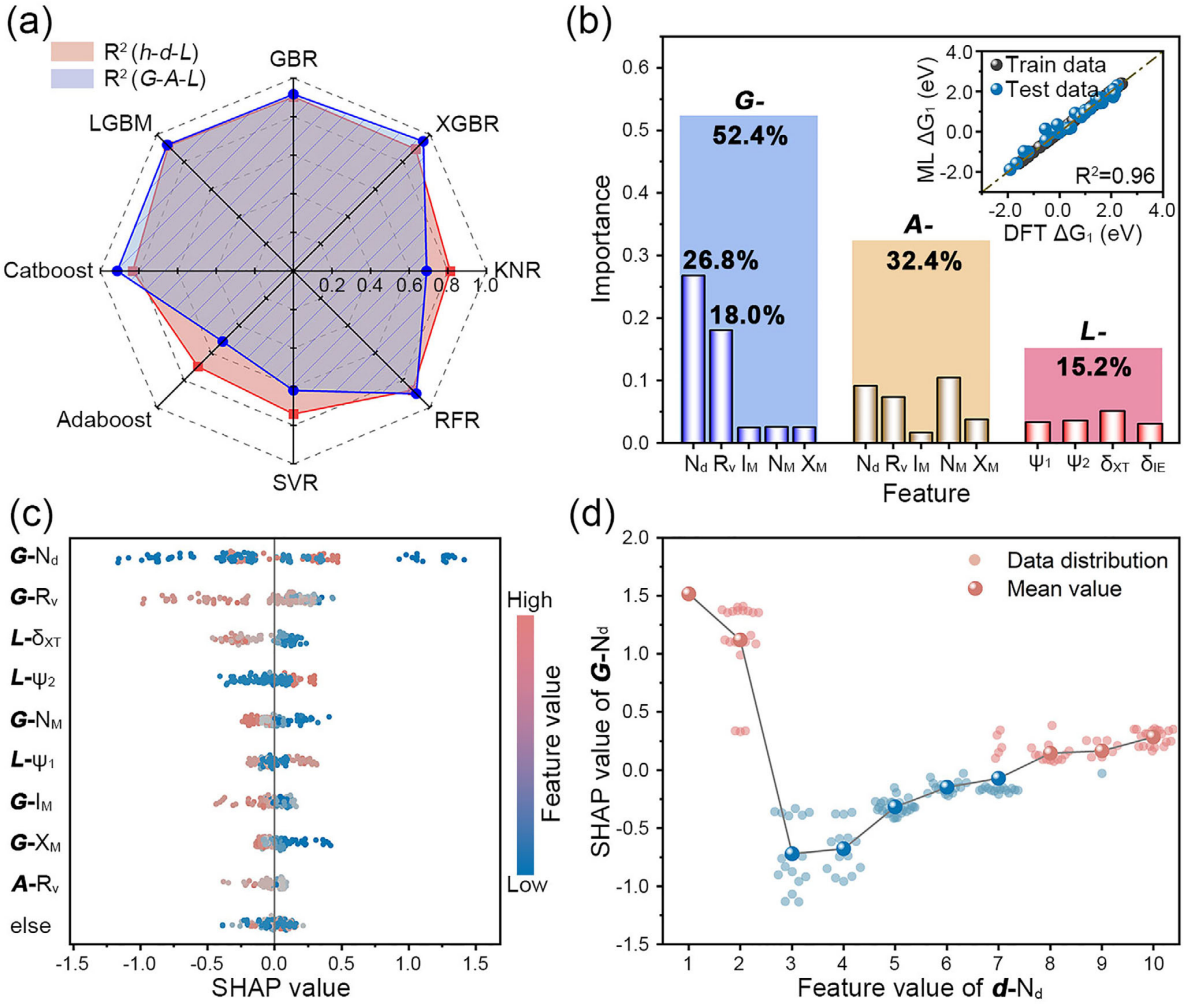
Construction and analysis of IML frameworks. (a) Radar chart comparing R^2^ across 8 regression algorithms for two feature combinations; (b) Stacked weight distribution across three descriptor categories in the XGBR model, the inset shows the consistency of DFT‐ML; (c) Summary plot ranking descriptors by mean SHAP values. Data point color mapping corresponds to the normalized feature values, with horizontal distribution representing the SHAP contribution direction; (d) Distribution of ***G‐*** N_d_ in the ***d‐*** N_d_ projection space (blue indicates negative influence, red indicates positive influence).

Based on *HESM* (***G***, ***A***, ***L‐*** class and ***c‐*** type), with ΔG_1_ as the target variable, we constructed feature intercorrelation matrices (Figure S9), revealing strong Pearson correlations between ***c‐*** type and ***L‐*** class features—justifying the exclusion of ***c‐*** type features in final models. Using an 8:2 train‐test split, we evaluated eight ML algorithms (GBR, XGBR, LGBM, KNR, RFR, SVR, Catboost, and Adaboost) via R^2^ scores (Figure 4a). Comparing feature sets (***h***, ***d***, ***L‐*** types vs ***G***, ***A***, ***L‐*** class), LGBM (R^2^ = 0.92) and GBR (R^2^ = 0.90) showed high accuracy on the former, while XGBR (R^2^ = 0.95) and LGBM (R^2^ = 0.92) performed the best on the latter, demonstrating the robustness of tree‐based boosting under feature correlations. The data distribution within the training set (Figure S10) was well‐balanced across various feature categories. This not only effectively averted information leakage caused by feature similarity but also guaranteed the reliability of our results.

To enhance the physical interpretability of the ML model, parameters *w_1_* and *w_2_* were tuned within ranges of 0.05–0.25 and 0.3–0.7, using XGBR. The highest R^2^ (0.96) was achieved at *w_1_* = 0.25 and *w_2_* = 0.7 (Figure S11). Corresponding ML predictions vs DFT values are shown in Figure 4b. Feature contributions were: ***G‐*** class (52.4%), ***A‐*** class (32.4%), and ***L‐*** class (15.2%). Individually, ***G‐*** N_d_ and ***G‐*** R_v_ were most important (26.8% and 18.0%, respectively). Additionally, to demonstrate the stability and transferability of the *HESM*, additional details regarding the model's training and validation procedures have been provided in Text S4. A sensitivity analysis was conducted by adjusting *w_1_* and *w_2_* by ±20% (Figures S12–S15), and the results indicate that the model's predictive accuracy, the hierarchical contributions of each feature category, and its physical interpretability all remain stable when subjected to such moderate parameter perturbations.

Furthermore, leave‐one‐out validation (with MO_2_ as ZrO_2_ and TM as Rh; Figure S16) shows that the model can achieve high predictive accuracy (R^2^ = 0.89) even for systems absent from the training set, which confirms its robustness. Given that ΔG_2_ serves as a key descriptor for OER performance, we also applied the same hierarchical structure to predict the adsorption free energy of O^*^ intermediate (Figure S17). With restricted physical adjustments, the model achieved a predictive performance of R^2^ = 0.89, which further demonstrates the strong extrapolation capability of *HESM*. The relevant feature data had been organized in Tables S4–S11.

SHAP analysis provided global and local insights to ensure model interpretability [54, 55]. The SHAP heatmap illustrates each feature's marginal contribution to activity. Catalysts with total SHAP values (the sum of all features) near the base value (0.93) are likely highly active (Figure S34). Global feature interpretation via average SHAP values confirmed feature significance hierarchy (Figure S35), with ***G‐*** N_d_ (≈0.45) being most critical, consistent with XGBR results.

As a guide for OER catalyst design, Figure 4c shows a SHAP summary plot analyzing global feature contributions. While dominant ***G‐*** N_d_ features lack direct correlation with SHAP values, their periodic trend emerges in dependency analysis (Figure S36). Considering the strong correlation between ***G‐*** class and ***d‐*** type features, we projected ***G‐*** N_d_ onto ***d‐*** N_d_ (Figure 4d). Based on this local feature interpretation, ***d‐*** N_d_ = 1, 2 yields excessive OH^*^ adsorption energy, which is unfavorable for the reaction. Within the range ***d‐*** N_d_ = 3 to 10, ***G‐*** N_d_ exhibits a positive correlation with its SHAP value, aligning with V‐shaped adsorption behavior reported by Kirsten T. Winther et al. [56]. Furthermore, they explain the outliers observed in the scaling relationships discussed earlier: elements such as Hf and Zr (***d‐*** N_d_ = 2) exhibit weak OH adsorption, resulting in lower Gibbs free energy changes for O^*^, whereas Ta and Nb (***d‐*** N_d_ = 3, 4) have overly strong OH adsorption, making the deprotonation step exceptionally challenging (Figures S27–S29). These findings are consistent with the *HESM*’s emphasis on ***G‐*** class characteristics, thereby validating its conceptual framework and utility in describing the catalytic system.

### 2.5 Mechanistic Insights of Synergistic Interaction for Hierarchical Descriptor

**FIGURE 5:**
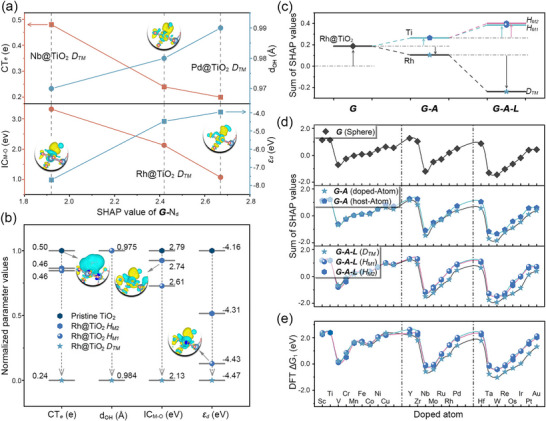
Multiscale mechanistic insights into catalytic surface interactions: electronic structure, adsorption dynamics, and feature interpretability. Multiparameter correlation analysis of CT*_e_*, d_OH_, IC_M‐O_, and *ε_d_* at (a) the *D_TM_* sites on Nb/Rh/Pd‐ TiO_2_ surfaces and (b) the different active sites (*H_M2_*/*H_M1_*/*D_TM_*) on the surfaces of Rh@TiO_2_ and pristine TiO_2_. The inset shows the 3D isosurface plots of charge density difference (±0.002 *e*·bohr^−3^, with yellow/cyan representing electron density accumulation and depletion, respectively); (c) Take Rh@TiO_2_ as an example to explain the SHAP value sum and its variation at different OH adsorption stages; (d) SHAP‐based *HESM* analysis of multilevel feature contributions; (e) DFT‐validated hierarchical energy splitting revealing the discretized distribution of site‐dependent ΔG_1_ on the doped surface.

IML SHAP analysis identified ***G‐*** N_d_ as the most important feature. Using TM@TiO_2_ as a case study, we investigated its role in governing surface electronic properties. SHAP force plots revealed that *D_TM_* sites exhibit catalytic contributions correlated with ***G‐*** N_d_: Nb‐doped TiO_2_ (***d‐*** N_d_ = 4, ***G‐*** N_d_ = 1.92) suppressed OER activity, while Rh‐doped (***d‐*** N_d_ = 8, ***G‐*** N_d_ = 2.42), and Pd‐doped (***d‐*** N_d_ = 10, ***G‐*** N_d_ = 2.67) systems enhanced it (Figure S37). Further electronic structure analysis via projected density of states (PDOS) and COHP for adsorbed OH^*^ species showed four key trends with increasing ***G‐*** N_d_ (3 ≤ ***d‐*** N_d_ ≤ 10) (Figure 5a): reduced OH^*^charge transfer (0.48→0.24→0.20 *e*), elongated OH^*^ bond length (0.97→0.98→0.99 Å), decreased IC_M‐O_ values (3.32→2.13→1.07 eV), and upshifted *d*‐band center (−7.69→−4.47→−3.96 eV). These results indicate weakened OH^*^ binding, quantitatively consistent with activity trends.

Beyond *D_TM_* sites, we examined catalytic variability across host sites. In optimal Rh@TiO_2_, SHAP force plots of OH^*^ adsorption at *H_M1_*, *H_M2_*, and pristine Ti sites revealed strong electronic modulation (Figure 5b; Figure S37). SHAP contributions decreased from pristine Ti (2.40) to *H_M2_* (1.44) and *H_M1_* (1.42), with reduced charge transfer (0.50→0.46→0.46 *e*) and consistent OH bond length (∼ 0.98 Å). Bonding strengths weakened (2.79→2.74→2.61 eV), and *ε_d_* downshifted (−4.16→−4.31→−4.43 eV), converging toward *D_TM_* values with proximity. This indicates doping‐induced electronic homogenization, where coordination distortion imparts dopant‐like traits to nearby host atoms. Regularity and convergence were further validated in TM@TiO_2_, TM@ZrO_2_, and TM@SnO_2_ systems, highlighting universal dopant‐host electronic coupling (Figures S38–S40).

Subsequent analysis reveals a hierarchical pattern in both SHAP values and DFT‐calculated energies reflecting the competitive adsorption behavior of OH^*^ on complex surfaces. Within the *HESM* framework, OH adsorption is decomposed into three stages: (1) initial attraction to the global large sphere (***G‐***); (2) competition adsorption among doped and host atoms (***G‐A***); and (3) final localization at specific active sites: *D_TM_*, *H_M1_*, or *H_M2_* (***G‐A‐L***). For instance, in Rh@TiO_2_, the ***G‐*** dominates the absolute position of adsorption energy, while ***G‐A*** and ***G‐A‐L*** undergo one and two differentiations, respectively (Figure 5c). Expanding this analysis to TM@TiO_2_ (Figure 5d), as well as TM@ZrO_2_ and TM@SnO_2_ (Figures S41 and S42), the ***G‐*** class establishes the fundamental SHAP value curve, with ***A‐*** and ***L‐*** class contributions introducing incremental modifications. This results in three characteristically similar yet subtly distinct energy curves. Corresponding hierarchical splitting is observed in DFT‐calculated OH^*^ adsorption energies (Figure 5e, with analogous trends in TM@ZrO_2_ and TM@SnO_2_ systems in Figures S41 and S42). These findings validate the broad applicability of *HESM* across varying substrate systems (MO_2_), dopant elements (TM), and active site types (*D_TM_*, *H_M1_*, *H_M2_*). Furthermore, hierarchical splitting was analyzed in the O‐OH Gibbs free energy difference (ΔG_2_) for TM@TiO_2_ systems (Text S5 and Figure S43). While the energetics primarily depend on the M atom itself, the observed regularity and convergence remain governed by electronic and coordination effects.

To clarify competitive adsorption between *D_TM_* and *Host‐S* for intermediates, a conceptual framework is proposed. In hetero‐periodic systems, *D_TM_*/*Host‐S* with higher energy *d*‐states (e.g., 4*d*/3*d* transition metals) exhibit stronger OH^*^ adsorption. In homo‐periodic systems, activity follows a V‐shape trend: for *d* ^0^‐like host configurations (e.g., Ti, Zr), (Sn‐based systems are excluded due to the non‐transition metal character), OH^*^ favors *D_TM_* sites along the right arm of the V‐shape. A secondary *H_M1_*‐*H_M2_* competition mechanism, modulated by dopant‐induced *d*‐band center shifts, further regulates adsorption: positive shifts (e.g., V→Ti) enhance *H_M1_*‐site adsorption, whereas negative shifts (e.g., Cr→Ti) stabilize *H_M2_* sites (Figure S44).

Building on these findings, we attribute the high activity of Rh@MO_2_ *D_TM_* sites and Fe@ZrO_2_ *H_M1_* sites to synergistic global electronic effects and local coordination field distortions. PDOS analysis (Figure S45) confirms that the hybridization between M‐*d_xz_*, *d_z2_*, and *d_x2‐y2_* orbitals and OH‐*p* orbitals during adsorption, supported by orbital schematics and OH^*^ configurations. For Rh@MO_2_ *D_TM_* sites (Figures S46 and S47), inherent high activity is maintained despite slight variations in local coordination and charge retention, due to sharp peaks near the Fermi level. In Fe@ZrO_2_ (Figures S48 and S49), pyramidal coordination expands at *H_M1_*/*H_M2_* sites while reducing charge loss. Fe‐induced electronic restructuring enhances *d*‐orbital filling below E_f_, particularly in *d_xz_*/*d_z2_* orbitals. The *H_M1_* site exhibits multiple charge states near E_f_ that favor OH^*^ adsorption. Unlike traditional single electronic structure descriptors, *HESM* holistically represents multiscale effects in complex catalytic systems by incorporating hierarchical feature contributions.

## 3 Conclusions

In summary, this study develops the *HESM* that provides a simplified paradigm to study complex catalytic surfaces. By decoupling multiscale geometric‐electronic interactions into three distinct descriptors (***G‐***, ***A‐***, ***L‐***), the model establishes a quantitative framework linking atomic‐scale properties to overall reactivity, thereby addressing a critical challenge in heterogeneous catalysis. Notably, *HESM* enables descriptor‐level prediction of the adsorption energy splitting, where ***G‐*** class descriptors define the overall energy trend curve, while ***A‐*** and ***L‐*** class features provide local correction effects. This hierarchical contribution lays a theoretical foundation for the competitive adsorption behaviors of OH^*^. It also successfully elucidates the coexistence mechanism of two distinct active sites (Rh@MO_2_ *D_TM_* and Fe@ZrO_2_ *H_M1_*), clarifying the dominant role of electronic structure and the tuning effect of local coordination.

The model exhibits high extensibility, laying the foundation for its application across diverse catalytic systems and reaction classes. By integrating bulk electronic descriptors with structural parameters, it is readily adaptable not only to doped oxides but also to other catalyst families. Furthermore, its descriptor logic, based on key intermediates (e.g., OH^*^ for OER), can be extended to reactions like CO_2_ reduction and nitrogen fixation. *HESM*'s machine learning algorithm can further be compatible with deep neural networks, balancing high‐throughput data processing with interpretability. By incorporating additional hierarchical factors like solvation effects and applied potential, future work will bridge the simulation‐experiment gap, positioning *HESM* as a universal platform linking atomic‐scale coordination to macroscopic catalytic performance.

## 4 Experimental Methods

### 4.1 DFT Calculation

All spin‐polarized DFT calculations were performed using the Vienna Ab initio Simulation Package (VASP) [57, 58]. The Perdew–Burke–Ernzerhof (PBE) form of the generalized gradient approximation (GGA) was employed to describe the exchange‐correlation potentials [59]. The Hubbard U correction was applied to account for the strong correlations in the *d* electrons of transition metal oxides, with specific effective U values and their determination methods detailed in Text S6. The projector‐augmented wave (PAW) method was used to describe the electron‐ion interactions, with a plane‐wave cutoff energy set to 520 eV, and the zero‐damping DFT‐D3 method of Grimme was utilized to correct for van der Waals interactions [60]. The convergence criteria were set to forces below 0.02 eV Å^−1^ and energy differences below 10^−5^ eV per atom. A 3 × 3 × 1 Monkhorst‐Pack grid was employed for k‐point sampling to describe the supercell [61, 62], and a vacuum layer of 15 Å was added along the *z*‐direction to avoid interactions between periodic images. Thermodynamic stability was assessed using ab initio molecular dynamics (AIMD) simulations within a typical NVT ensemble, conducted over 12 ps with a time step of 2 fs at a temperature of 300 K. Additionally, the local chemical bonding properties of periodic systems were analyzed by calculating the crystal orbital Hamilton population (COHP), obtained by multiplying the Hamiltonian matrix with the corresponding DOS matrix. This analysis was further supported by the powerful LOBSTER tool [63], which enables visualization of COHP diagrams and evaluation of bond strength when using a plane‐wave basis set. Details of energy‐related calculations can be found in Text S7.

## Funding

This work was supported by the National Natural Science Foundation of China (Grant No. 12374061, No. 12404274), Zhejiang Provincial Natural Science Foundation of China (Grant No. LQN25A040020, No. LD24F040001).

## Conflicts of Interest

The authors declare no conflicts of interest.

## Supporting information




**Supporting File**: advs73083‐sup‐0001‐SuppMat.pdf.

## Data Availability

The data that support the findings of this study are available from the corresponding author upon reasonable request.
