# Supplementary material for: Hierarchical Embedded Sphere Model: An Interpretable ML‐Guided Multiscale Descriptor Engineering Decodes OER Activity on TM@MO2 Catalysts
Source: Adv Sci (Weinh). 2025 Dec 19;13(11):e18931. doi: 10.1002/advs.202518931 (PMC12931158; doi:10.1002/advs.202518931)
Supplement: Supplementary file 1 — Supporting File: advs73083‐sup‐0001‐SuppMat.pdf. [file ADVS-13-e18931-s001.pdf]

Supporting Information

Hierarchical embedded sphere model: An interpretable ML-guided multiscale descriptor engineering decodes OER activity on TM@MO_2 catalysts

Ziyuan Li, Shan Gao,* Yunhan Wang, Yueyu Zhang, Weichao Wang, Xiangmei Duan*

Supplementary Text

Text S1. Detailed Calculation of Machine Learning Feature Engineering

1. Features in the single-site catalytic model

According to the literatures, the traditional single-site catalytic model design in Fig. 2 should incorporate three distinct feature categories from Fig. S1: (1) intrinsic atomic features (labeled as AF), (2) electronic structural properties obtained from DFT calculations, and (3) simple descriptors derived from combinations of intrinsic properties.

❶ Atom's Feature	Definition
N_d	*d*-shell valence electrons of M
R_v	The van der Waals radius of M
I_M	The first ionization energy of M
X_M	Electronegativity of M
N_M	Electron affinity of M

DFT Feature	Definition
❷ M_e	Charge change of M after OH*
❸ CT_e	Charge transfer from metal oxides to intermediates
❹ $IC_{M\text{-}O}$	Bond strength between M and O in adsorption state
❺ ε_d	*d*-band center of M

Descriptor	Definition	Descriptor	Definition
❻ ψ_1	Composite index of charge transfer capacity with M as active center	❽ δ_{XT}	Global electronegativity difference between M and intermetiate
❼ ψ_2	Same as ψ_1, but with M-5O as active center	❾ δ_{IE}	Ionization energy difference

Figure S1. Three categories of features included in the traditional single-site catalytic model.

2. ***s***-type features

For the descriptors in Fig. S1, we selected the validated ψ_1, ψ_2, δ_{XT}, δ_{IE} from the literatures. [1] 错误!未找到引用源。 Their calculation formulas are as follows:

$$\psi = N_{\mathrm{d}}^{ac} \times \frac{\left|X_M^{ac} - X_O\right|}{\left|X_M^{ac} - X_O\right| + \left|X_M^{ac} - X_{sub}\right|} \begin{cases} \psi_1 : X_M^{ac} = X_M \\ \psi_2 : X_M^{ac} = X_{MO5} \end{cases} \tag{1}$$

$$X_{sub} = \frac{\left|\sum n_k \times X_{Mk} + n_O \times X_O\right|}{\sum n_k + n_O} \tag{2}$$

Here, N_d^{ac} is the valence electron count of the active M atom at the adsorption site; X_M^{ac}, X_O, X_{Mk} represent the electronegativities of this active M atom, its adjacent O atom, and other M atoms in the doped structure spatially distant from the active site, respectively; n_k, n_O denote the numbers of non-active M atoms and O atoms in the doped structure. The descriptors ψ_1, ψ_2 are constructed based on spatially defined active centers: the isolated M atom at the adsorption site and the local M-5O unit composed of this M atom and its neighboring O atom, where X_M, X_{MO5} correspond to the electronegativity of the active M atom and the average electronegativity of the M-5O unit, respectively. In summary, ψ_1 and ψ_2 are composite descriptors that evaluate charge transfer capability between the substrate and adsorbed oxygen, anchored by distinct active centers (M atom or M-5O unit).

$$\delta_{XT} = \overline{X_M} - \overline{X_{int}} = \begin{cases} \overline{X_M} = \dfrac{X_M + \sum n_i X_i / N}{2} \\ \overline{X_{int}} = \dfrac{\sum n_j X_j}{n_{int}} \end{cases} \tag{3}$$

$$\delta_{IE} = \frac{1}{N} \sum (IE_M - IE_i) \tag{4}$$

Here, X_M, X_i and X_j denote the electronegativities of the M atom at the adsorption site, its nearest-neighbor atoms, and atoms within the adsorbed intermediate, respectively; n_i, n_j, n_{int} and N represent the counts of each atom type in the nearest-neighbor environment, each atom type in the adsorbed intermediate, the total atoms in the intermediate, and the total nearest-neighbor atoms, respectively; IE_M and IE_i are the first ionization energies of the M atom and its nearest-neighbor atoms. In summary, δ_{XT} and δ_{IE} are descriptors quantifying the global electronegativity difference between the M atom and the adsorbed intermediate, and the average ionization energy difference between the M atom and its adjacent atoms, respectively.

3. ***L***-class features

For the ***s***-type feature descriptors, according to the definition, the same doped surface should have two different sets of values corresponding to the dopant atom D_{TM} and the host metal atom *Host-S*, which are labeled as $\boldsymbol{z^D}$-(ψ_1, ψ_2, δ_{XT}, δ_{IE}) and $\boldsymbol{z^H}$-(ψ_1, ψ_2, δ_{XT}, δ_{IE}), respectively. Since our study focuses on the OER catalytic performance at distinct sites on doped surfaces, it is essential to resolve site-specific characteristics in the feature representation. Furthermore, the introduction of dopant atoms breaks the symmetry of the pristine surface, leading to varying degrees of distortion in the local coordination environment. To account for this effect, we introduce a geometric weight factor w_L and an adaptive factor w_1 to construct the ***L***-class features. Here, we use ***L***-ψ_1 as an example:

$$L\text{-}\psi_{1M} = \begin{cases} (1-w_1)\times z^D\text{-}\psi_1 + w_1\times\left[\dfrac{1}{e^{wLM}}\times z^D\text{-}\psi_1 + \dfrac{e^{wLM}-1}{e^{wLM}}\times z^H\text{-}\psi_1\right],\ M = TM \\ (1-w_1)\times z^H\text{-}\psi_1 + w_1\times\left[\dfrac{1}{e^{wLM}}\times z^D\text{-}\psi_1 + \dfrac{e^{wLM}-1}{e^{wLM}}\times z^H\text{-}\psi_1\right],\ n = M1,\ M2 \end{cases} \quad (5)$$

$$w_{LM} = \mathrm{d}_M / \mathrm{d}_c,\ M = TM,\ M1,\ M2 \quad (6)$$

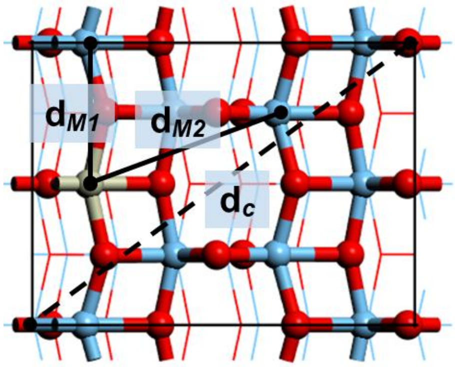

Figure S2. Parameters related to the geometric structural weight w_L.

Here, as shown in the Fig. S2, d_M denote the geometric distances between specific sites (e.g., D_{TM}, H_{M1}, H_{M2}) and the doping site, while d_c represents the unit cell diagonal length. It is important to note that d_{TM} is always zero, while the other distances vary for each surface. For simplicity, we take the values corresponding to the distances between sites on the undoped surface, with the specific values listed in the Table S1. The adaptive factor w_1 is initially assigned a value of 0.05, which serves to quantify the degree of local structural responsiveness of the catalyst to a specific reaction intermediate. Physically, w_1 functions as a normalization coefficient that describes how local coordination environments adapt to perturbations induced by either dopant incorporation or intermediate adsorption. This well-defined role enables the *HESM* framework to capture the system-specific geometric sensitivity as the catalyst, while simultaneously preserving the overall consistency of the model when applied across different oxide families.

4. ***G***-class and ***A***-class features

After replacing the ***s***-type features with ***L***-class features, although the feature structure is sufficient to distinguish each example, there are still some issues: ① The ***h***-type features are only used to describe the Ti, Zr, and Sn atoms, and the excessive repetition of the data leads to a decrease in the accuracy of the machine learning model; ② The ***d***-type features, due to nearly two-thirds of

the examples being adsorbed on the *Host-S* atoms, result in some data being invalid. Here, we still abstract the single-site model into the *Hierarchical Embedded Sphere Model* (*HESM*) by introducing the geometric weight factor w_G and the adaptive factor w_2. Taking ***G***-N_d as an example:

$$G\text{-}N_d = \frac{\left(h\text{-}N_d \times N_{1H} + d\text{-}N_d \times N_{1D}\right) + w_2 \times w_G \times \left(h\text{-}N_d \times N_{2H} + d\text{-}N_d \times N_{2D}\right)}{N_{1H} + N_{1D} + N_{2H} + N_{2D}} \tag{7}$$

$$w_G = \mathrm{d_s} / \mathrm{d_{s2}} \tag{8}$$

As shown in the Fig. S3, we divide the surface-exposed metal atoms into 4 surface atoms (unsaturated M atoms, which are shown with adjacent O atoms using the precise molecular model CPK) and 4 sub-surface atoms (saturated M atoms, which are represented by the ball-and-stick model). In the equation, N_{1H}, N_{1D}, N_{2H}, and N_{2D} represent the number of *Host-S* atoms and D_{TM} atoms in the most-surface and sub-surface, respectively. For this study, these values are fixed as 3, 1, 4, and 0. d_s and d_{s2} represent the distances to the closest surface and sub-surface *Host-S* atoms to D_{TM}, respectively. In the Fig. S3, d_{s2} should actually be the distance between D_{TM} and the *Host-S* atom in the same position of the adjacent unit cell on the left side. To simplify the operation, the values are taken as the distances between corresponding sites on the undoped surface, with specific values provided in the Table S2. The adaptive factor w_2 is initially assigned a value of 0.5, which is used to quantify the global structural responsiveness of the catalyst to a specific reaction intermediate. Analogous to w_1, w_2 acts as a normalization coefficient that describes how the catalyst's overall lattice structure and electronic environment adapt to perturbations induced by either intermediate adsorption or dopant incorporation. Here, after characterizing the catalytic model as the *HESM*, we use the ***G***-class features to describe the intrinsic properties of the overall large sphere.

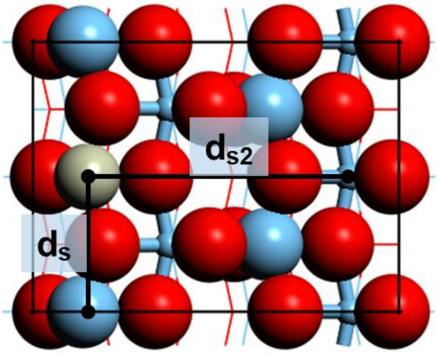

Figure S3. Parameters related to the geometric structural weight w_G.

Additionally, the ***A***-class features are used to describe the intrinsic properties of the embedded small sphere at the adsorption site. Taking ***A***-N_d as an example:

$$A\text{-}N_d^s = \begin{cases} d\text{-}N_d, s = D_{TM} \\ h\text{-}N_d, s = H_{M1}/H_{M2} \end{cases} \tag{9}$$

Text S2. Screening metal oxides and analysis TM@MO_2 stability

The anatase phase, recognized as the most thermodynamically stable form of TiO_2, was selected as the primary substrate for OER electrocatalytic activity. To establish the comparative benchmarks, we extended our analysis to structurally analogous MO_2 oxides (M = Fe, Zr, Nb, Ir, Sn) retrieved from the Materials Project database (structural details provided in Fig. S4). Stability screening via Pourbaix diagram analysis revealed severe limitations for FeO_2 (complete instability) and NbO_2 (narrow stability window), promoting their exclusion from subsequent studies. For the remaining candidates (TiO_2, ZrO_2, IrO_2, SnO_2), optimized lattice parameters exhibited excellent agreement with database references, with deviations < 1.0% (Table S3).

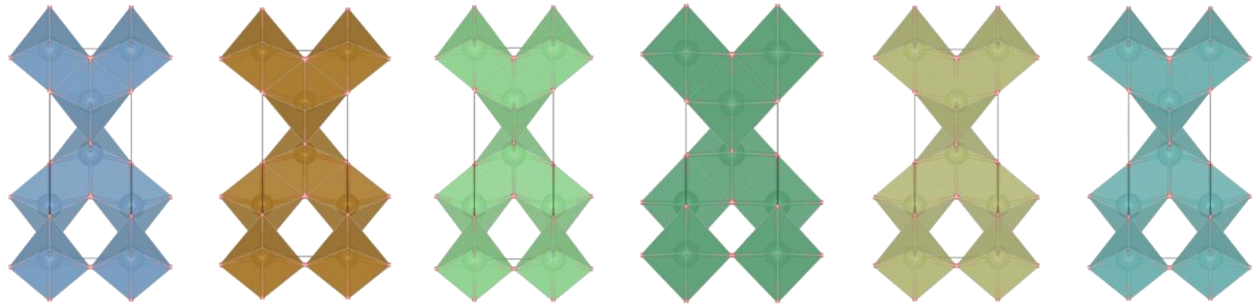

Figure S4. Structural diagrams of six anatase-phase MO_2 (M = Ti, Fe, Zr, Nb, Ir, Sn).

The anatase (101) surface, widely employed in computational and experimental catalysis due to its low surface energy and thermodynamic resilience, served as our prototype. [3] We constructed 1×2×1 supercell models for the MO_2 (101) surface, with the bottom atomic layers fixed to mimic bulk-constrained relaxation (Fig. S5).

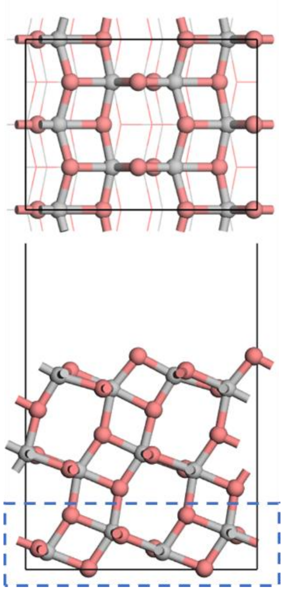

Figure S5. Top and side views of the MO_2 (101) surface, where the atoms inside the blue dashed box are fixed during the structural relaxation process to simulate the slab structure.

As illustrated in Fig. 1, the MO_2(101) surface presents multiple adsorption sites prone to occupation by OER intermediates (H^*, O^*, or OH^*) derived from water activation. DFT calculations revealed site-specific preferences: O^* and OH^* preferentially absorb on undercoordinated M atoms (coordination number = 5), while H^* occupies protruding O atoms (Fig. S6). Surface Pourbaix diagrams (Fig. S7), employing the Nernst equation, [46] elucidated potential-dependent coverage. For TiO_2, the surface exhibit H^*-dominated coverage at potentials below −0.06 V, a bare surface between 1.23 and 1.73 V, and O^*-covered regions at potentials above 1.73 V. For ZrO_2 and SnO_2, bare surfaces persist within the potential range of 1.23-1.55 V and 1.23-1.79 V, respectively. IrO_2 exhibits continuous OH^* coverage across the potential range of 0.22-1.62 V, suggesting irreversible surface reconstruction under these conditions. Given IrO_2's untypical behavior, we focused on computational effects on TiO_2, ZrO_2, and SnO_2.

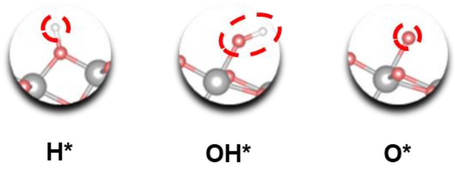

Figure S6. Different configurations of the MO_2 surface under OER working conditions.

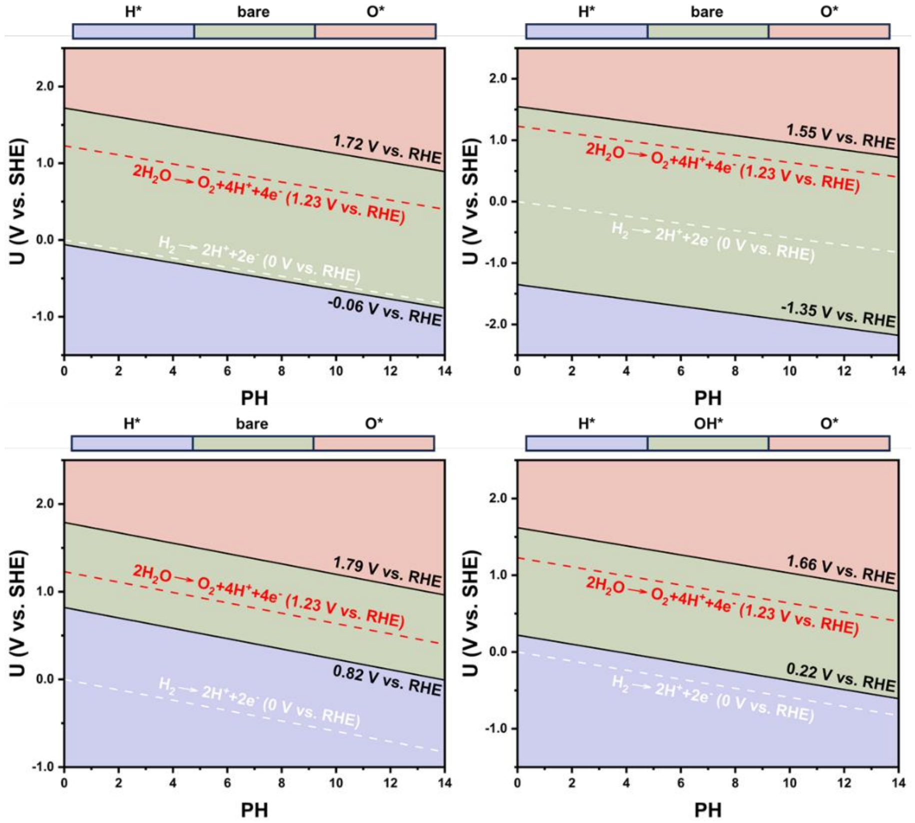

Figure S7. Surface Pourbaix diagram of the MO_2 surface (M=Ti, Zr, Sn, Ir).

To enhance their intrinsic OER activity, we engineered single-atom transition metals (TMs, Fig. S8) doping into MO_2 (101) surfaces, systematically evaluating 78 candidates through high-throughput DFT screening. As shown in Fig. 1B, TM doping at the D_{TM} site among the four exposed metal atoms on the surface breaks the original symmetry, making the three *Host-S* atoms inequivalent under the dopant influence. Consequently, three unique adsorption sites were modeled per doped surface. This framework generated 234 distinct reaction configurations.

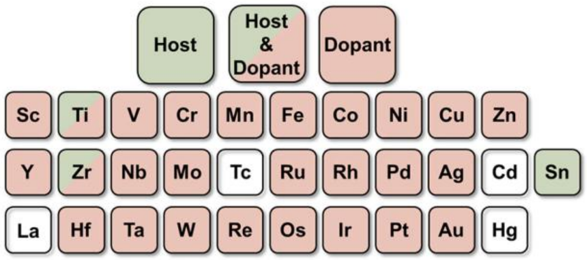

Figure S8. Elemental selection matrix: Periodic table highlighting host metals (green: Ti, Zr, Sn-based oxides) and dopant metals (red: 26 TM candidates).

Structural feasibility and stability were assessed though doping binding energy (E_b) and cohesive energy (E_c) calculations ($E_b/E_c > 0.5$), [78] with detailed stable structures in Figs. S18-S20. As illustrated in Fig. S21, all TM-doped MO_2 systems satisfy this criterion, demonstrating robust structural integrity under thermodynamic equilibrium. Complementary AIMD simulations were performed on representative systems—TM@TiO_2 (TM = Mn, Rh, Pd, Ag), TM@ZrO_2 (TM = Co, Zn, Ru, Rh), and TM@SnO_2 (TM = Ti, Zr, Rh, Hf)—to validate dynamic stability. Over 12 ps trajectories at 300 K (Figs. S22-S24), total energy fluctuations remained within 1.0 eV of equilibrium values, while temperature oscillations were constrained to < 50 K. No bond rupture or lattice reconstruction was observed, confirming the doped configurations' energetic resilience. These findings align with experimental precedents demonstrating TM feasibility on anatase (101) surfaces via solvothermal synthesis and In-situ generation methods, substantiating the practical realizability of our designed catalysts. [9, 10]

Text S3. Electronic structure analysis and the relationship with ΔG_1

Focusing on ΔG_1 and OH^* adsorption configurations, we conducted detailed electronic structure analyses. Integrated ICOHP and Bader charge analyses (Figs. S31-S33) demonstrate a negative correlation, that is ΔG_1 inversely relates to $-ICOHP_{M\text{-}O}$ (bond strength) and CT_e (charge transfer). TM@TiO_2's anomaly manifests in that OH charge transfer-ΔG_1 correlation inversion occurs beyond $CT_e > 0.4\ e$, attributed to strong Ti-O covalency. The positive correlation of $-ICOHP_{M\text{-}O}$ with CT_e, and negative correlation with M_e highlight competing ionic/covalent interactions, exemplifying characteristic dual bonding effects. For *Host-S* sites, ΔG_1 vs. $-ICOHP_{M\text{-}O}$ exhibits a cascade pattern with maximum bond strength. Optimal Rh configurations consistently reside at the midpoint of these relationships, suggesting that only appropriate OH^* adsorption strength ensures optimal OER activity.

Text S4. Training and Validation of the *HESM* Model

To evaluate the practical performance and physical validity of the *HESM* (including ***G***-, ***A***-, ***L***-class, and ***c***-type descriptors), we integrated it with DFT-derived geometric, electronic, and adsorption energy data.

First, a correlation matrix was constructed between ***c***-type and ***L***-class descriptors (Fig. S9). With the exception of $IC_{M\text{–}O}$, all ***c***-type features exhibit strong correlations with ***L***-class descriptors (|corr| > 0.5), and ε_d reaching 0.8. To reduce dependency on DFT-derived properties and improve model transferability, ***c***-type features were excluded from the final model. Although internal correlations among ***L***-class descriptors exceeded 0.7, each captures distinct physical aspects of local coordination. For example, ***L***-ψ_2 reflects the charge-transfer capability among the adsorbate, active center, and substrate at an M-5O site, while ***L***-δ_{IE} quantifies the mean ionization energy difference between the adsorption site and its coordinating atoms. Given their clear physical interpretations, the ***L***-class descriptors were retained.

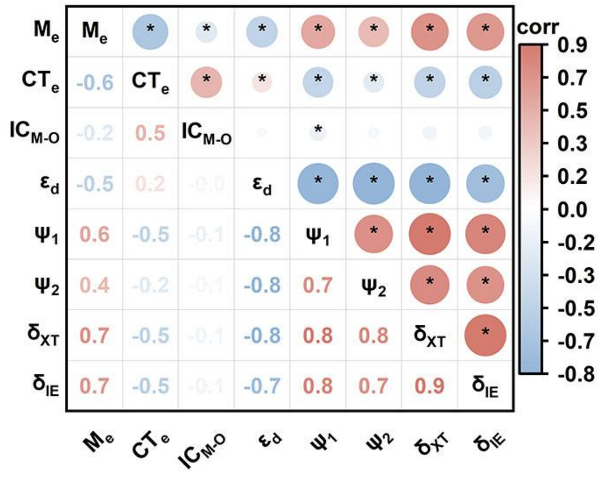

Figure S9. Pearson correlation coefficient heatmap between ***c***-type and ***L***-class features. Bubble size and color intensity reflect correlation strength, with asterisk denoting statistically significant relationships.

Subsequently, the feature space was constructed using ***G***-, ***A***-, and ***L***-class descriptors, with ΔG_{OH*} as the target variable. The dataset was split into training and test sets at an 8:2 ratio. As illustrated in Fig. S10, the distributions across host materials (TiO_2, ZrO_2, SnO_2), dopant periods

(3d, 4d, 5d), and adsorption sites (D_{TM}, H_{M1}, H_{M2}) are well-balanced (≈33% ± 4%), which ensures the dataset's representativeness and minimizes potential bias.

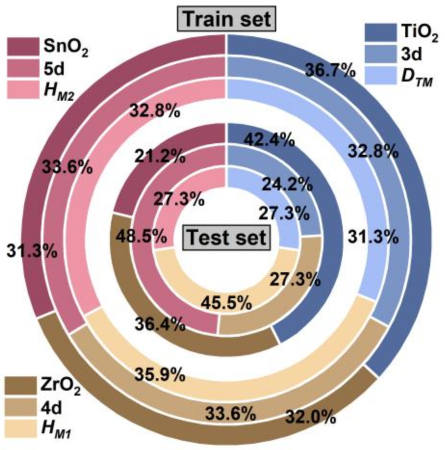

Figure S10. Circular plot of hierarchical feature distributions in the training and test sets.

We compared eight regression algorithms (GBR, XGBR, LGBM, KNR, RFR, SVR, CatBoost, and AdaBoost) using different descriptor combinations (***h***-, ***d***-, ***L***-types vs. ***G***-, ***A***-, ***L***-class). As shown in Fig. 4a, LGBM (R^2 = 0.92) and GBR (R^2 = 0.90) performed best when ***G***-class descriptors were excluded. After including ***G***-class descriptors, XGBR (R^2 = 0.95) and LGBM (R^2 = 0.92) achieved the highest predictive accuracy.

To improve physical interpretability, we performed a parameter scan over the adaptive factors w_1 and w_2, with w_1 ranging from 0.05 to 0.25 and w_2 from 0.3 to 0.7. The results (Fig. S11) show that model performance improves as the doping effect increases. The optimal parameter set (w_1 = 0.25, w_2 = 0.7) gives R^2 = 0.96, with ***G***-, ***A***-, and ***L***-class contributions accounting for 52.4%, 32.4%, and 15.2%, respectively (Fig. 4b). Among individual descriptors, ***G***-N_d (26.8%) and ***G***-R_V (18.0%) exhibit the strongest influence.

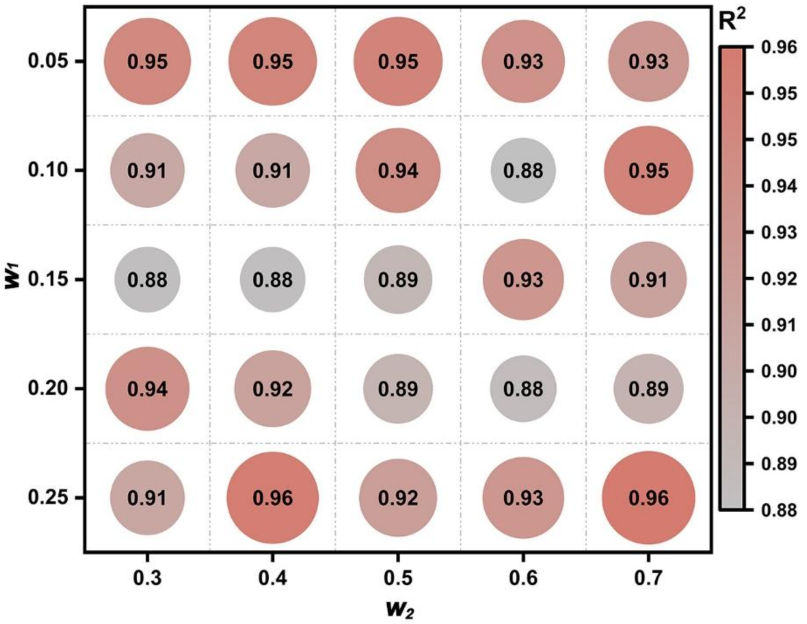

Figure S11. Test results of the adaptive factors w_1 and w_2.

A sensitivity analysis with ±20% perturbation around physically values (Fig. S12) verified the model's robustness, with R^2 staying above 0.85. ***G***-class descriptors remained dominant in feature importance (>0.33, Fig. S13). SHAP-based rankings (Figs. S14-S15) further enhanced interpretability, identifying ***G***-N_d as the top influential feature, followed by ***G***-R_V, ***L***-ψ_2, and ***L***-δ_{XT}.

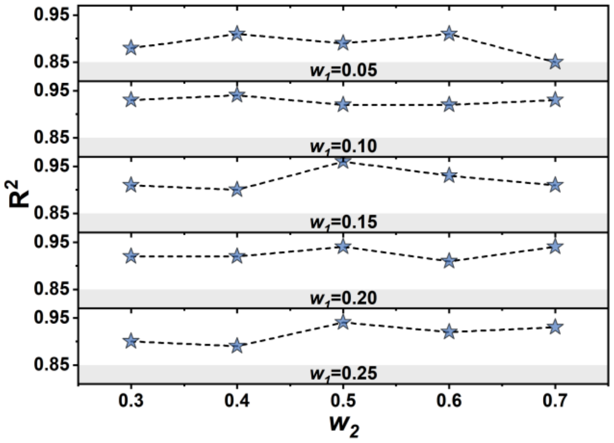

Figure S12. Sensitivity of model performance (R^2) to adaptive factors w_1 and w_2.

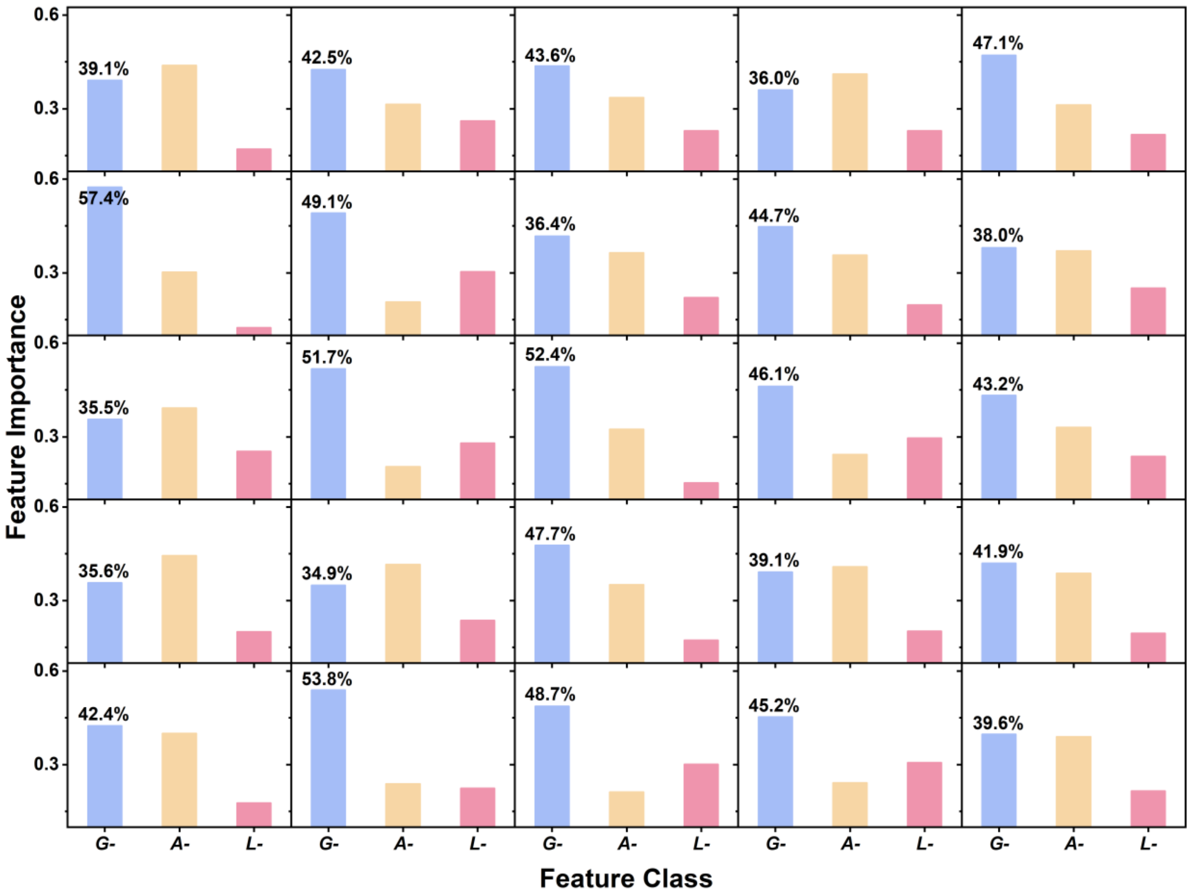

Figure S13. Sensitivity of feature importance to adaptive factors w_1 and w_2.

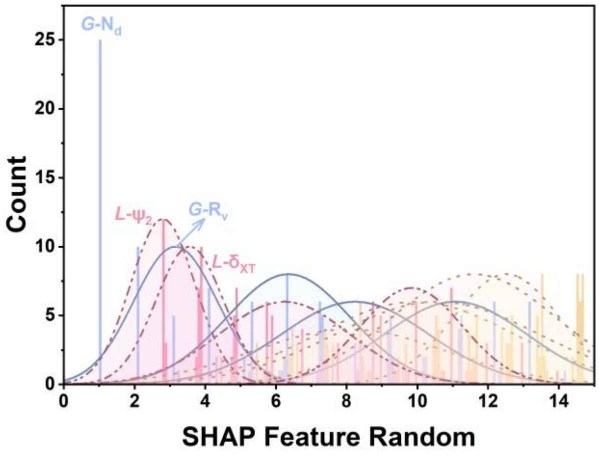

Figure S14. SHAP-based feature ranking statistical chart from the sensitivity analysis of adaptive factors w_1 and w_2.

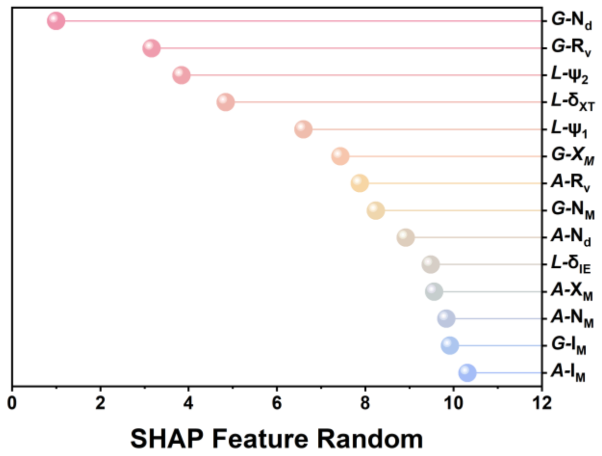

Figure S15. SHAP-based feature rankings: frequency-weighted average from the sensitivity analysis of adaptive factors w_1 and w_2.

To assess the model's extrapolation capability, we conducted two leave-one-out validation tests: (i) training on TM@TiO_2 and TM@SnO_2, and testing on TM@ZrO_2; (ii) training on all systems except Rh@MO_2, and testing on Rh@MO_2. As summarized in Fig. S16, case (i) yielded an R^2 value of 0.76—partly attributed to the out-of-range adsorption energies on ZrO_2—while case (ii) yielded an R^2 value of 0.89, confirming the model's strong predictive reliability within known chemical regions.

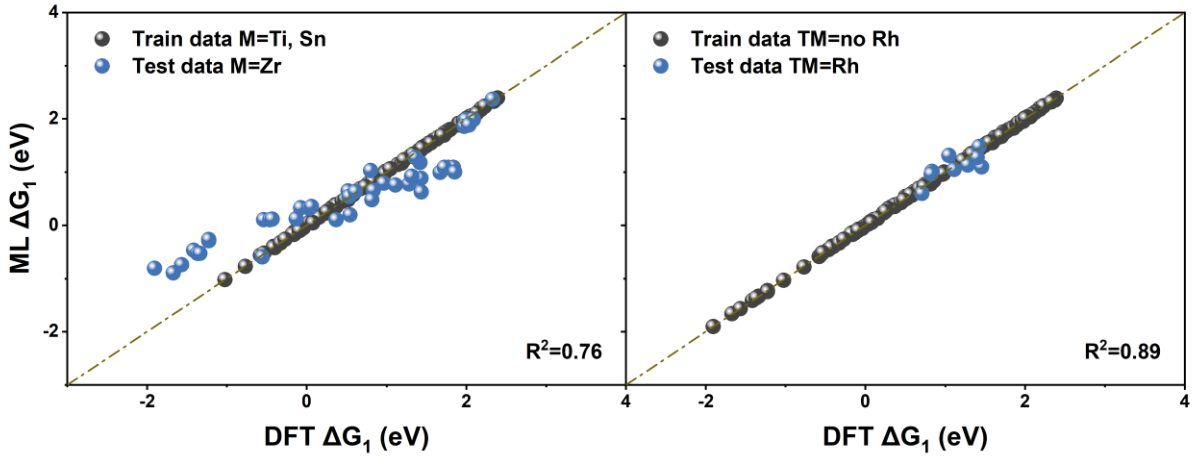

Figure S16. Results of leave-one-out cross-validation with one host (ZrO_2) and one dopant (Rh) exclude.

Finally, to demonstrate the extensibility of the framework, we applied the *HESM* model to predict the adsorption free energy of O^* (ΔG_{O^*}). Under high, medium, and low adaptability settings (w_1 = 0.25, w_2 = 0.7; w_1 = 0.15, w_2 = 0.5; w_1 = 0.05, w_2 = 0.3), the corresponding R^2 values were 0.72, 0.79, and 0.89, respectively (Fig. S17). This trend indicates that O^* adsorption

relies more strongly on the intrinsic properties of the adsorption site—a physically consistent result that underscores the generality and scalability of the *HESM* framework.

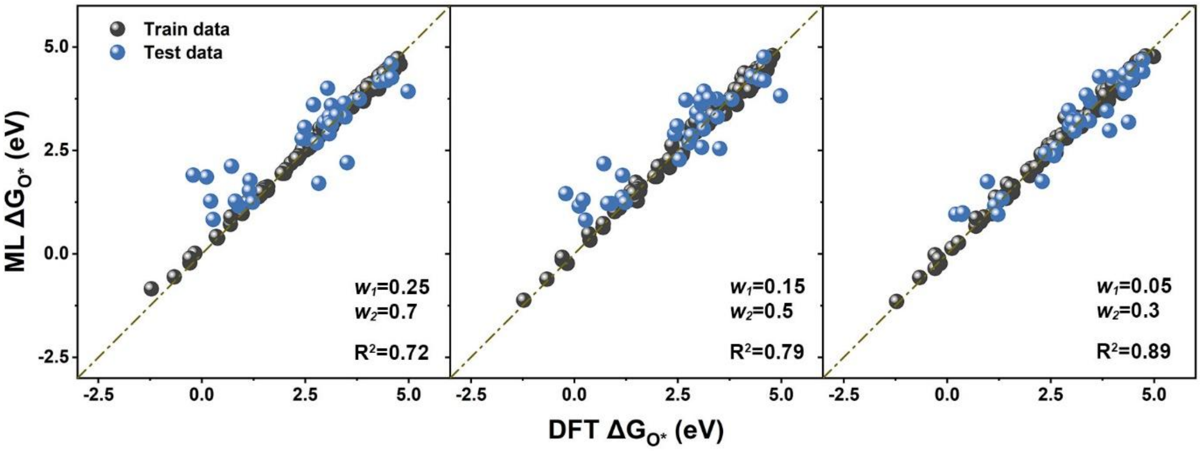

Figure S17. Results of extrapolation validation across high, medium, and low adaptability configurations.

Text S5. Hierarchical energy splitting of the DFT ΔG_2

We plotted the free energy variations for ΔG_2 on TM@TiO_2 (Fig. S43). The fluctuations in free energy at *Host-S* sites were observed to be less pronounced compared to those at D_{TM} sites, except early TMs (***d***-N_d = 1, 2). This conclusion aligns with the work of Kirsten T. Winther's hydroxyl adsorption model, [11] which highlights that the O-OH energy difference is markedly less sensitive to surface configurations than isolated OH adsorption energies. While dopant-centric studies dominate catalysis research, *Host-S* sites retain significant potential for high-performance OER systems. Synergistic dopant effects enable *Host-S* modulation to achieve favorable energetics, enhancing performance—as demonstrated by the exceptional activity of Fe@ZrO_2's H_{M1} site.

Text S6. Calculation of Hubbard effective U values

We used the linear response method (LSDA) provided on the VASP official website to calculate the Hubbard effective U values for each M atom in the bulk. Combined with commonly used values from the literature, since we only consider U_{eff} when performing electronic structure calculations, we determine the final U_{eff} value based on the calculated bandgap. According to literatures, the experimental bandgap value for the anatase TiO_2 configuration is 3.2 eV, and the U_{eff} value for Ti's 3*d* electrons is typically set to 3.3 eV in calculation.[12] We obtained a U value of 5.0 eV for Ti using the LSDA method. We compared band gap calculations using PBE, PBE+U_1 (3.3 eV), PBE+U_2 (5.0 eV), and HSE06 hybrid functionals, and the results are shown in the Fig. S50. The band gap relationship is as follows: experimental value (3.2 eV) ~ HSE06 value (3.28 eV) > PBE+U_2 (2.78 eV) > PBE+U_1 (2.51 eV) > PBE (2.06 eV). Therefore, we finally decided to adopt the U value calculated by the LSDA method. The U_{eff} values for Zr and Sn atoms were also calculated from their respective bulk structures, while the U_{eff} values for dopant atoms were determined by calculating the U value for individual dopant atoms on the surface.

Text S7. DFT calculation method

The binding energy (E_b) between the dopant and substrate is calculated to evaluate the thermodynamic stability of potential catalysts, while the cohesive energy (E_c) and the E_b/E_c ratio are computed to examine the aggregation behavior of dopant atoms on the substrate. The calculation formulas for E_b and E_c are as follows: [7]

$$E_b = E_{TM@MO2} - E_{TM\_s} - E_{MO2\_v} \quad (10)$$

$$E_c = E_{TM\_b}/n - E_{TM\_s} \quad (11)$$

Where, $E_{TM@MO2}$, E_{TM_s}, E_{MO2_v}, and E_{TM_b} represent the total energies of TM@MO_2, an isolated TM atom in vacuum, the MO_2 (101) substrate with a single surface vacancy, and a bulk TM crystal, respectively. n denotes the number of TM atoms in the TM crystal. A more negative E_b value corresponds to a higher probability of experimental synthesis, with $E_b/E_c > 0.5$ indicates an increased tendency for TM atoms to exhibit atomic segregation on the substrate.

The adsorption energies of the intermediates can be defined by the following formulas:

$$\Delta E_{OH*} = E_{OH*} - E_{*} - (E_{H2O} - 1/2\, E_{H2}) \quad (12)$$

$$\Delta E_{O*} = E_{O*} - E_{*} - (E_{H2O} - E_{H2}) \quad (13)$$

$$\Delta E_{OOH*} = E_{OOH*} - E_{*} - (2E_{H2O} - 3/2\, E_{H2}) \quad (14)$$

Here, E_*, E_{OH*}, E_{O*}, and E_{OOH*} are the total energies of the bare catalyst substrate and surfaces adsorbed with OH^*, O^*, and OOH^* species, respectively. E_{H2O} and E_{H2} are the total energies of H_2O and H_2 molecules in gas phases.

As is well known, the OER, as the half-reaction at the anode in water electrolysis, typically involves four electron-proton transfer steps. In electrochemistry, the OER in an acidic solution can be summarized as:

$$2H_2O \rightarrow O_2 + 4H^+ + 4e^- \quad (15)$$

Specifically, the fundamental reaction steps include:

$$H_2O + * \rightarrow OH* + H^+ + e^- \quad (16)$$

$$OH* \rightarrow O* + H^+ + e^- \quad (17)$$

$$H_2O + O* \rightarrow OOH* + H^+ + e^- \quad (18)$$

$$OOH* \rightarrow * + O_2 + H^+ + e^- \quad (19)$$

For which, * represents an active site on the bare catalyst surface, and OH^*, O^*, and OOH^* represent three different catalytic intermediates. Based on these reaction steps, the changes in Gibbs free energy $\Delta G_i (i = 1,\ 2,\ 3,\ 4)$ can be obtained using the following expression:

$$\Delta G_i = \Delta E + \Delta ZPE - \mathrm{T}\Delta S + \Delta G_U + \Delta G_{pH} \quad (20)$$

Where, ΔE is the adsorption energy of the adsorbed intermediates obtained through DFT calculations. ΔZPE and ΔS are the changes in zero-point energy and entropy contributions, respectively. $\Delta G_U = -eU$, where U is the electrode potential. $\Delta G_{pH} = k_B T ln 10 \times pH$.

For a given electrocatalyst, its theoretical overpotential can be evaluated using the method described in reference. [13]

$$\eta = max\{\Delta G_1,\ \Delta G_2,\ \Delta G_3,\ \Delta G_4\}/e - 1.23\ V \quad (21)$$

References

1. Xue, Z. Tan, R. Wang, et al., "A novel tetragonal T-C_2N supported transition metal atoms as superior bifunctional catalysts for OER/ORR: From coordination environment to ratio nal design," *Journal of Colloid and Interface Science* (2023): 149-158, https://doi.org/10.1016/j.jcis.2023.07.128
2. Zhu, Q. Gu, Y. Liang, X. Wang, X. and J. Ma, "A machine learning model to predict CO_2 reduction reactivity and products transferred from metal-zeolites," *ACS Catalysis* (2022): 12336-12348, https://doi.org/10.1021/acscatal.2c03250

3. Qiu, L. Fang, K. Li, et al., "Array of single crystalline anatase TiO_2 nanotubes with significant enhancement of photoresponse," *Progress in Natural Science-Materials International* (2021): 536-540, https://doi.org/10.1016/j.pnsc.2021.06.003
4. Dickens, C. F. Kirk, C. and J. K. Nørskov, "Insights into the electrochemical oxygen evolution reaction with *ab initio* calculations and microkinetic modeling: Beyond the limiting potential volcano," *The Journal of Physical Chemistry C* (2019): 18960-18977, https://doi.org/10.1021/acs.jpcc.9b03830
5. Liu, H. Jia, X. Cao, A. Wei, L. D'agostino, C. and H. Li, "The surface states of transition metal X-ides under electrocatalytic conditions," *The Journal of Chemical Physics* (2023): 124705, https://doi.org/10.1063/5.0147123
6. Yang, W. Jia, Z. Zhou, B. Wei, L. Gao, Z. and H. Li, "Surface states of dual-atom catalysts should be considered for analysis of electrocatalytic activity," *Communications Chemistry* (2023): 6, https://doi.org/10.1038/s42004-022-00810-4
7. Liu, J. Wang, S. Tian, et al., "Screening of silver-based single-atom alloy catalysts for NO electroreduction to NH_3 by DFT calculations and machine learning," *Angewandth Chemie-International Edition* (2025): e202414314, https://doi.org/10.1002/anie.202414314
8. Liu, X. Hupalo, M. Wang, et al., "Growth morphology and thermal stability of metal islands on graphene," *Physical Review B* (2012): 081414, https://doi.org/10.1103/PhysRevB.86.081414
9. Epifani, M. Kaciulis, S. Mezzi, et al., "Rhodium as efficient additive for boosting acetone sensing by TiO_2 nanocrystals: Beyond the classical view of noble metal additives," *Sensors and Actuators B-Chemical* (2020): 128338, https://doi.org/10.1016/j.snb.2020.128338
10. Bai, X. Li, Z. Fang, et al., "Adsorption and gas sensitive behavior of Co^{2+} doped TiO_2 (101) crystal planes," *Ceramics International* (2024): 23429-23440, https://doi.org/10.1016/j.ceramint.2024.04.065
11. Comer, B. M. Bothra, N. Lunger, et al., "Prediction of O and OH adsorption on transition metal oxide surfaces from bulk descriptors," *ACS Catalysis* (2024): 5286-5296, https://doi.org/10.1021/acscatal.4c00111
12. Shao G, "Red shift in manganese- and Iron-doped TiO_2: A DFT+U analysis," *Journal of Physical Chemistry C* (2009): 6800-6808, https://doi.org/10.1021/jp810923r
13. Man, I. C. Su, H. Calle-Vallejo, et al., " Universality in oxygen evolution electrocatalysis on oxide surfaces," *ChemCatChem* (2011): 1159-1165, https://doi.org/10.1002/cctc.201000397

Supplementary Figures

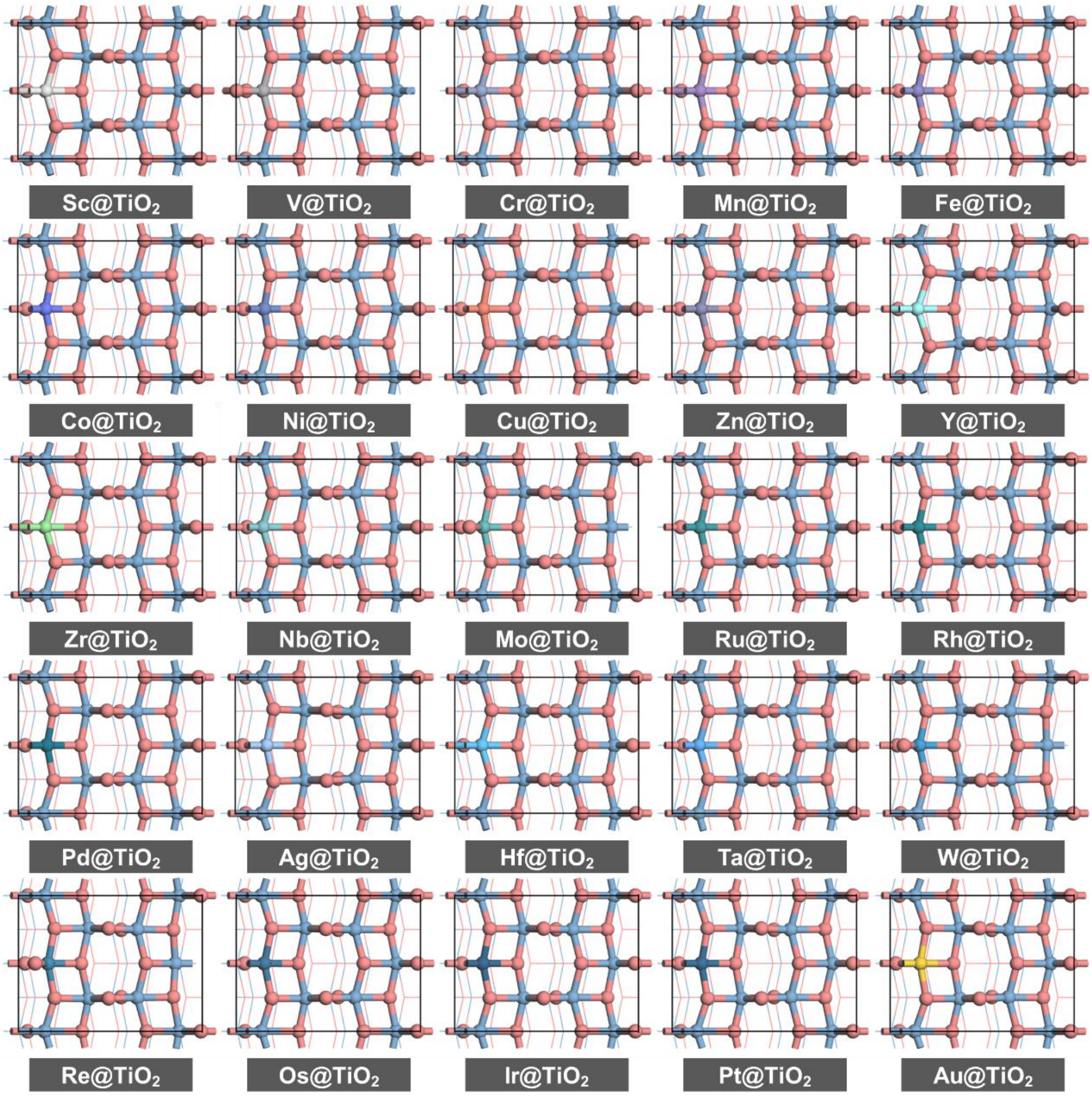

Figure S18. Optimized structures of TM@TiO_2.

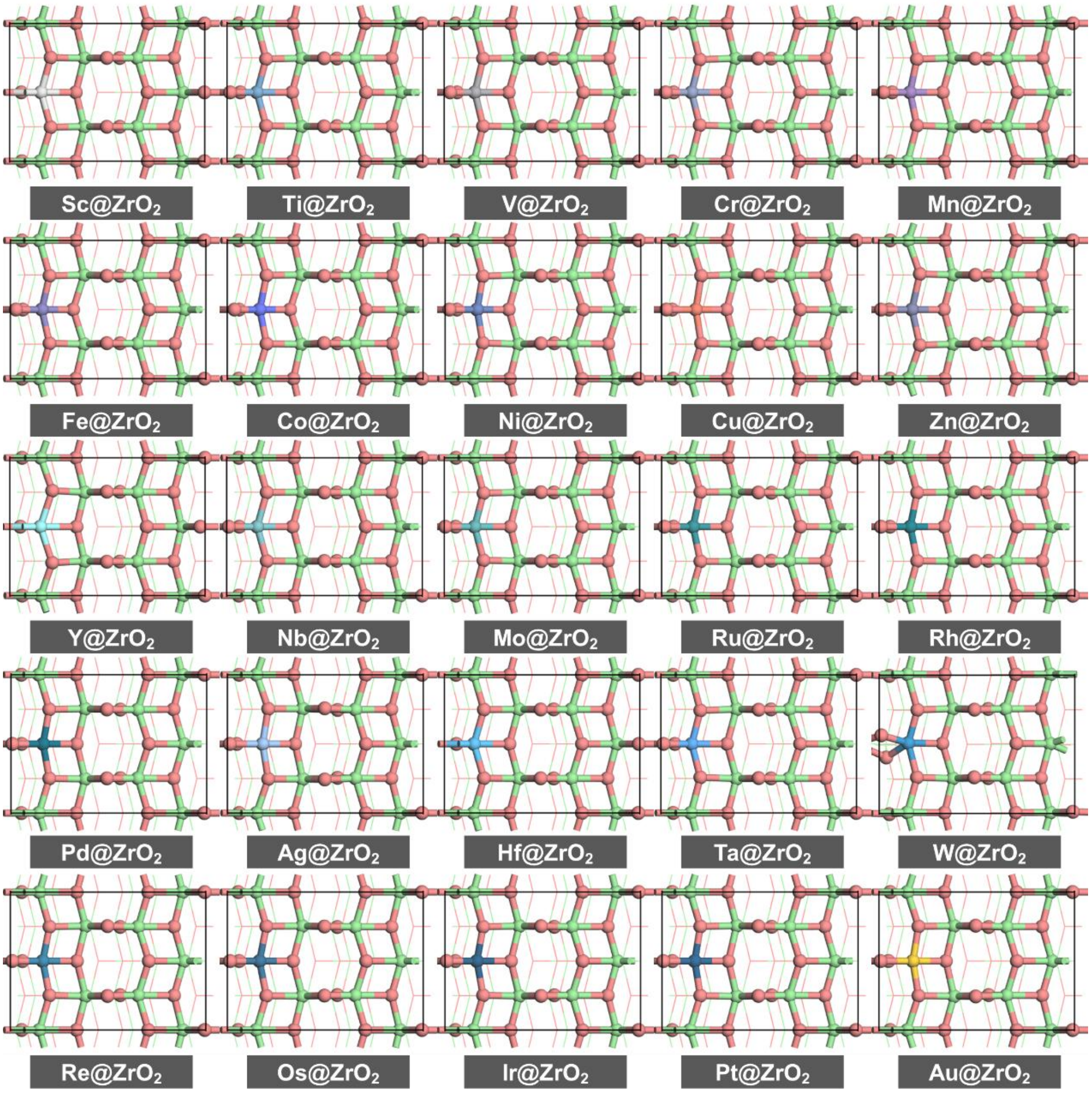

Figure S19. Optimized structures of TM@ZrO_2.

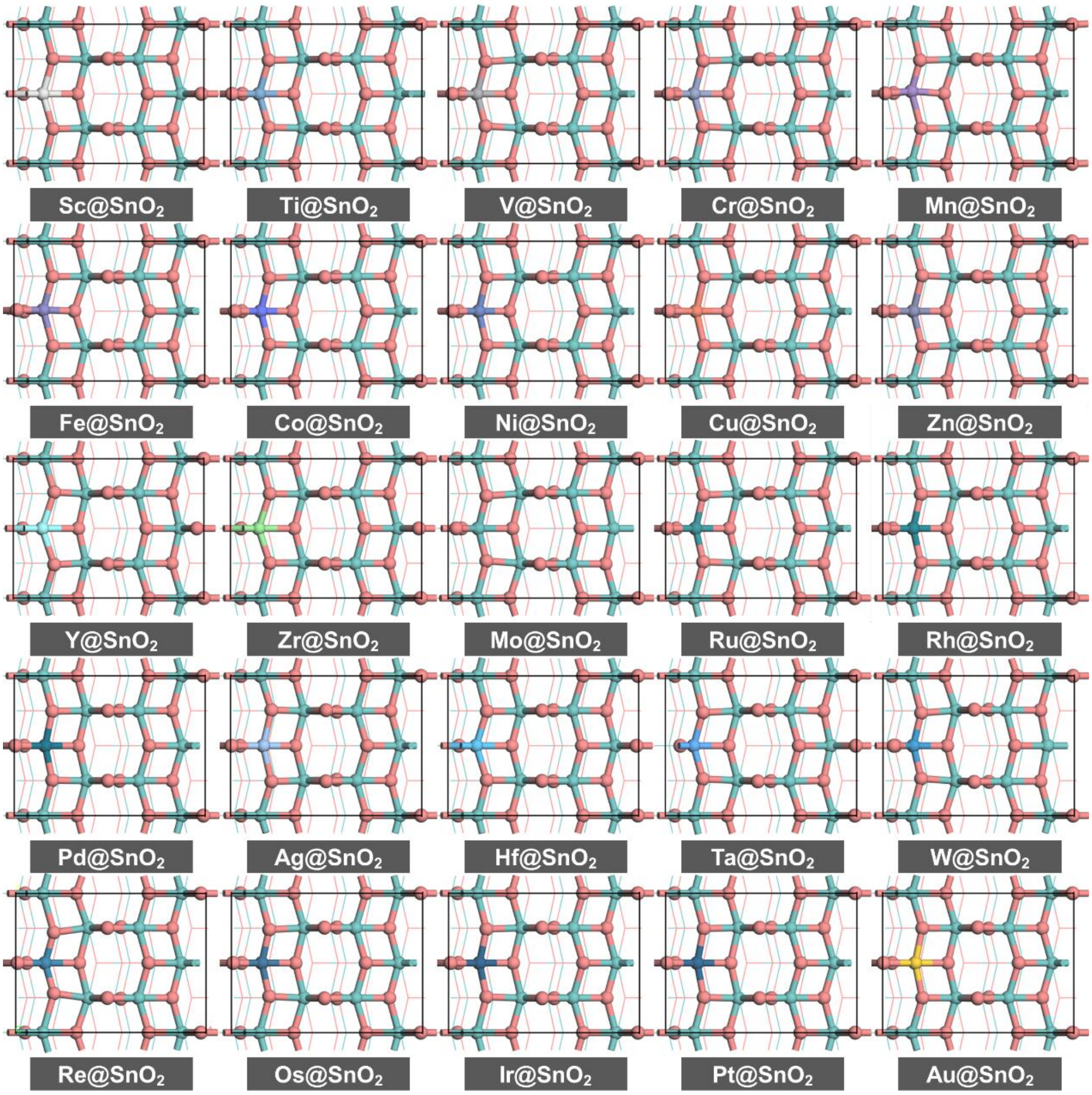

Figure S20. Optimized structures of TM@SnO_2.

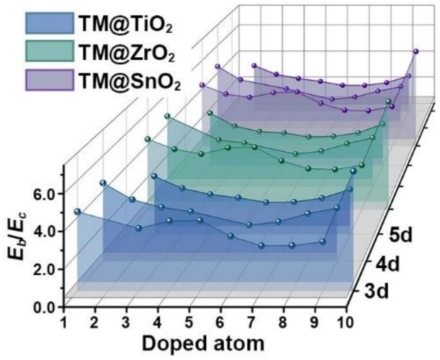

Figure S21. Dopant-induced stability metrics: E_b/E_c correlation map for TM doped MO_2 systems.

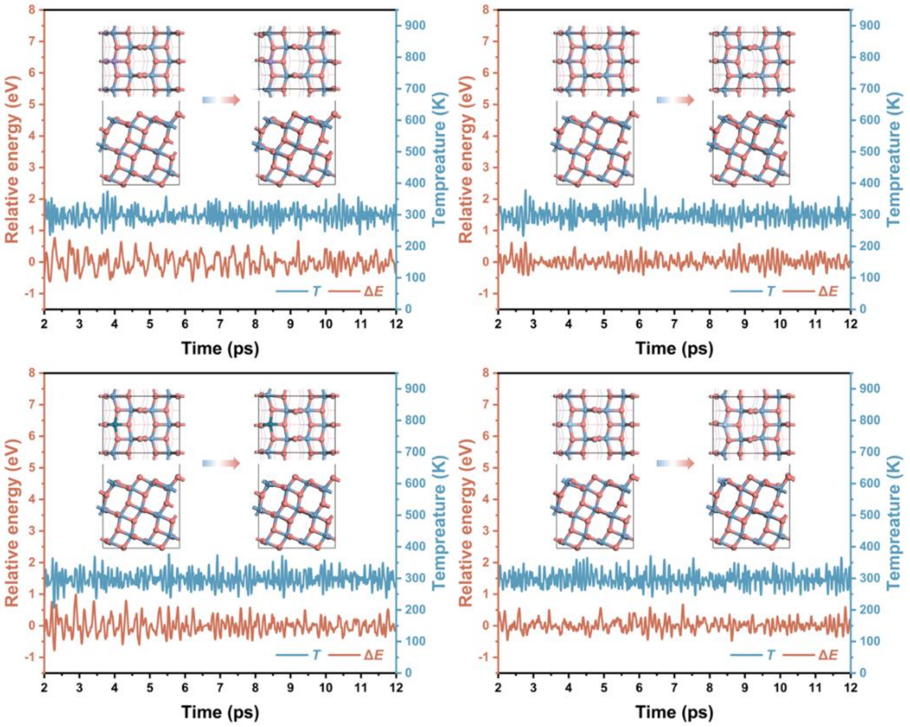

Figure S22. AIMD results of TM@TiO_2 (TM = Mn, Rh, Pd, Ag).

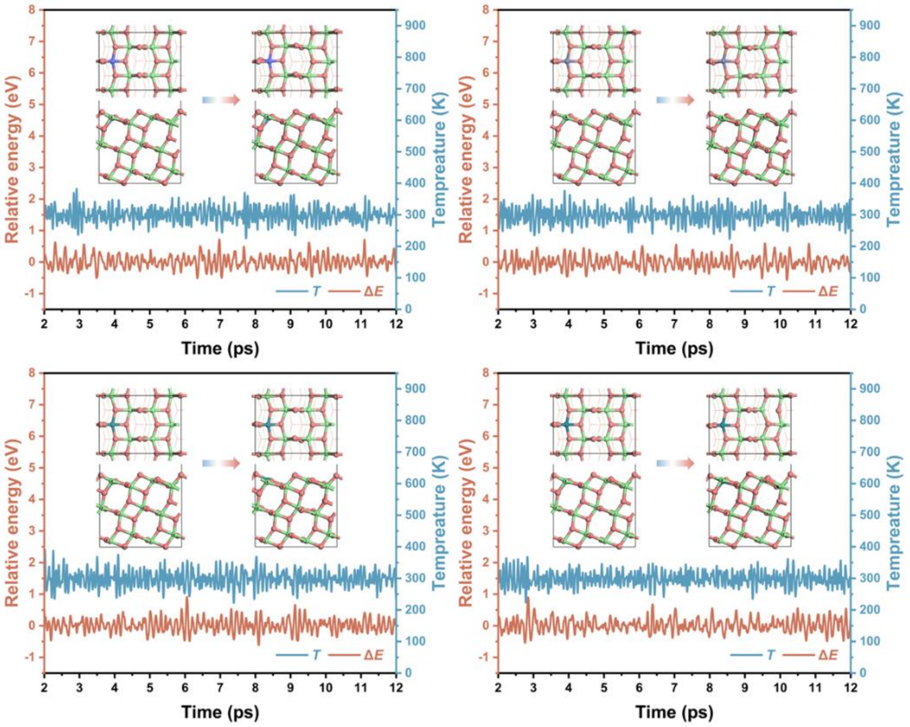

Figure S23. AIMD results of TM@ZrO_2 (TM = Co, Zn, Ru, Rh).

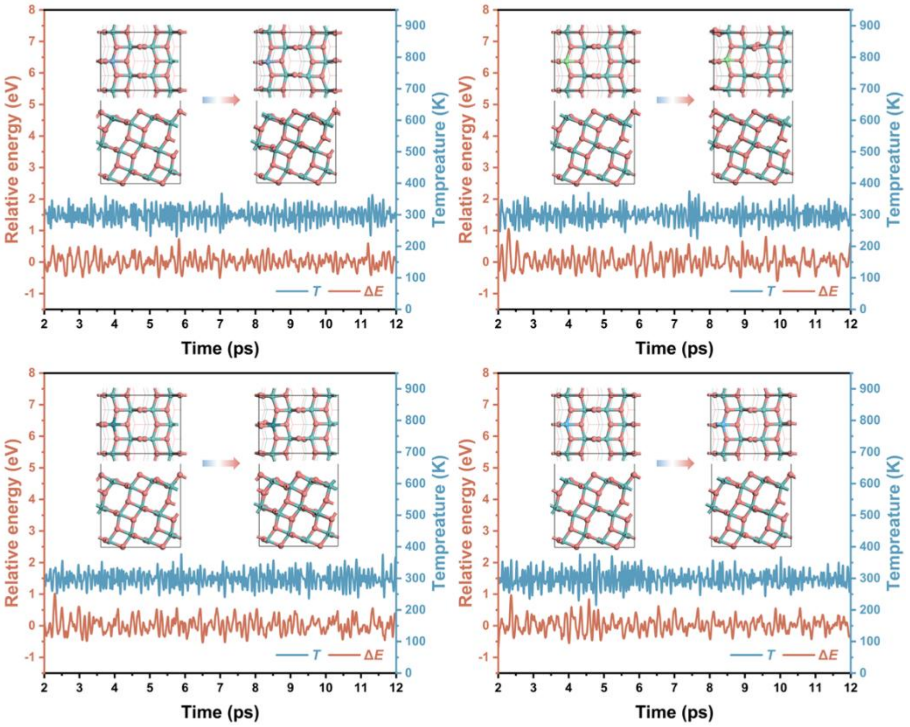

Figure S24. AIMD results of TM@SnO_2 (TM = Ti, Zr, Rh, Hf).

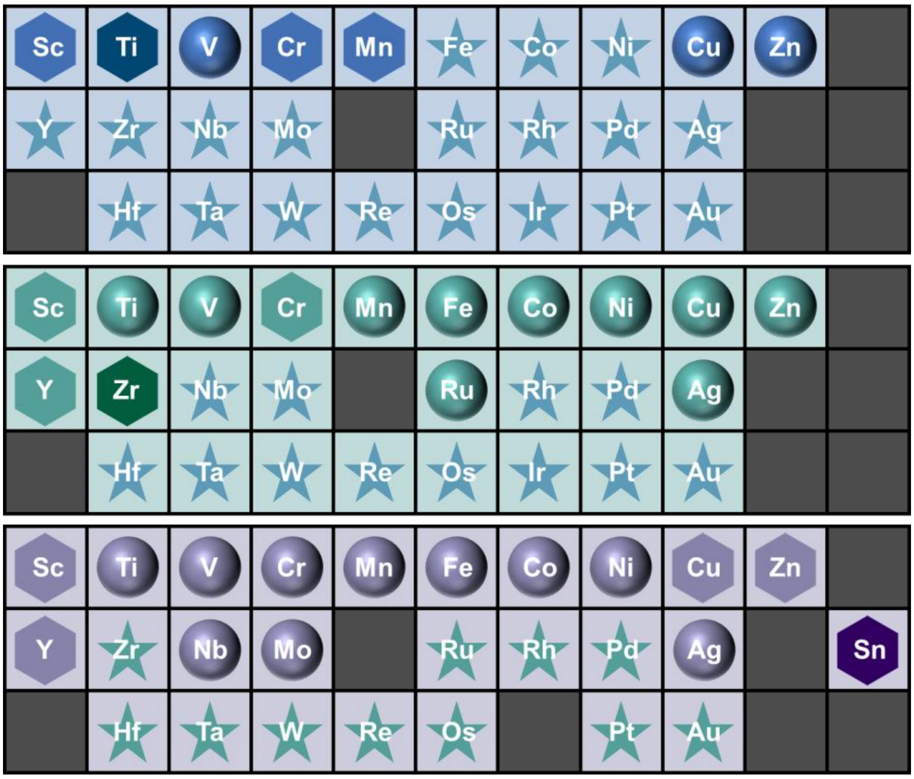

Figure S25. OH^* adsorption site results on TM@MO_2, where star symbols represent adsorption at D_{TM} sites, circle symbols represent adsorption at H_{M1} sites, and hexagon symbols represent adsorption at H_{M2} sites. Here, we assume that the reactions on the pristine MO_2 surface occur at the H_{M2} sites.

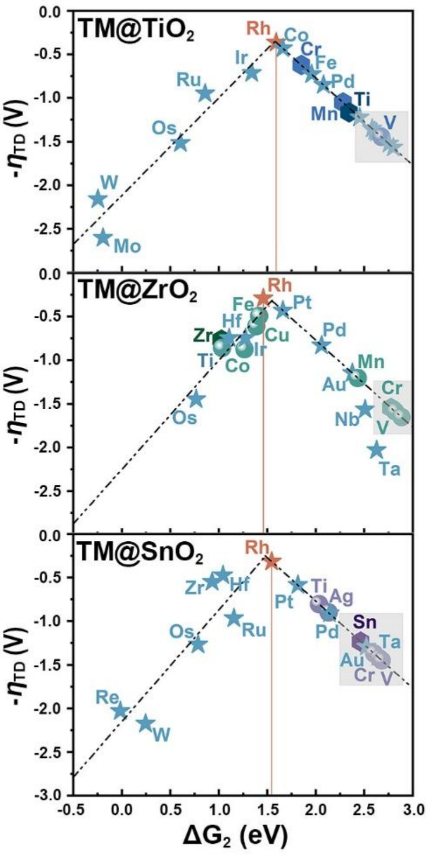

Figure S26. OER activity volcano plots for TM@MO$_2$ (where blue, green, and purple markers represent TiO_2, ZrO_2 and SnO_2 surfaces, respectively; dark blue stars indicate adsorption at D_{TM}, circles denote adsorption at H_{M1}, and hexagons denote H_{M2}, red star symbols indicate the optimal active sites for each substrate system).

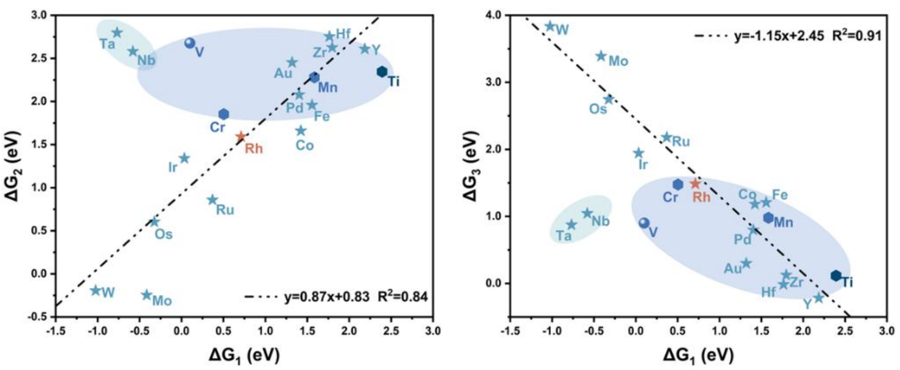

Figure S27. Linear scaling relationships of the OER reaction in the TM@TiO_2 system.

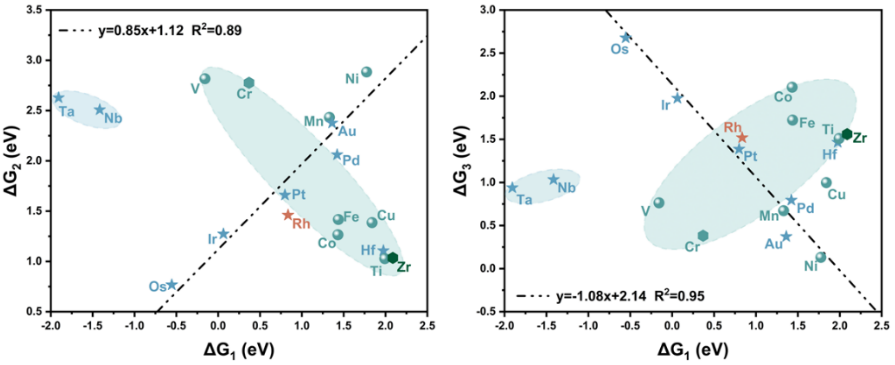

Figure S28. Linear scaling relationships of the OER reaction in the TM@ZrO_2 system.

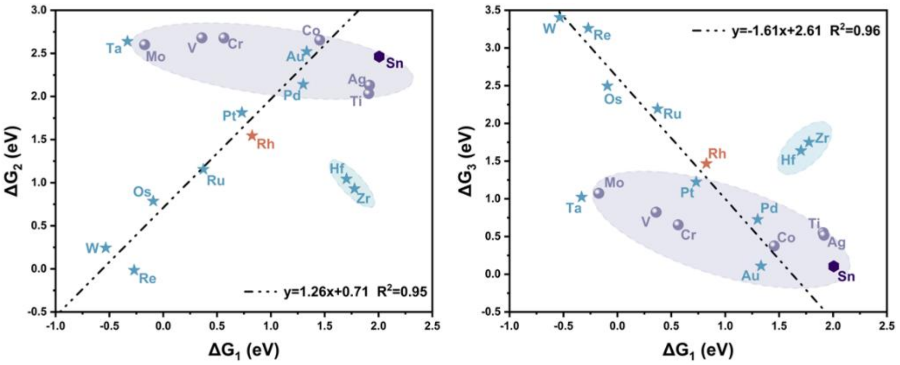

Figure S29. Linear scaling relationships of the OER reaction in the TM@SnO_2 system.

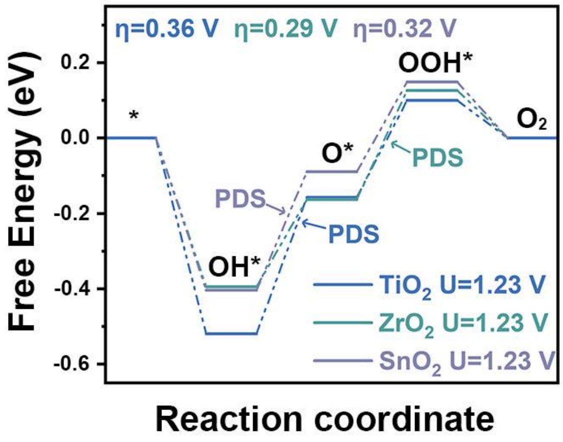

Figure S30. OER reaction free energy step plots for the best catalysts in each system.

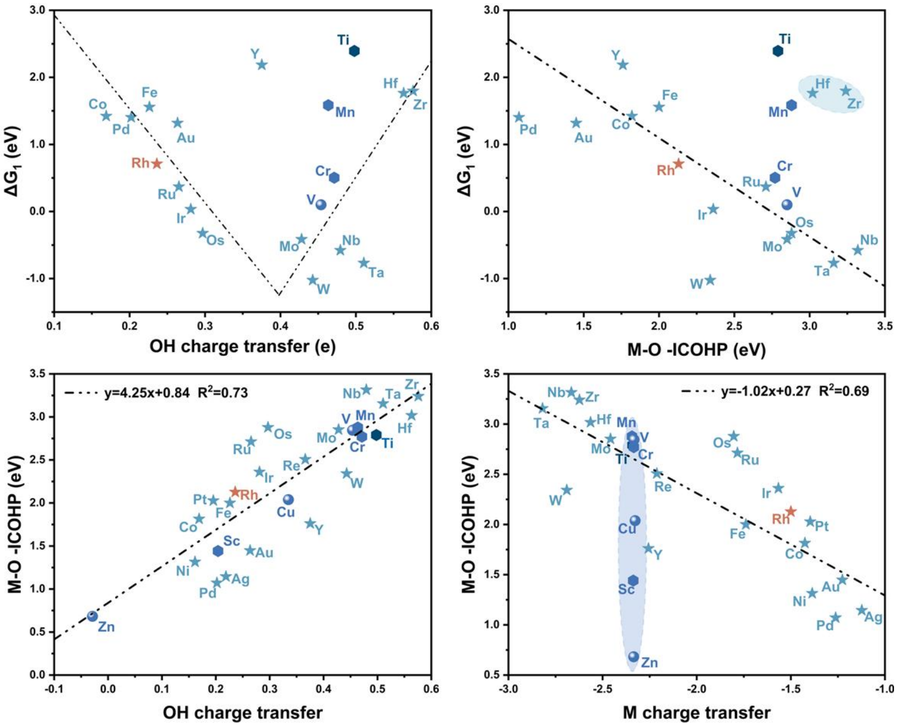

Figure S31. Four sets of linear relationships in the TM@TiO_2 system.

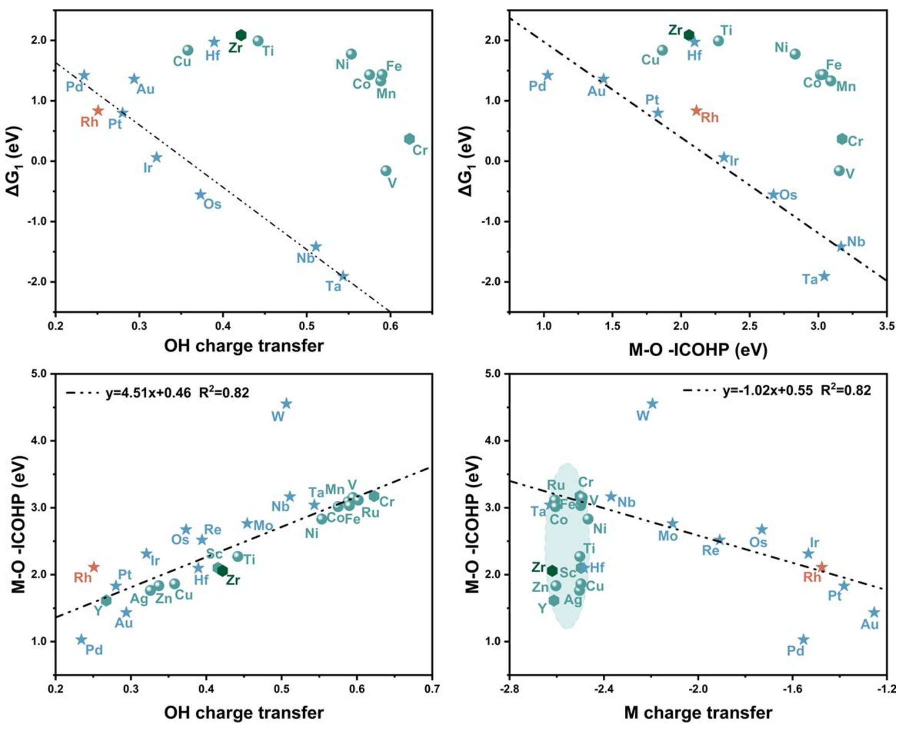

Figure S32. Four sets of linear relationships in the TM@ZrO_2 system.

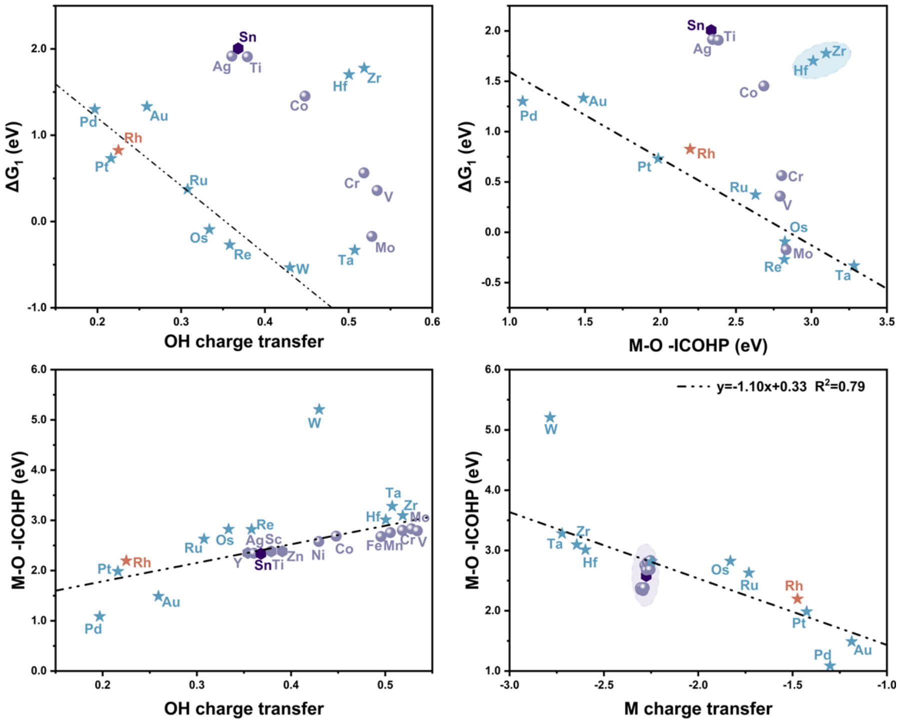

Figure S33. Four sets of linear relationships in the TM@SnO_2 system.

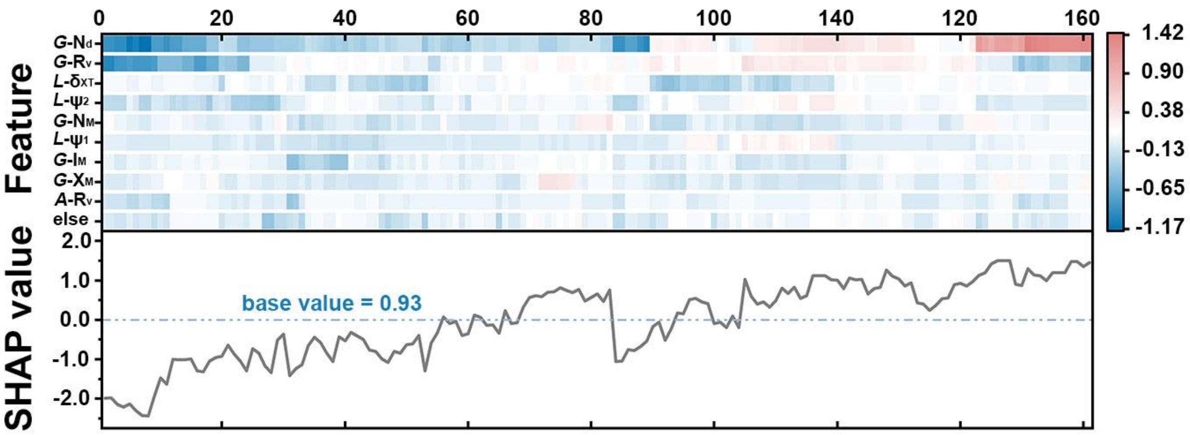

Figure S34. Heatmap of SHAP values for 14 features across 161 catalytic systems, ranked by feature importance (top to bottom). Overlaid gray trajectory traces the cumulative SHAP contributions per sample.

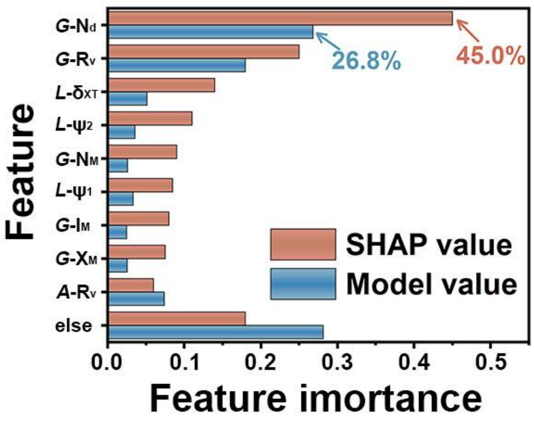

Figure S35. Two-dimensional comparison of feature importance (red: SHAP global importance value; blue: feature gain weight based on tree models).

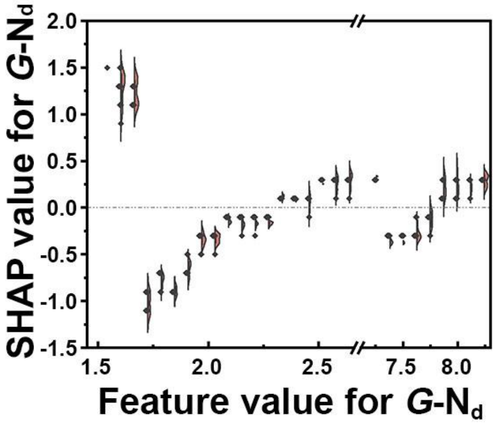

Figure S36. SHAP dependence distribution of ***G***-N_d.

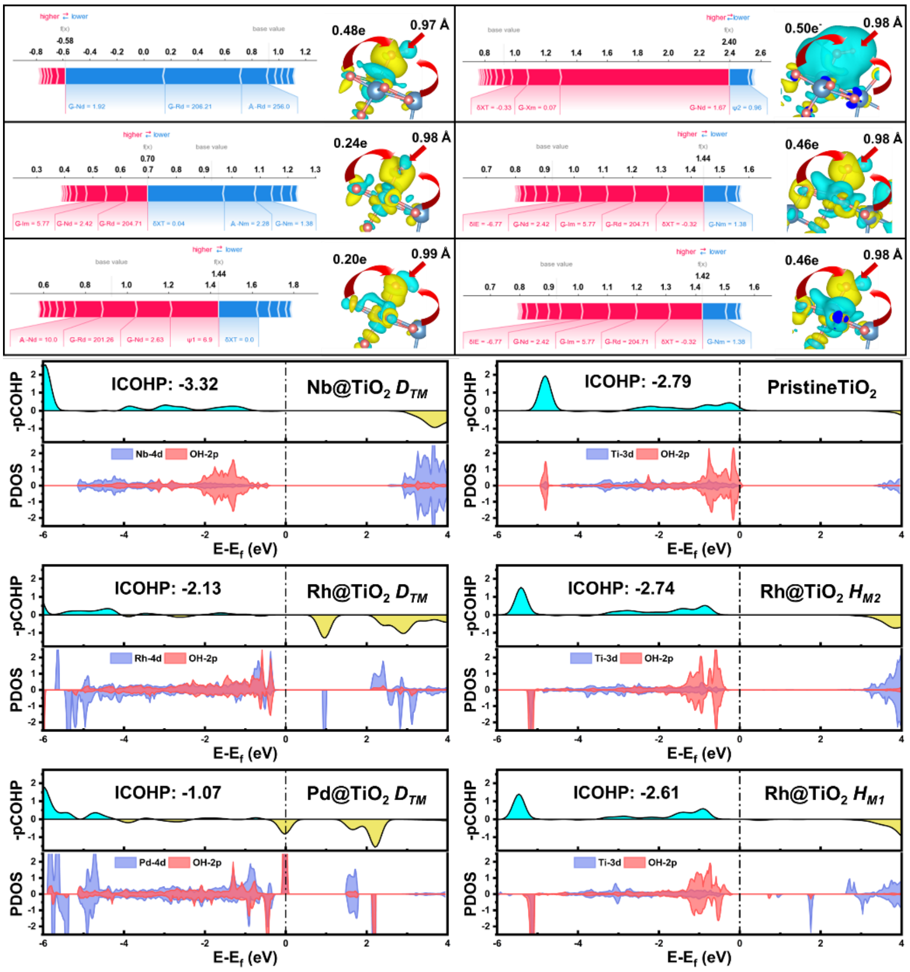

Figure S37. The SHAP force diagrams and electronic structure analyses of the D_{TM} sites on TM@TiO_2, different sites on Rh@TiO_2, and the Ti atoms on pristine TiO_2.

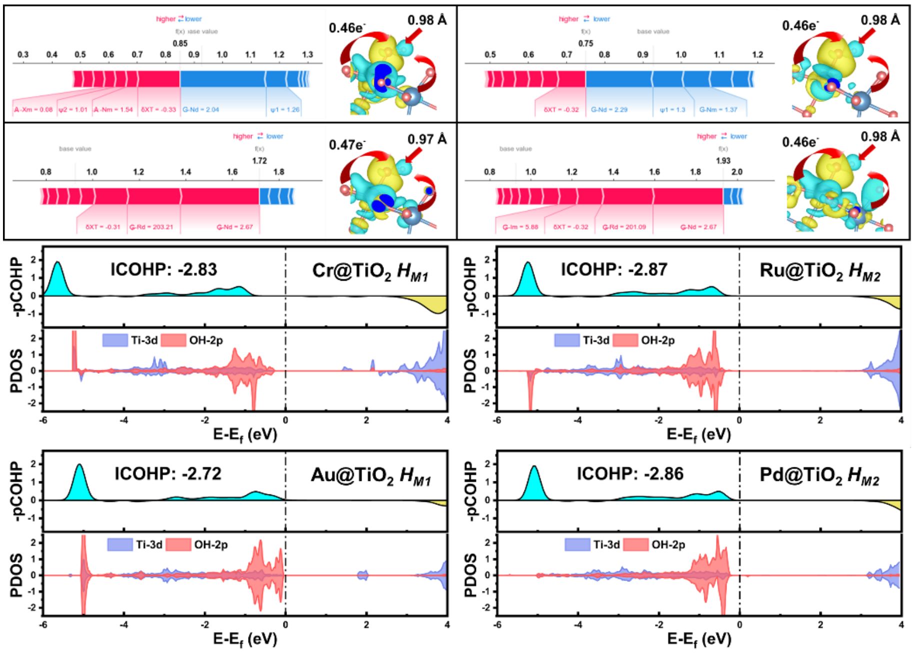

Figure S38. SHAP force plots and electronic structure analysis of H_{M1} and H_{M2} sites on $TM@TiO_2$.

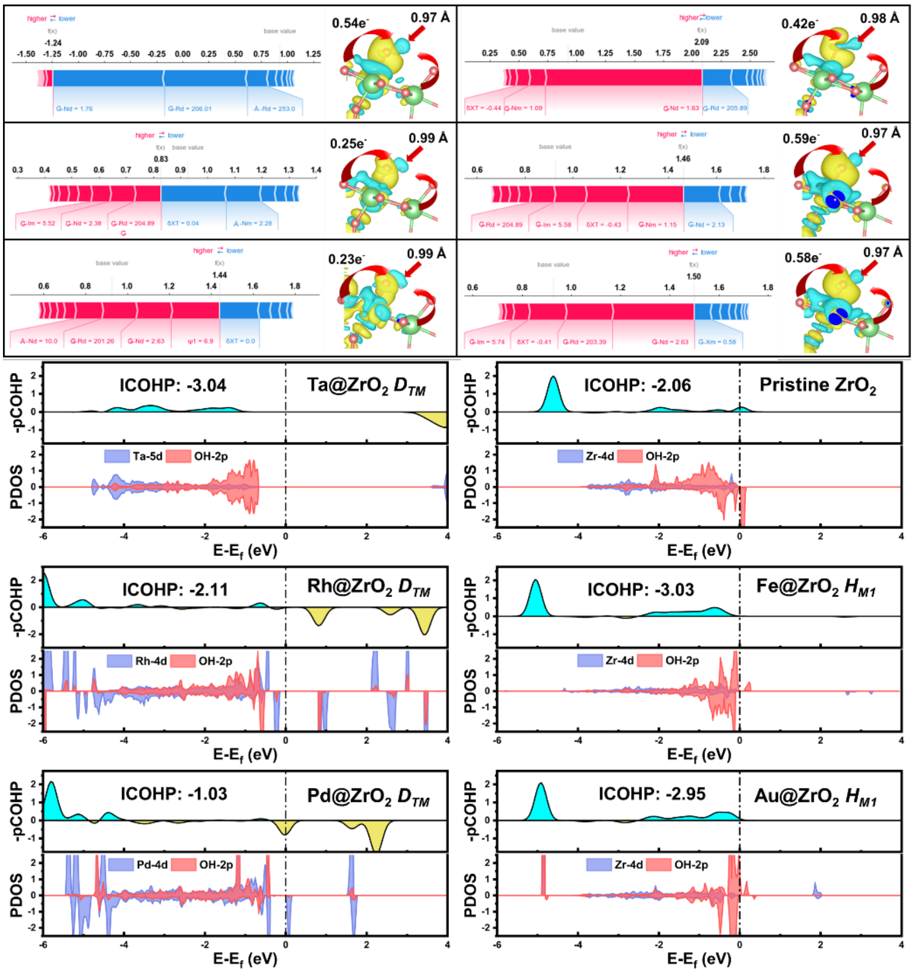

Figure S39. SHAP force plots and electronic structure analysis of D_{TM} and H_{M1} sites on TM@ZrO_2.

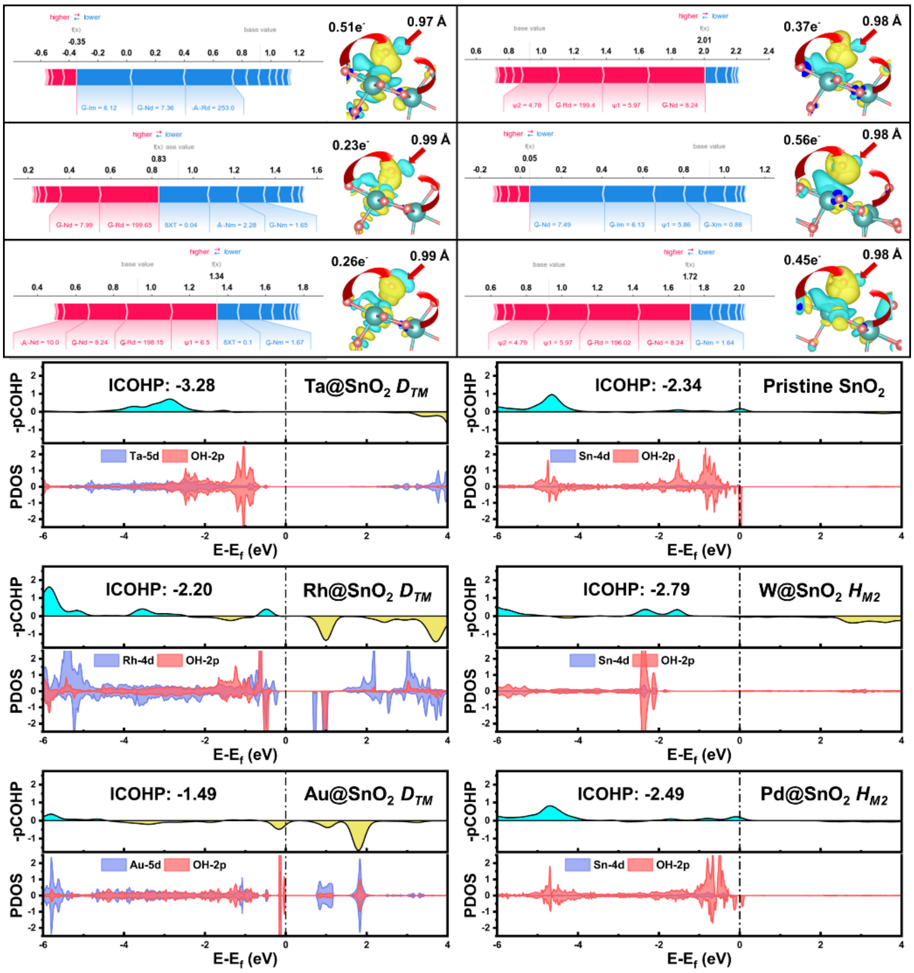

Figure S40. SHAP force plots and electronic structure analysis of D_{TM} and H_{M2} sites on TM@SnO_2.

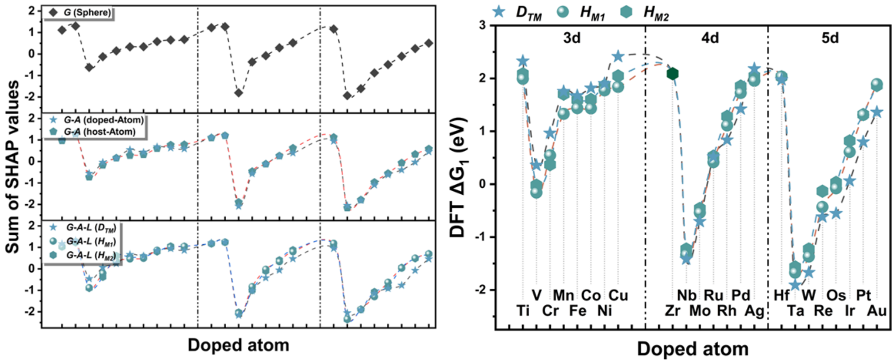

Figure S41. Global SHAP plot of TM@ZrO_2 and global results plot of DFT ΔG_1.

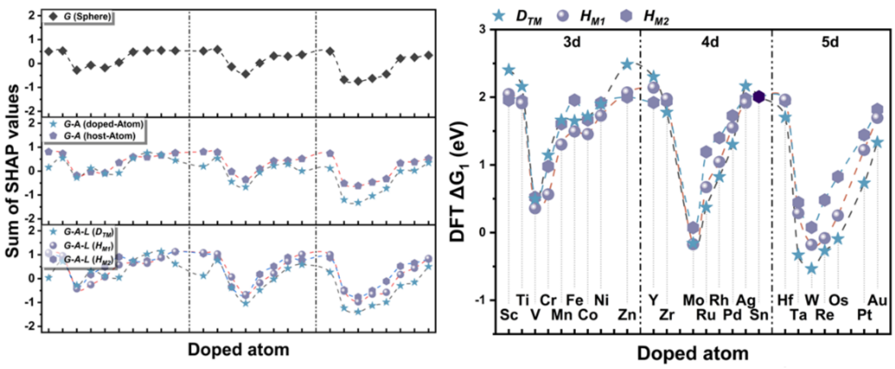

Figure S42. Global SHAP plot of TM@SnO_2 and global results plot of DFT ΔG_1.

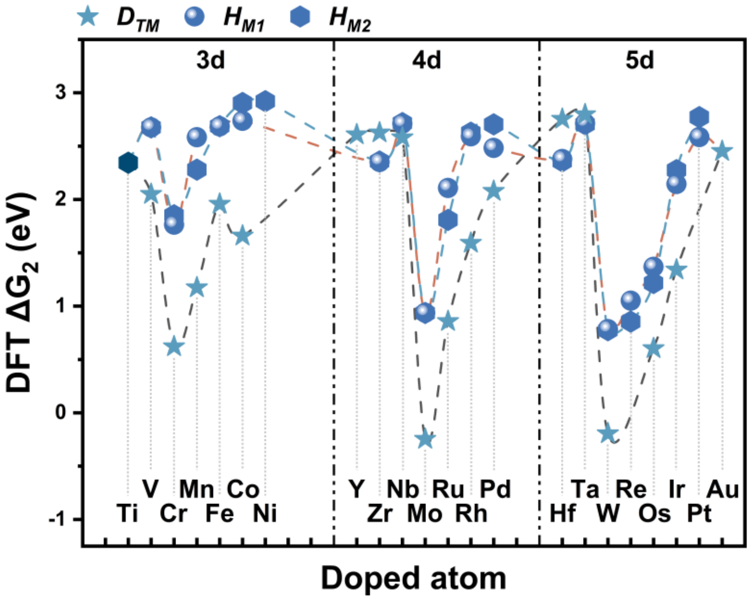

Figure S43. Global results plot of DFT ΔG_2 for $TM@TiO_2$.

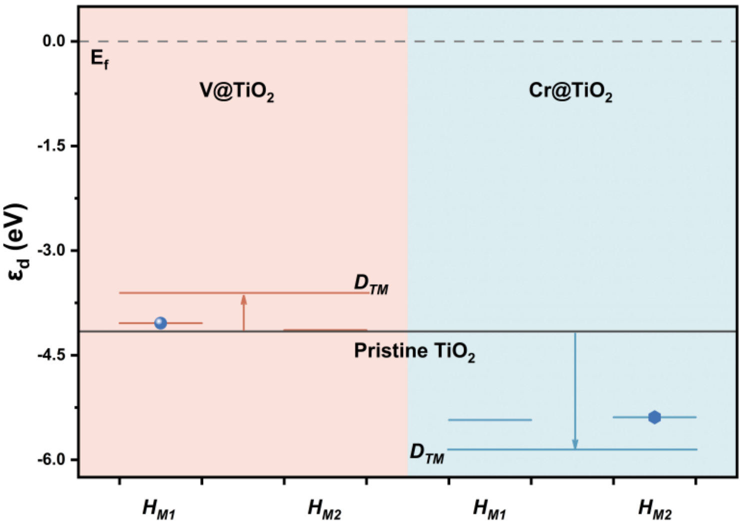

Figure S44. Modulation map of local *d*-band center positions. The red/blue regions denote the distributions of V@TiO_2 and Cr@TiO_2, respectively. The gray dashed line marks the Fermi level. The gray solid line indicates the ε_d of Ti atoms in pristine TiO_2. The red/blue long solid lines represent the ε_d of the D_{TM} sites in V@TiO_2 and Cr@TiO_2, respectively. The short solid lines correspond to the ε_d of the host sites (H_{M1}, H_{M2}). Sites marked with circles/hexagons are the optimal OH adsorption sites, associated with lower adsorption energies.

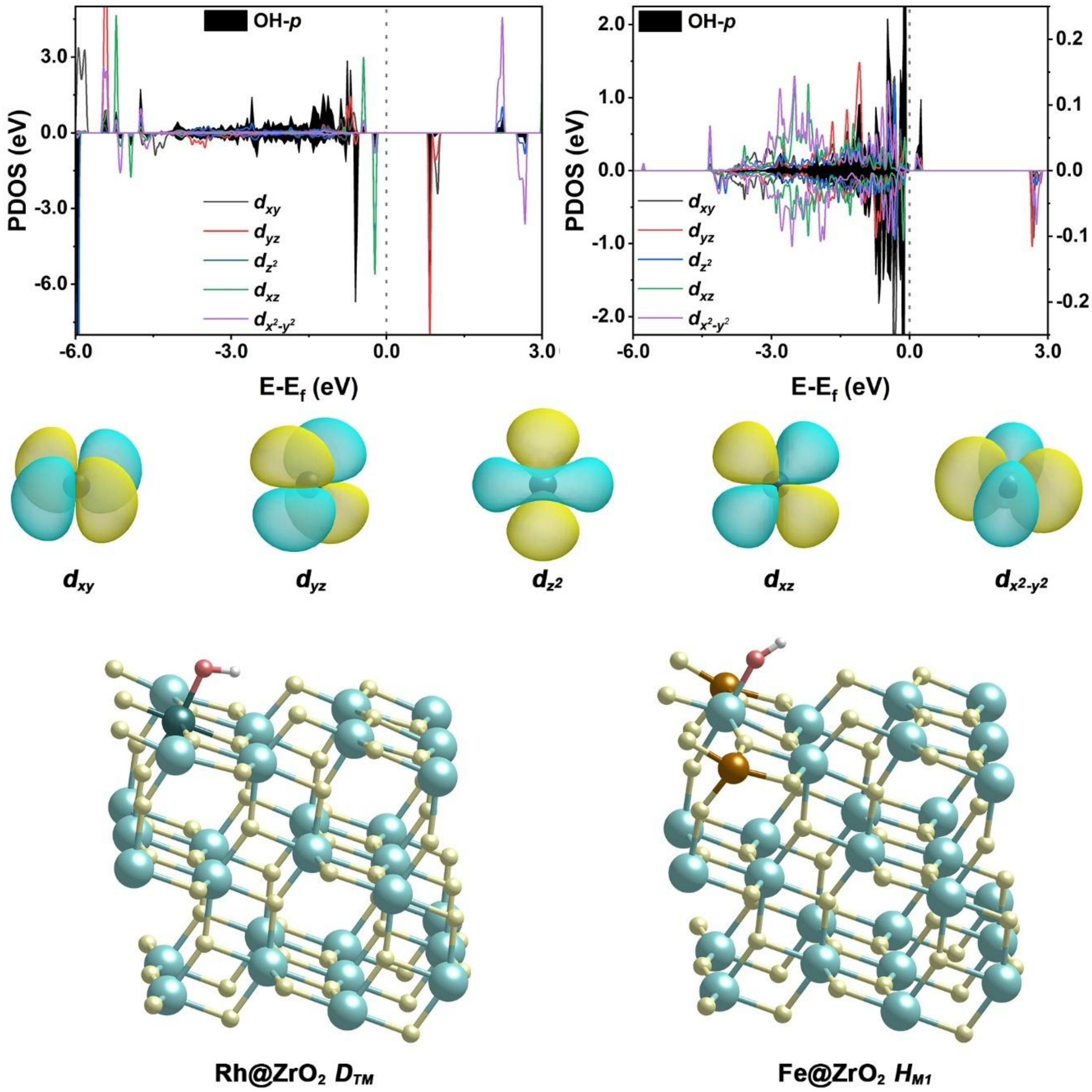

Figure S45. Analysis of electronic and geometric configurations of OH* at the Rh@ZrO_2 D_{TM} site and Fe@ZrO_2 H_{M1} site. From top to bottom: PDOS of the OH-*p* orbitals and M-*d* orbitals under OH adsorption; schematic of the five split *d*-orbitals of the M atom; geometric configuration of OH*.

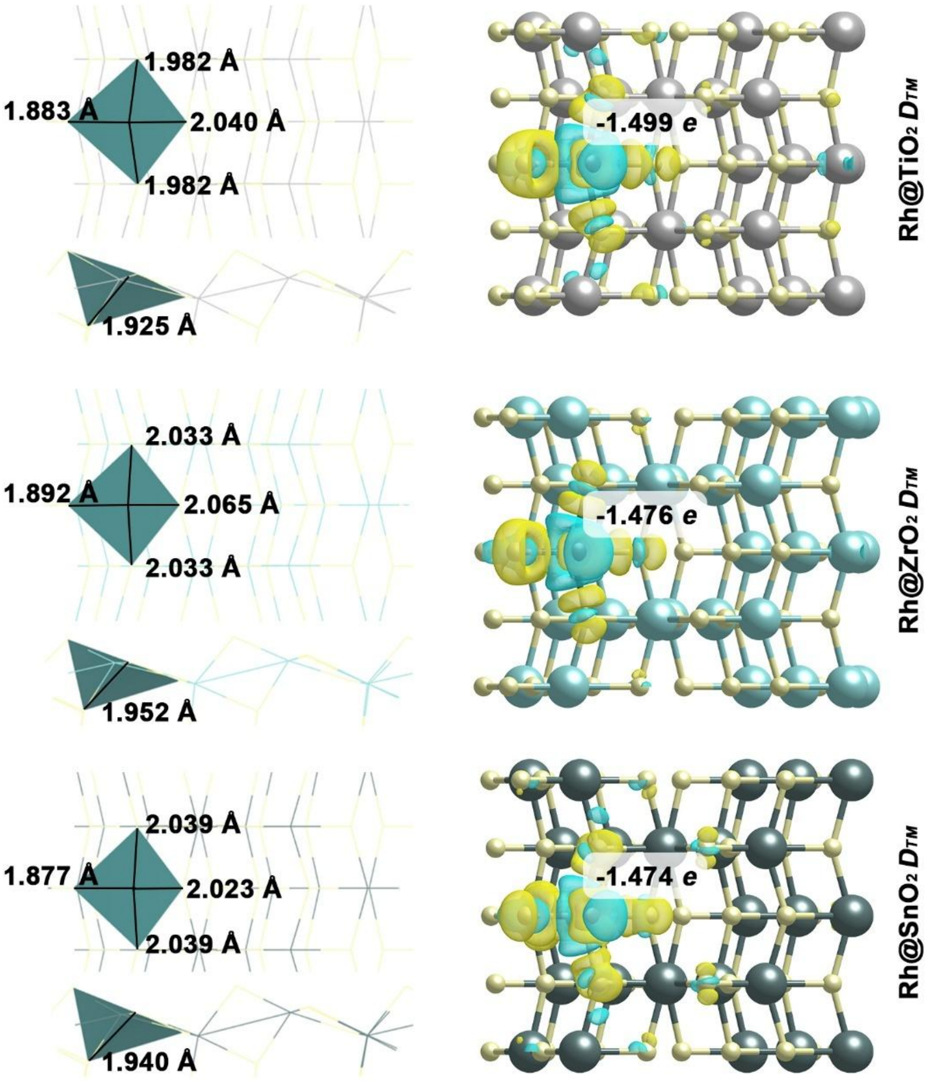

Figure S46. Geometric and electronic effects influencing the reactivity of the doping site. From top to bottom: Rh@MO_2 (M=Ti, Zr, Sn). Left panel: Pyramidal coordination fields of the Rh doping site on different metal oxides; Right panel: Charge distribution at the Rh sites ((±0.002 $e\cdot$bohr^{-3}, with yellow/cyan representing electron density accumulation and depletion, respectively)).

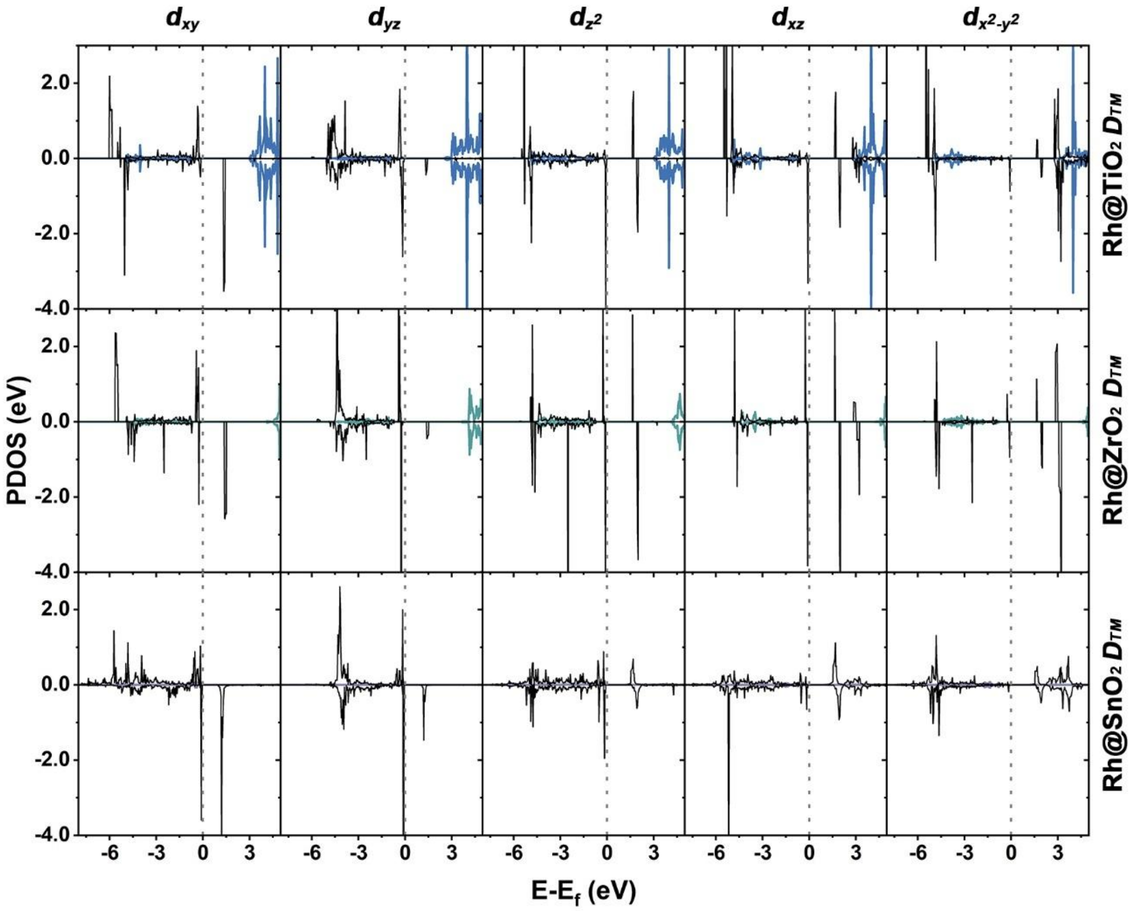

Figure S47. *d*-Orbital splitting and distribution at the Rh doping site under geometric and electronic effects. From top to bottom: Rh@MO_2 (M=Ti, Zr, Sn). Blue, green, and purple colors represent the *d*-orbital distribution of the corresponding M atom in pristine MO_2, respectively.

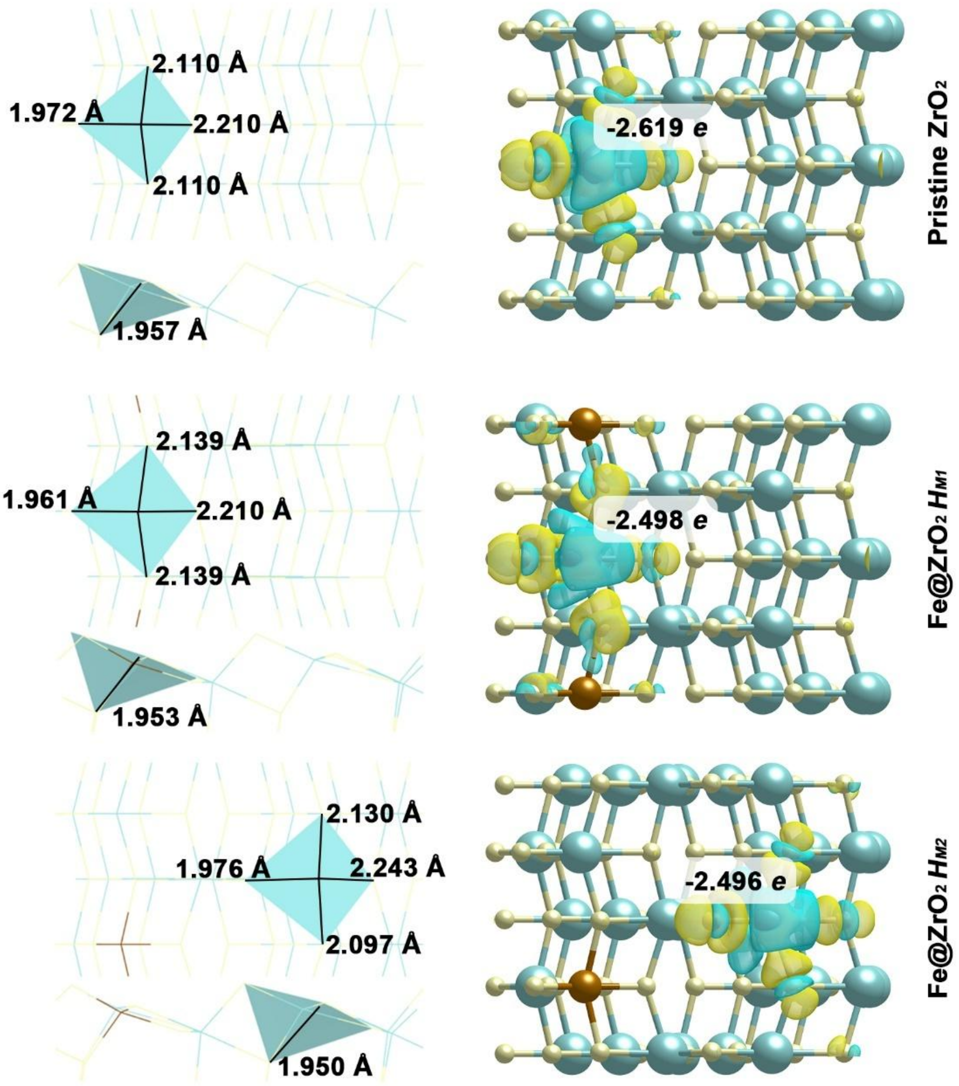

Figure S48. Geometric and electronic effects influencing the reactivity of the host site. From top to bottom: Pristine ZrO_2, Fe@ZrO_2 D_{M1} site, Fe@ZrO_2 D_{M2} site. Left panel: Pyramidal coordination field of the Zr host site on different metal oxides; Right panel: Charge distribution at the Zr site ((± 0.002 $e\cdot$bohr^{-3}, with yellow/cyan representing electron density accumulation and depletion, respectively)).

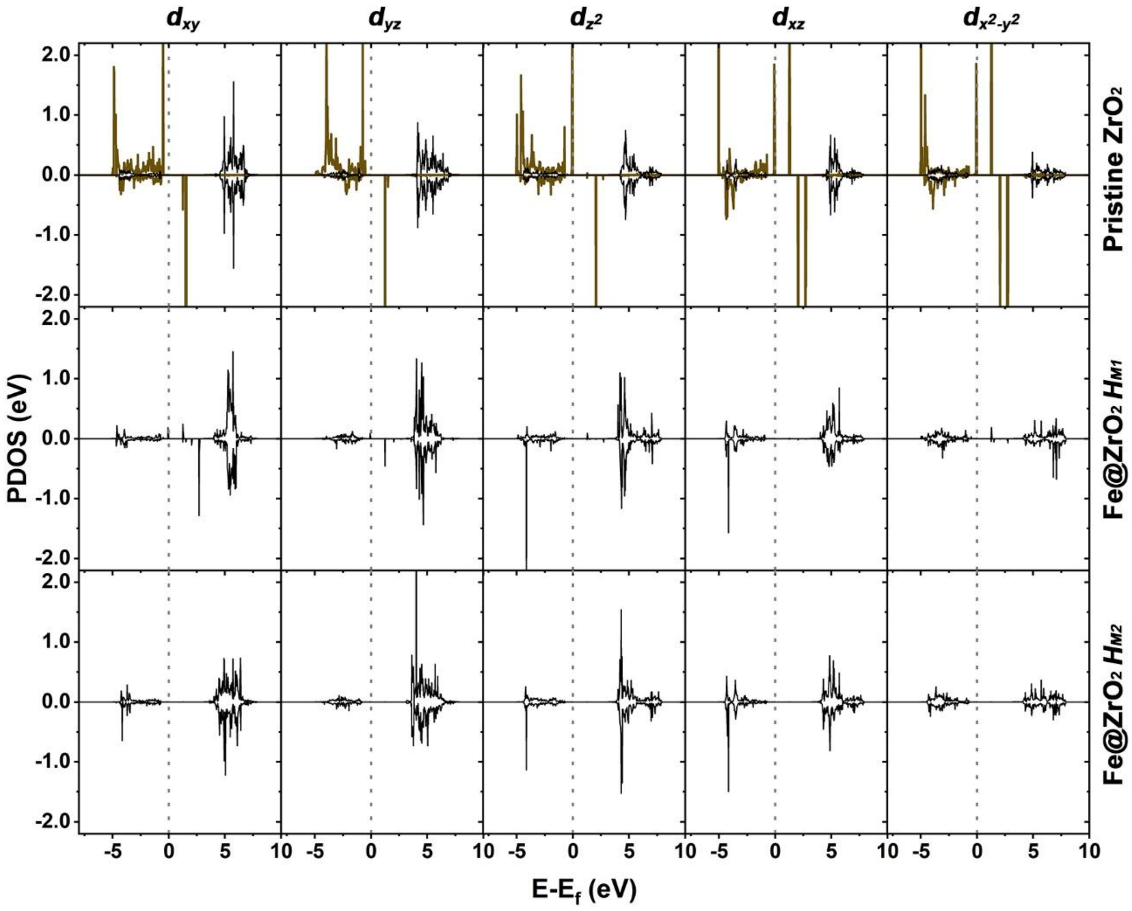

Figure S49. *d*-Orbital splitting and distribution at the Zr host site under geometric and electronic effects. From top to bottom: Pristine ZrO_2, Fe@ZrO_2 H_{M1} site, Fe@ZrO_2 H_{M2} site. Brown represents the *d*-orbital distribution at the Fe@ZrO_2 D_{TM} site.

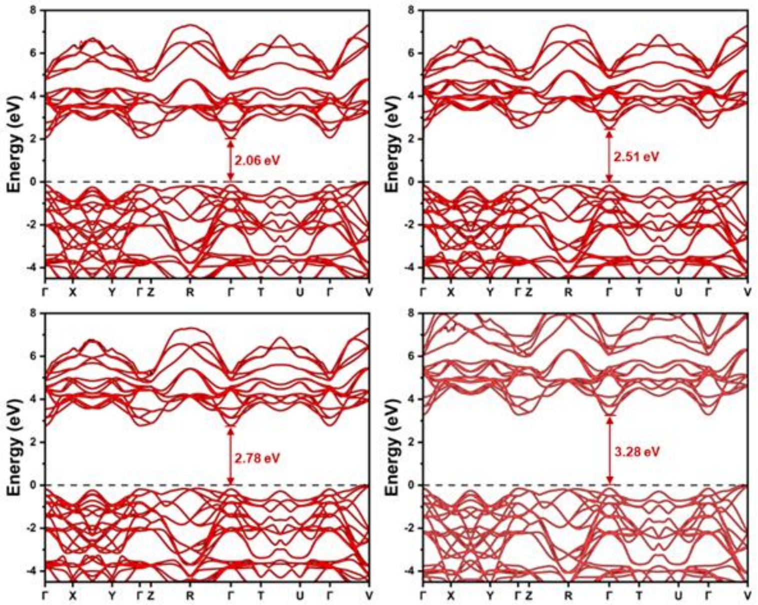

Figure S50. Energy band gaps of bulk TiO_2 calculated using different methods.

Supplementary Tables

Table S1. Relevant parameters for the geometric structure weight w_L of MO_2.

MO_2	d_{TM} (Å)	d_{M1} (Å)	d_{M2} (Å)	d_c (Å)
TiO_2	0	3.80	5.49	12.80
ZrO_2	0	4.03	6.05	13.97
SnO_2	0	4.04	5.90	13.71

Table S2. Relevant parameters for the geometric structure weight w_G of MO_2.

MO_2	d_s (Å)	d_{s2} (Å)
TiO_2	3.80	2.85
ZrO_2	4.03	3.18
SnO_2	4.04	3.12

Table S3. Comparison of lattice constants of MO_2 obtained from DFT calculations and values from the Materials Project database.

MO_2	DFT calculated values (Å)			Database values (Å)		
	a	b	c	a	b	c
TiO_2	3.80	3.80	9.57	3.78	3.78	9.62
ZrO_2	4.03	4.03	10.68	4.02	4.02	10.69
IrO_2	3.91	3.91	9.74	3.93	3.93	9.78
SnO_2	4.04	4.04	10.31	4.00	4.00	10.25

Table S4. Table of ***G***-class features for TM@TiO_2.

TM	Site	N_d	R_V (pm)	I_M (eV)	X_M	N_M (eV)
Sc	D_{TM}	1.81	239.25	6.57	1.47	0.09
	H_{M1}	1.81	239.25	6.57	1.47	0.09
	H_{M2}	1.81	239.25	6.57	1.47	0.09
Ti	D_{TM}	1.93	237.75	6.60	1.49	0.08
	H_{M1}	1.93	237.75	6.60	1.49	0.08
	H_{M2}	1.93	237.75	6.60	1.49	0.08
V	D_{TM}	2.06	237.25	6.59	1.50	0.13
	H_{M1}	2.06	237.25	6.59	1.50	0.13
	H_{M2}	2.06	237.25	6.59	1.50	0.13
Cr	D_{TM}	2.31	237.62	6.59	1.50	0.15
	H_{M1}	2.31	237.62	6.59	1.50	0.15
	H_{M2}	2.31	237.62	6.59	1.50	0.15
Mn	D_{TM}	2.31	237.62	6.67	1.49	0.07
	H_{M1}	2.31	237.62	6.67	1.49	0.07
	H_{M2}	2.31	237.62	6.67	1.49	0.07
Fe	D_{TM}	2.43	237.50	6.73	1.52	0.09
	H_{M1}	2.43	237.50	6.73	1.52	0.09
	H_{M2}	2.43	237.50	6.73	1.52	0.09
Co	D_{TM}	2.56	237.00	6.73	1.53	0.15
	H_{M1}	2.56	237.00	6.73	1.53	0.15
	H_{M2}	2.56	237.00	6.73	1.53	0.15
Ni	D_{TM}	2.68	237.00	6.70	1.53	0.21
	H_{M1}	2.68	237.00	6.70	1.53	0.21
	H_{M2}	2.68	237.00	6.70	1.53	0.21
Cu	D_{TM}	2.93	236.75	6.71	1.53	0.22
	H_{M1}	2.93	236.75	6.71	1.53	0.22
	H_{M2}	2.93	236.75	6.71	1.53	0.22
Zn	D_{TM}	2.93	236.87	6.92	1.50	0.07
	H_{M1}	2.93	236.87	6.92	1.50	0.07

	H_{M2}	2.93	236.87	6.92	1.50	0.07
Y	D_{TM}	1.81	241.37	6.52	1.45	0.11
	H_{M1}	1.81	241.37	6.52	1.45	0.11
	H_{M2}	1.81	241.37	6.52	1.45	0.11
Zr	D_{TM}	1.93	238.50	6.57	1.46	0.12
	H_{M1}	1.93	238.50	6.57	1.46	0.12
	H_{M2}	1.93	238.50	6.57	1.46	0.12
Nb	D_{TM}	2.18	239.00	6.59	1.49	0.18
	H_{M1}	2.18	239.00	6.59	1.49	0.18
	H_{M2}	2.18	239.00	6.59	1.49	0.18
Mo	D_{TM}	2.31	237.62	6.63	1.57	0.16
	H_{M1}	2.31	237.62	6.63	1.57	0.16
	H_{M2}	2.31	237.62	6.63	1.57	0.16
Ru	D_{TM}	2.56	237.75	6.67	1.57	0.20
	H_{M1}	2.56	237.75	6.67	1.57	0.20
	H_{M2}	2.56	237.75	6.67	1.57	0.20
Rh	D_{TM}	2.68	237.50	6.68	1.58	0.21
	H_{M1}	2.68	237.50	6.68	1.58	0.21
	H_{M2}	2.68	237.50	6.68	1.58	0.21
Pd	D_{TM}	2.93	233.87	6.79	1.57	0.14
	H_{M1}	2.93	233.87	6.79	1.57	0.14
	H_{M2}	2.93	233.87	6.79	1.57	0.14
Ag	D_{TM}	2.93	238.62	6.69	1.54	0.23
	H_{M1}	2.93	238.62	6.69	1.54	0.23
	H_{M2}	2.93	238.62	6.69	1.54	0.23
Hf	D_{TM}	1.93	239.87	6.60	1.46	0.07
	H_{M1}	1.93	239.87	6.60	1.46	0.07
	H_{M2}	1.93	239.87	6.60	1.46	0.07
Ta	D_{TM}	2.06	238.62	6.73	1.48	0.11
	H_{M1}	2.06	238.62	6.73	1.48	0.11

	H_{M2}	2.06	238.62	6.73	1.48	0.11
W	D_{TM}	2.18	239.12	6.74	1.51	0.17
	H_{M1}	2.18	239.12	6.74	1.51	0.17
	H_{M2}	2.18	239.12	6.74	1.51	0.17
Re	D_{TM}	2.31	238.12	6.73	1.54	0.09
	H_{M1}	2.31	238.12	6.73	1.54	0.09
	H_{M2}	2.31	238.12	6.73	1.54	0.09
Os	D_{TM}	2.43	238.00	6.83	1.57	0.20
	H_{M1}	2.43	238.00	6.83	1.57	0.20
	H_{M2}	2.43	238.00	6.83	1.57	0.20
Ir	D_{TM}	2.56	237.12	6.89	1.57	0.26
	H_{M1}	2.56	237.12	6.89	1.57	0.26
	H_{M2}	2.56	237.12	6.89	1.57	0.26
Pt	D_{TM}	2.81	235.62	6.87	1.57	0.33
	H_{M1}	2.81	235.62	6.87	1.57	0.33
	H_{M2}	2.81	235.62	6.87	1.57	0.33
Au	D_{TM}	2.93	236.00	6.90	1.60	0.36
	H_{M1}	2.93	236.00	6.90	1.60	0.36
	H_{M2}	2.93	236.00	6.90	1.60	0.36

Table S5. Table of ***G***-class features for TM@ZrO_2.

TM	Site	N_d	R_V (pm)	I_M (eV)	X_M	N_M (eV)
Sc	D_{TM}	1.76	238.59	6.25	1.26	0.37
	H_{M1}	1.76	238.59	6.25	1.26	0.37
	H_{M2}	1.76	238.59	6.25	1.26	0.37
Ti	D_{TM}	1.89	237.09	6.29	1.28	0.36
	H_{M1}	1.89	237.09	6.29	1.28	0.36
	H_{M2}	1.89	237.09	6.29	1.28	0.36
V	D_{TM}	2.01	236.59	6.28	1.29	0.41
	H_{M1}	2.01	236.59	6.28	1.29	0.41
	H_{M2}	2.01	236.59	6.28	1.29	0.41
Cr	D_{TM}	2.26	236.97	6.28	1.30	0.43
	H_{M1}	2.26	236.97	6.28	1.30	0.43
	H_{M2}	2.26	236.97	6.28	1.30	0.43
Mn	D_{TM}	2.26	236.97	6.36	1.28	0.35
	H_{M1}	2.26	236.97	6.36	1.28	0.35
	H_{M2}	2.26	236.97	6.36	1.28	0.35
Fe	D_{TM}	2.39	236.84	6.42	1.32	0.37
	H_{M1}	2.39	236.84	6.42	1.32	0.37
	H_{M2}	2.39	236.84	6.42	1.32	0.37
Co	D_{TM}	2.51	236.34	6.42	1.32	0.43
	H_{M1}	2.51	236.34	6.42	1.32	0.43
	H_{M2}	2.51	236.34	6.42	1.32	0.43
Ni	D_{TM}	2.64	236.34	6.39	1.33	0.49
	H_{M1}	2.64	236.34	6.39	1.33	0.49
	H_{M2}	2.64	236.34	6.39	1.33	0.49
Cu	D_{TM}	2.89	236.09	6.40	1.33	0.50
	H_{M1}	2.89	236.09	6.40	1.33	0.50
	H_{M2}	2.89	236.09	6.40	1.33	0.50
Zn	D_{TM}	2.89	236.22	6.61	1.30	0.35
	H_{M1}	2.89	236.22	6.61	1.30	0.35

	H_{M2}	2.89	236.22	6.61	1.30	0.35
Y	D_{TM}	1.76	240.72	6.21	1.24	0.39
	H_{M1}	1.76	240.72	6.21	1.24	0.39
	H_{M2}	1.76	240.72	6.21	1.24	0.39
Zr	D_{TM}	1.89	237.84	6.26	1.26	0.40
	H_{M1}	1.89	237.84	6.26	1.26	0.40
	H_{M2}	1.89	237.84	6.26	1.26	0.40
Nb	D_{TM}	2.14	238.34	6.28	1.29	0.46
	H_{M1}	2.14	238.34	6.28	1.29	0.46
	H_{M2}	2.14	238.34	6.28	1.29	0.46
Mo	D_{TM}	2.26	236.97	6.32	1.36	0.44
	H_{M1}	2.26	236.97	6.32	1.36	0.44
	H_{M2}	2.26	236.97	6.32	1.36	0.44
Ru	D_{TM}	2.51	237.09	6.35	1.36	0.48
	H_{M1}	2.51	237.09	6.35	1.36	0.48
	H_{M2}	2.51	237.09	6.35	1.36	0.48
Rh	D_{TM}	2.64	236.84	6.36	1.37	0.49
	H_{M1}	2.64	236.84	6.36	1.37	0.49
	H_{M2}	2.64	236.84	6.36	1.37	0.49
Pd	D_{TM}	2.89	233.22	6.47	1.36	0.42
	H_{M1}	2.89	233.22	6.47	1.36	0.42
	H_{M2}	2.89	233.22	6.47	1.36	0.42
Ag	D_{TM}	2.89	237.97	6.38	1.33	0.51
	H_{M1}	2.89	237.97	6.38	1.33	0.51
	H_{M2}	2.89	237.97	6.38	1.33	0.51
Hf	D_{TM}	1.89	239.22	6.29	1.25	0.35
	H_{M1}	1.89	239.22	6.29	1.25	0.35
	H_{M2}	1.89	239.22	6.29	1.25	0.35
Ta	D_{TM}	2.01	237.97	6.42	1.28	0.39
	H_{M1}	2.01	237.97	6.42	1.28	0.39

	H_{M2}	2.01	237.97	6.42	1.28	0.39
W	D_{TM}	2.14	238.47	6.43	1.30	0.46
	H_{M1}	2.14	238.47	6.43	1.30	0.46
	H_{M2}	2.14	238.47	6.43	1.30	0.46
Re	D_{TM}	2.26	237.47	6.42	1.33	0.37
	H_{M1}	2.26	237.47	6.42	1.33	0.37
	H_{M2}	2.26	237.47	6.42	1.33	0.37
Os	D_{TM}	2.39	237.34	6.52	1.36	0.49
	H_{M1}	2.39	237.34	6.52	1.36	0.49
	H_{M2}	2.39	237.34	6.52	1.36	0.49
Ir	D_{TM}	2.51	236.47	6.57	1.36	0.55
	H_{M1}	2.51	236.47	6.57	1.36	0.55
	H_{M2}	2.51	236.47	6.57	1.36	0.55
Pt	D_{TM}	2.76	234.97	6.56	1.36	0.61
	H_{M1}	2.76	234.97	6.56	1.36	0.61
	H_{M2}	2.76	234.97	6.56	1.36	0.61
Au	D_{TM}	2.89	235.34	6.59	1.39	0.64
	H_{M1}	2.89	235.34	6.59	1.39	0.64
	H_{M2}	2.89	235.34	6.59	1.39	0.64

Table S6. Table of ***G***-class features for TM@SnO_2.

TM	Site	N_d	R_V (pm)	I_M (eV)	X_M	N_M (eV)
Sc	D_{TM}	8.41	232.76	6.90	1.79	0.94
	H_{M1}	8.41	232.76	6.90	1.79	0.94
	H_{M2}	8.41	232.76	6.90	1.79	0.94
Ti	D_{TM}	8.54	231.26	6.93	1.82	0.93
	H_{M1}	8.54	231.26	6.93	1.82	0.93
	H_{M2}	8.54	231.26	6.93	1.82	0.93
V	D_{TM}	8.66	230.76	6.92	1.83	0.99
	H_{M1}	8.66	230.76	6.92	1.83	0.99
	H_{M2}	8.66	230.76	6.92	1.83	0.99
Cr	D_{TM}	8.91	231.13	6.93	1.83	1.00
	H_{M1}	8.91	231.13	6.93	1.83	1.00
	H_{M2}	8.91	231.13	6.93	1.83	1.00
Mn	D_{TM}	8.91	231.13	7.01	1.82	0.92
	H_{M1}	8.91	231.13	7.01	1.82	0.92
	H_{M2}	8.91	231.13	7.01	1.82	0.92
Fe	D_{TM}	9.04	231.01	7.07	1.85	0.94
	H_{M1}	9.04	231.01	7.07	1.85	0.94
	H_{M2}	9.04	231.01	7.07	1.85	0.94
Co	D_{TM}	9.16	230.51	7.07	1.86	1.00
	H_{M1}	9.16	230.51	7.07	1.86	1.00
	H_{M2}	9.16	230.51	7.07	1.86	1.00
Ni	D_{TM}	9.29	230.51	7.04	1.86	1.06
	H_{M1}	9.29	230.51	7.04	1.86	1.06
	H_{M2}	9.29	230.51	7.04	1.86	1.06
Cu	D_{TM}	9.54	230.26	7.05	1.86	1.07
	H_{M1}	9.54	230.26	7.05	1.86	1.07
	H_{M2}	9.54	230.26	7.05	1.86	1.07
Zn	D_{TM}	9.54	230.38	7.26	1.83	0.92
	H_{M1}	9.54	230.38	7.26	1.83	0.92

	H_{M2}	9.54	230.38	7.26	1.83	0.92
Y	D_{TM}	8.41	234.88	6.86	1.78	0.96
	H_{M1}	8.41	234.88	6.86	1.78	0.96
	H_{M2}	8.41	234.88	6.86	1.78	0.96
Zr	D_{TM}	8.54	232.01	6.91	1.79	0.97
	H_{M1}	8.54	232.01	6.91	1.79	0.97
	H_{M2}	8.54	232.01	6.91	1.79	0.97
Nb	D_{TM}	8.79	232.51	6.93	1.82	1.03
	H_{M1}	8.79	232.51	6.93	1.82	1.03
	H_{M2}	8.79	232.51	6.93	1.82	1.03
Mo	D_{TM}	8.91	231.13	6.97	1.89	1.01
	H_{M1}	8.91	231.13	6.97	1.89	1.01
	H_{M2}	8.91	231.13	6.97	1.89	1.01
Ru	D_{TM}	9.16	231.26	7.00	1.90	1.05
	H_{M1}	9.16	231.26	7.00	1.90	1.05
	H_{M2}	9.16	231.26	7.00	1.90	1.05
Rh	D_{TM}	9.29	231.01	7.01	1.91	1.06
	H_{M1}	9.29	231.01	7.01	1.91	1.06
	H_{M2}	9.29	231.01	7.01	1.91	1.06
Pd	D_{TM}	9.54	227.38	7.12	1.90	0.99
	H_{M1}	9.54	227.38	7.12	1.90	0.99
	H_{M2}	9.54	227.38	7.12	1.90	0.99
Ag	D_{TM}	9.54	232.13	7.03	1.87	1.08
	H_{M1}	9.54	232.13	7.03	1.87	1.08
	H_{M2}	9.54	232.13	7.03	1.87	1.08
Hf	D_{TM}	8.54	233.38	6.93	1.79	0.92
	H_{M1}	8.54	233.38	6.93	1.79	0.92
	H_{M2}	8.54	233.38	6.93	1.79	0.92
Ta	D_{TM}	8.66	232.13	7.07	1.81	0.96
	H_{M1}	8.66	232.13	7.07	1.81	0.96

	H_{M2}	8.66	232.13	7.07	1.81	0.96
W	D_{TM}	8.79	232.63	7.08	1.84	1.03
	H_{M1}	8.79	232.63	7.08	1.84	1.03
	H_{M2}	8.79	232.63	7.08	1.84	1.03
Re	D_{TM}	8.91	231.63	7.07	1.87	0.94
	H_{M1}	8.91	231.63	7.07	1.87	0.94
	H_{M2}	8.91	231.63	7.07	1.87	0.94
Os	D_{TM}	9.04	231.51	7.17	1.90	1.06
	H_{M1}	9.04	231.51	7.17	1.90	1.06
	H_{M2}	9.04	231.51	7.17	1.90	1.06
Ir	D_{TM}	9.16	230.63	7.22	1.90	1.12
	H_{M1}	9.16	230.63	7.22	1.90	1.12
	H_{M2}	9.16	230.63	7.22	1.90	1.12
Pt	D_{TM}	9.41	229.13	7.21	1.90	1.19
	H_{M1}	9.41	229.13	7.21	1.90	1.19
	H_{M2}	9.41	229.13	7.21	1.90	1.19
Au	D_{TM}	9.54	229.51	7.23	1.92	1.21
	H_{M1}	9.54	229.51	7.23	1.92	1.21
	H_{M2}	9.54	229.51	7.23	1.92	1.21

Table S7. Table of ***A***-class features for TM@TiO_2.

TM	Site	N_d	R_V (pm)	I_M (eV)	X_M	N_M (eV)
Sc	D_{TM}	1	258	6.56	1.36	0.19
	H_{M1}	2	246	6.83	1.54	0.08
	H_{M2}	2	246	6.83	1.54	0.08
Ti	D_{TM}	2	246	6.83	1.54	0.08
	H_{M1}	2	246	6.83	1.54	0.08
	H_{M2}	2	246	6.83	1.54	0.08
V	D_{TM}	3	242	6.75	1.63	0.52
	H_{M1}	2	246	6.83	1.54	0.08
	H_{M2}	2	246	6.83	1.54	0.08
Cr	D_{TM}	5	245	6.77	1.66	0.67
	H_{M1}	2	246	6.83	1.54	0.08
	H_{M2}	2	246	6.83	1.54	0.08
Mn	D_{TM}	5	245	7.43	1.55	0.00
	H_{M1}	2	246	6.83	1.54	0.08
	H_{M2}	2	246	6.83	1.54	0.08
Fe	D_{TM}	6	244	7.90	1.83	0.15
	H_{M1}	2	246	6.83	1.54	0.08
	H_{M2}	2	246	6.83	1.54	0.08
Co	D_{TM}	7	240	7.88	1.88	0.66
	H_{M1}	2	246	6.83	1.54	0.08
	H_{M2}	2	246	6.83	1.54	0.08
Ni	D_{TM}	8	240	7.64	1.91	1.16
	H_{M1}	2	246	6.83	1.54	0.08
	H_{M2}	2	246	6.83	1.54	0.08
Cu	D_{TM}	10	238	7.73	1.90	1.24
	H_{M1}	2	246	6.83	1.54	0.08
	H_{M2}	2	246	6.83	1.54	0.08
Zn	D_{TM}	10	239	9.39	1.65	0.00
	H_{M1}	2	246	6.83	1.54	0.08

	H_{M2}	2	246	6.83	1.54	0.08
Y	D_{TM}	1	275	6.22	1.22	0.31
	H_{M1}	2	246	6.83	1.54	0.08
	H_{M2}	2	246	6.83	1.54	0.08
Zr	D_{TM}	2	252	6.63	1.33	0.43
	H_{M1}	2	246	6.83	1.54	0.08
	H_{M2}	2	246	6.83	1.54	0.08
Nb	D_{TM}	4	256	6.76	1.59	0.89
	H_{M1}	2	246	6.83	1.54	0.08
	H_{M2}	2	246	6.83	1.54	0.08
Mo	D_{TM}	5	245	7.09	2.16	0.75
	H_{M1}	2	246	6.83	1.54	0.08
	H_{M2}	2	246	6.83	1.54	0.08
Ru	D_{TM}	7	246	7.36	2.20	1.05
	H_{M1}	2	246	6.83	1.54	0.08
	H_{M2}	2	246	6.83	1.54	0.08
Rh	D_{TM}	8	244	7.46	2.28	1.14
	H_{M1}	2	246	6.83	1.54	0.08
	H_{M2}	2	246	6.83	1.54	0.08
Pd	D_{TM}	10	215	8.34	2.20	0.56
	H_{M1}	2	246	6.83	1.54	0.08
	H_{M2}	2	246	6.83	1.54	0.08
Ag	D_{TM}	10	253	7.58	1.93	1.30
	H_{M1}	2	246	6.83	1.54	0.08
	H_{M2}	2	246	6.83	1.54	0.08
Hf	D_{TM}	2	263	6.83	1.32	0.00
	H_{M1}	2	246	6.83	1.54	0.08
	H_{M2}	2	246	6.83	1.54	0.08
Ta	D_{TM}	3	253	7.89	1.51	0.32
	H_{M1}	2	246	6.83	1.54	0.08

	H_{M2}	2	246	6.83	1.54	0.08
W	D_{TM}	4	257	7.98	1.70	0.86
	H_{M1}	2	246	6.83	1.54	0.08
	H_{M2}	2	246	6.83	1.54	0.08
Re	D_{TM}	5	249	7.88	1.93	0.15
	H_{M1}	2	246	6.83	1.54	0.08
	H_{M2}	2	246	6.83	1.54	0.08
Os	D_{TM}	6	248	8.71	2.18	1.10
	H_{M1}	2	246	6.83	1.54	0.08
	H_{M2}	2	246	6.83	1.54	0.08
Ir	D_{TM}	7	241	9.12	2.20	1.57
	H_{M1}	2	246	6.83	1.54	0.08
	H_{M2}	2	246	6.83	1.54	0.08
Pt	D_{TM}	9	229	9.02	2.20	2.13
	H_{M1}	2	246	6.83	1.54	0.08
	H_{M2}	2	246	6.83	1.54	0.08
Au	D_{TM}	10	232	9.23	2.40	2.31
	H_{M1}	2	246	6.83	1.54	0.08
	H_{M2}	2	246	6.83	1.54	0.08

Table S8. Table of ***A***-class features for TM@ZrO_2.

TM	Site	N_d	R_V (pm)	I_M (eV)	X_M	N_M (eV)
Sc	D_{TM}	1	258	6.56	1.36	0.19
	H_{M1}	2	252	6.63	1.33	0.43
	H_{M2}	2	252	6.63	1.33	0.43
Ti	D_{TM}	2	246	6.83	1.54	0.08
	H_{M1}	2	252	6.63	1.33	0.43
	H_{M2}	2	252	6.63	1.33	0.43
V	D_{TM}	3	242	6.75	1.63	0.52
	H_{M1}	2	252	6.63	1.33	0.43
	H_{M2}	2	252	6.63	1.33	0.43
Cr	D_{TM}	5	245	6.77	1.66	0.67
	H_{M1}	2	252	6.63	1.33	0.43
	H_{M2}	2	252	6.63	1.33	0.43
Mn	D_{TM}	5	245	7.43	1.55	0.00
	H_{M1}	2	252	6.63	1.33	0.43
	H_{M2}	2	252	6.63	1.33	0.43
Fe	D_{TM}	6	244	7.90	1.83	0.15
	H_{M1}	2	252	6.63	1.33	0.43
	H_{M2}	2	252	6.63	1.33	0.43
Co	D_{TM}	7	240	7.88	1.88	0.66
	H_{M1}	2	252	6.63	1.33	0.43
	H_{M2}	2	252	6.63	1.33	0.43
Ni	D_{TM}	8	240	7.64	1.91	1.16
	H_{M1}	2	252	6.63	1.33	0.43
	H_{M2}	2	252	6.63	1.33	0.43
Cu	D_{TM}	10	238	7.73	1.90	1.24
	H_{M1}	2	252	6.63	1.33	0.43
	H_{M2}	2	252	6.63	1.33	0.43
Zn	D_{TM}	10	239	9.39	1.65	0.00
	H_{M1}	2	252	6.63	1.33	0.43

	H_{M2}	2	252	6.63	1.33	0.43
Y	D_{TM}	1	275	6.22	1.22	0.31
	H_{M1}	2	252	6.63	1.33	0.43
	H_{M2}	2	252	6.63	1.33	0.43
Zr	D_{TM}	2	252	6.63	1.33	0.43
	H_{M1}	2	252	6.63	1.33	0.43
	H_{M2}	2	252	6.63	1.33	0.43
Nb	D_{TM}	4	256	6.76	1.59	0.89
	H_{M1}	2	252	6.63	1.33	0.43
	H_{M2}	2	252	6.63	1.33	0.43
Mo	D_{TM}	5	245	7.09	2.16	0.75
	H_{M1}	2	252	6.63	1.33	0.43
	H_{M2}	2	252	6.63	1.33	0.43
Ru	D_{TM}	7	246	7.36	2.20	1.05
	H_{M1}	2	252	6.63	1.33	0.43
	H_{M2}	2	252	6.63	1.33	0.43
Rh	D_{TM}	8	244	7.46	2.28	1.14
	H_{M1}	2	252	6.63	1.33	0.43
	H_{M2}	2	252	6.63	1.33	0.43
Pd	D_{TM}	10	215	8.34	2.20	0.56
	H_{M1}	2	252	6.63	1.33	0.43
	H_{M2}	2	252	6.63	1.33	0.43
Ag	D_{TM}	10	253	7.58	1.93	1.30
	H_{M1}	2	252	6.63	1.33	0.43
	H_{M2}	2	252	6.63	1.33	0.43
Hf	D_{TM}	2	263	6.83	1.32	0.00
	H_{M1}	2	252	6.63	1.33	0.43
	H_{M2}	2	252	6.63	1.33	0.43
Ta	D_{TM}	3	253	7.89	1.51	0.32
	H_{M1}	2	252	6.63	1.33	0.43

	H_{M2}	2	252	6.63	1.33	0.43
W	D_{TM}	4	257	7.98	1.70	0.86
	H_{M1}	2	252	6.63	1.33	0.43
	H_{M2}	2	252	6.63	1.33	0.43
Re	D_{TM}	5	249	7.88	1.93	0.15
	H_{M1}	2	252	6.63	1.33	0.43
	H_{M2}	2	252	6.63	1.33	0.43
Os	D_{TM}	6	248	8.71	2.18	1.10
	H_{M1}	2	252	6.63	1.33	0.43
	H_{M2}	2	252	6.63	1.33	0.43
Ir	D_{TM}	7	241	9.12	2.20	1.57
	H_{M1}	2	252	6.63	1.33	0.43
	H_{M2}	2	252	6.63	1.33	0.43
Pt	D_{TM}	9	229	9.02	2.20	2.13
	H_{M1}	2	252	6.63	1.33	0.43
	H_{M2}	2	252	6.63	1.33	0.43
Au	D_{TM}	10	232	9.23	2.40	2.31
	H_{M1}	2	252	6.63	1.33	0.43
	H_{M2}	2	252	6.63	1.33	0.43

Table S8. Table of ***A***-class features for TM@SnO_2.

TM	Site	N_d	R_V (pm)	I_M (eV)	X_M	N_M (eV)
Sc	D_{TM}	1	258	6.56	1.36	0.19
	H_{M1}	10	242	7.34	1.96	1.11
	H_{M2}	10	242	7.34	1.96	1.11
Ti	D_{TM}	2	246	6.83	1.54	0.08
	H_{M1}	10	242	7.34	1.96	1.11
	H_{M2}	10	242	7.34	1.96	1.11
V	D_{TM}	3	242	6.75	1.63	0.52
	H_{M1}	10	242	7.34	1.96	1.11
	H_{M2}	10	242	7.34	1.96	1.11
Cr	D_{TM}	5	245	6.77	1.66	0.67
	H_{M1}	10	242	7.34	1.96	1.11
	H_{M2}	10	242	7.34	1.96	1.11
Mn	D_{TM}	5	245	7.43	1.55	0.00
	H_{M1}	10	242	7.34	1.96	1.11
	H_{M2}	10	242	7.34	1.96	1.11
Fe	D_{TM}	6	244	7.90	1.83	0.15
	H_{M1}	10	242	7.34	1.96	1.11
	H_{M2}	10	242	7.34	1.96	1.11
Co	D_{TM}	7	240	7.88	1.88	0.66
	H_{M1}	10	242	7.34	1.96	1.11
	H_{M2}	10	242	7.34	1.96	1.11
Ni	D_{TM}	8	240	7.64	1.91	1.16
	H_{M1}	10	242	7.34	1.96	1.11
	H_{M2}	10	242	7.34	1.96	1.11
Cu	D_{TM}	10	238	7.73	1.90	1.24
	H_{M1}	10	242	7.34	1.96	1.11
	H_{M2}	10	242	7.34	1.96	1.11
Zn	D_{TM}	10	239	9.39	1.65	0.00
	H_{M1}	10	242	7.34	1.96	1.11

	H_{M2}	10	242	7.34	1.96	1.11
Y	D_{TM}	1	275	6.22	1.22	0.31
	H_{M1}	10	242	7.34	1.96	1.11
	H_{M2}	10	242	7.34	1.96	1.11
Zr	D_{TM}	2	252	6.63	1.33	0.43
	H_{M1}	10	242	7.34	1.96	1.11
	H_{M2}	10	242	7.34	1.96	1.11
Nb	D_{TM}	4	256	6.76	1.59	0.89
	H_{M1}	10	242	7.34	1.96	1.11
	H_{M2}	10	242	7.34	1.96	1.11
Mo	D_{TM}	5	245	7.09	2.16	0.75
	H_{M1}	10	242	7.34	1.96	1.11
	H_{M2}	10	242	7.34	1.96	1.11
Ru	D_{TM}	7	246	7.36	2.20	1.05
	H_{M1}	10	242	7.34	1.96	1.11
	H_{M2}	10	242	7.34	1.96	1.11
Rh	D_{TM}	8	244	7.46	2.28	1.14
	H_{M1}	10	242	7.34	1.96	1.11
	H_{M2}	10	242	7.34	1.96	1.11
Pd	D_{TM}	10	215	8.34	2.20	0.56
	H_{M1}	10	242	7.34	1.96	1.11
	H_{M2}	10	242	7.34	1.96	1.11
Ag	D_{TM}	10	253	7.58	1.93	1.30
	H_{M1}	10	242	7.34	1.96	1.11
	H_{M2}	10	242	7.34	1.96	1.11
Hf	D_{TM}	2	263	6.83	1.32	0.00
	H_{M1}	10	242	7.34	1.96	1.11
	H_{M2}	10	242	7.34	1.96	1.11
Ta	D_{TM}	3	253	7.89	1.51	0.32
	H_{M1}	10	242	7.34	1.96	1.11

	H_{M2}	10	242	7.34	1.96	1.11
W	D_{TM}	4	257	7.98	1.70	0.86
	H_{M1}	10	242	7.34	1.96	1.11
	H_{M2}	10	242	7.34	1.96	1.11
Re	D_{TM}	5	249	7.88	1.93	0.15
	H_{M1}	10	242	7.34	1.96	1.11
	H_{M2}	10	242	7.34	1.96	1.11
Os	D_{TM}	6	248	8.71	2.18	1.10
	H_{M1}	10	242	7.34	1.96	1.11
	H_{M2}	10	242	7.34	1.96	1.11
Ir	D_{TM}	7	241	9.12	2.20	1.57
	H_{M1}	10	242	7.34	1.96	1.11
	H_{M2}	10	242	7.34	1.96	1.11
Pt	D_{TM}	9	229	9.02	2.20	2.13
	H_{M1}	10	242	7.34	1.96	1.11
	H_{M2}	10	242	7.34	1.96	1.11
Au	D_{TM}	10	232	9.23	2.40	2.31
	H_{M1}	10	242	7.34	1.96	1.11
	H_{M2}	10	242	7.34	1.96	1.11

Table S9. Table of ***L***-class features for TM@TiO_2.

TM	Site	ψ_1	ψ_2	δ_{XT}	δ_{IE}
Sc	D_{TM}	0.59	0.52	-0.42	-7.06
	H_{M1}	1.08	0.87	-0.35	-6.84
	H_{M2}	1.10	0.88	-0.34	-6.84
Ti	D_{TM}	1.19	0.96	-0.33	-6.79
	H_{M1}	1.19	0.96	-0.33	-6.79
	H_{M2}	1.19	0.96	-0.33	-6.79
V	D_{TM}	1.81	1.37	-0.29	-6.87
	H_{M1}	1.31	1.03	-0.32	-6.81
	H_{M2}	1.29	1.02	-0.32	-6.81
Cr	D_{TM}	3.02	2.24	-0.27	-6.85
	H_{M1}	1.53	1.20	-0.32	-6.80
	H_{M2}	1.49	1.17	-0.32	-6.80
Mn	D_{TM}	2.99	2.38	-0.33	-6.19
	H_{M1}	1.53	1.22	-0.33	-6.68
	H_{M2}	1.49	1.19	-0.33	-6.69
Fe	D_{TM}	3.71	2.43	-0.19	-5.72
	H_{M1}	1.66	1.24	-0.30	-6.59
	H_{M2}	1.60	1.20	-0.31	-6.62
Co	D_{TM}	4.36	2.75	-0.16	-5.74
	H_{M1}	1.78	1.30	-0.30	-6.60
	H_{M2}	1.71	1.25	-0.30	-6.62
Ni	D_{TM}	5.01	3.08	-0.15	-5.98
	H_{M1}	1.90	1.36	-0.30	-6.64
	H_{M2}	1.81	1.31	-0.30	-6.66
Cu	D_{TM}	6.25	3.88	-0.15	-5.89
	H_{M1}	2.13	1.51	-0.30	-6.62
	H_{M2}	2.01	1.44	-0.30	-6.65
Zn	D_{TM}	6.04	4.51	-0.28	-4.23
	H_{M1}	2.09	1.62	-0.32	-6.32

	H_{M2}	1.98	1.54	-0.32	-6.37
Y	D_{TM}	0.58	0.56	-0.49	-7.40
	H_{M1}	1.08	0.88	-0.36	-6.90
	H_{M2}	1.09	0.89	-0.36	-6.89
Zr	D_{TM}	1.17	1.06	-0.44	-6.99
	H_{M1}	1.19	0.97	-0.35	-6.83
	H_{M2}	1.19	0.97	-0.35	-6.82
Nb	D_{TM}	2.40	1.86	-0.31	-6.86
	H_{M1}	1.42	1.13	-0.33	-6.80
	H_{M2}	1.39	1.10	-0.33	-6.80
Mo	D_{TM}	3.29	1.61	-0.02	-6.53
	H_{M1}	1.58	1.09	-0.27	-6.74
	H_{M2}	1.53	1.07	-0.28	-6.75
Ru	D_{TM}	4.66	2.18	0.00	-6.26
	H_{M1}	1.83	1.20	-0.27	-6.69
	H_{M2}	1.75	1.17	-0.28	-6.71
Rh	D_{TM}	5.44	2.34	0.04	-6.16
	H_{M1}	1.98	1.23	-0.26	-6.67
	H_{M2}	1.88	1.19	-0.27	-6.69
Pd	D_{TM}	6.65	3.12	0.00	-5.28
	H_{M1}	2.20	1.37	-0.27	-6.51
	H_{M2}	2.08	1.32	-0.28	-6.55
Ag	D_{TM}	6.28	3.80	-0.14	-6.04
	H_{M1}	2.14	1.49	-0.29	-6.65
	H_{M2}	2.02	1.43	-0.30	-6.67
Hf	D_{TM}	1.17	1.07	-0.44	-6.79
	H_{M1}	1.19	0.97	-0.35	-6.79
	H_{M2}	1.19	0.97	-0.35	-6.79
Ta	D_{TM}	1.78	1.46	-0.35	-5.73
	H_{M1}	1.30	1.05	-0.33	-6.59

	H_{M2}	1.29	1.04	-0.33	-6.62
W	D_{TM}	2.43	1.75	-0.25	-5.64
	H_{M1}	1.42	1.11	-0.32	-6.58
	H_{M2}	1.39	1.09	-0.32	-6.60
Re	D_{TM}	3.14	1.90	-0.14	-5.74
	H_{M1}	1.55	1.14	-0.29	-6.60
	H_{M2}	1.51	1.12	-0.30	-6.62
Os	D_{TM}	3.97	1.90	-0.01	-4.91
	H_{M1}	1.71	1.14	-0.27	-6.44
	H_{M2}	1.64	1.12	-0.28	-6.49
Ir	D_{TM}	4.66	2.18	0.00	-4.50
	H_{M1}	1.83	1.20	-0.27	-6.37
	H_{M2}	1.75	1.17	-0.28	-6.42
Pt	D_{TM}	5.99	2.81	0.00	-4.60
	H_{M1}	2.08	1.31	-0.27	-6.38
	H_{M2}	1.97	1.27	-0.28	-6.44
Au	D_{TM}	7.10	2.62	0.10	-4.40
	H_{M1}	2.29	1.28	-0.25	-6.35
	H_{M2}	2.15	1.24	-0.26	-6.40

Table S10. Table of ***L***-class features for TM@ZrO_2.

TM	Site	ψ_1	ψ_2	δ_{XT}	δ_{IE}
Sc	D_{TM}	0.60	0.47	-0.42	-7.06
	H_{M1}	1.08	0.87	-0.43	-7.00
	H_{M2}	1.10	0.88	-0.43	-7.00
Ti	D_{TM}	1.22	0.86	-0.33	-6.79
	H_{M1}	1.20	0.94	-0.42	-6.95
	H_{M2}	1.20	0.94	-0.42	-6.95
V	D_{TM}	1.85	1.23	-0.29	-6.87
	H_{M1}	1.32	1.01	-0.41	-6.97
	H_{M2}	1.30	1.01	-0.41	-6.97
Cr	D_{TM}	3.09	2.02	-0.27	-6.85
	H_{M1}	1.55	1.16	-0.40	-6.96
	H_{M2}	1.50	1.13	-0.41	-6.96
Mn	D_{TM}	3.05	2.14	-0.33	-6.19
	H_{M1}	1.54	1.18	-0.41	-6.84
	H_{M2}	1.49	1.15	-0.42	-6.86
Fe	D_{TM}	3.81	2.19	-0.19	-5.72
	H_{M1}	1.68	1.20	-0.39	-6.75
	H_{M2}	1.62	1.16	-0.39	-6.78
Co	D_{TM}	4.48	2.48	-0.16	-5.74
	H_{M1}	1.81	1.25	-0.38	-6.75
	H_{M2}	1.72	1.21	-0.39	-6.78
Ni	D_{TM}	5.15	2.77	-0.15	-5.98
	H_{M1}	1.93	1.31	-0.38	-6.80
	H_{M2}	1.83	1.26	-0.39	-6.82
Cu	D_{TM}	6.43	3.49	-0.15	-5.89
	H_{M1}	2.17	1.44	-0.38	-6.78
	H_{M2}	2.04	1.38	-0.39	-6.81
Zn	D_{TM}	6.18	4.06	-0.28	-4.23
	H_{M1}	2.13	1.54	-0.41	-6.47

	H_{M2}	2.00	1.46	-0.41	-6.54
Y	D_{TM}	0.59	0.50	-0.49	-7.40
	H_{M1}	1.08	0.87	-0.45	-7.06
	H_{M2}	1.10	0.88	-0.44	-7.05
Zr	D_{TM}	1.19	0.96	-0.44	-6.99
	H_{M1}	1.19	0.96	-0.44	-6.99
	H_{M2}	1.19	0.96	-0.44	-6.99
Nb	D_{TM}	2.45	1.68	-0.31	-6.86
	H_{M1}	1.43	1.10	-0.41	-6.96
	H_{M2}	1.40	1.08	-0.41	-6.97
Mo	D_{TM}	3.41	1.45	-0.02	-6.53
	H_{M1}	1.61	1.06	-0.36	-6.90
	H_{M2}	1.55	1.05	-0.37	-6.91
Ru	D_{TM}	4.83	1.97	0.00	-6.26
	H_{M1}	1.87	1.16	-0.35	-6.85
	H_{M2}	1.78	1.14	-0.36	-6.87
Rh	D_{TM}	5.67	2.10	0.04	-6.16
	H_{M1}	2.03	1.19	-0.35	-6.83
	H_{M2}	1.92	1.16	-0.36	-6.85
Pd	D_{TM}	6.90	2.81	0.00	-5.28
	H_{M1}	2.26	1.32	-0.35	-6.67
	H_{M2}	2.12	1.27	-0.36	-6.71
Ag	D_{TM}	6.46	3.42	-0.14	-6.04
	H_{M1}	2.18	1.43	-0.38	-6.81
	H_{M2}	2.05	1.37	-0.39	-6.83
Hf	D_{TM}	1.19	0.96	-0.44	-6.79
	H_{M1}	1.19	0.96	-0.44	-6.95
	H_{M2}	1.19	0.96	-0.44	-6.96
Ta	D_{TM}	1.82	1.31	-0.35	-5.73
	H_{M1}	1.31	1.03	-0.42	-6.75

	H_{M2}	1.29	1.02	-0.42	-6.78
W	D_{TM}	2.49	1.58	-0.25	-5.64
	H_{M1}	1.43	1.08	-0.40	-6.73
	H_{M2}	1.40	1.06	-0.41	-6.77
Re	D_{TM}	3.23	1.71	-0.14	-5.74
	H_{M1}	1.57	1.11	-0.38	-6.75
	H_{M2}	1.52	1.09	-0.39	-6.78
Os	D_{TM}	4.12	1.71	-0.01	-4.91
	H_{M1}	1.74	1.11	-0.36	-6.60
	H_{M2}	1.66	1.09	-0.37	-6.65
Ir	D_{TM}	4.83	1.97	0.00	-4.50
	H_{M1}	1.87	1.16	-0.35	-6.52
	H_{M2}	1.78	1.14	-0.36	-6.58
Pt	D_{TM}	6.21	2.53	0.00	-4.60
	H_{M1}	2.13	1.27	-0.35	-6.54
	H_{M2}	2.00	1.23	-0.36	-6.60
Au	D_{TM}	7.45	2.36	0.10	-4.40
	H_{M1}	2.36	1.24	-0.33	-6.50
	H_{M2}	2.20	1.20	-0.35	-6.57

Table S11. Table of ***L***-class features for TM@SnO_2.

TM	Site	ψ_1	ψ_2	δ_{XT}	δ_{IE}
Sc	D_{TM}	0.57	0.67	-0.42	-7.06
	H_{M1}	4.98	3.95	-0.18	-6.43
	H_{M2}	5.10	4.04	-0.17	-6.41
Ti	D_{TM}	1.14	1.23	-0.33	-6.79
	H_{M1}	5.08	4.07	-0.16	-6.38
	H_{M2}	5.19	4.16	-0.15	-6.36
V	D_{TM}	1.73	1.75	-0.29	-6.87
	H_{M1}	5.19	4.18	-0.15	-6.39
	H_{M2}	5.29	4.25	-0.15	-6.38
Cr	D_{TM}	2.89	2.88	-0.27	-6.85
	H_{M1}	5.40	4.39	-0.15	-6.39
	H_{M2}	5.47	4.44	-0.14	-6.37
Mn	D_{TM}	2.86	3.05	-0.33	-6.19
	H_{M1}	5.40	4.41	-0.16	-6.26
	H_{M2}	5.47	4.45	-0.15	-6.26
Fe	D_{TM}	3.52	3.12	-0.19	-5.72
	H_{M1}	5.52	4.46	-0.13	-6.18
	H_{M2}	5.57	4.50	-0.13	-6.19
Co	D_{TM}	4.14	3.53	-0.16	-5.74
	H_{M1}	5.63	4.54	-0.13	-6.18
	H_{M2}	5.67	4.57	-0.13	-6.19
Ni	D_{TM}	4.74	3.96	-0.15	-5.98
	H_{M1}	5.74	4.62	-0.12	-6.22
	H_{M2}	5.77	4.64	-0.12	-6.23
Cu	D_{TM}	5.92	4.98	-0.15	-5.89
	H_{M1}	5.96	4.81	-0.13	-6.21
	H_{M2}	5.96	4.81	-0.12	-6.22
Zn	D_{TM}	5.77	5.78	-0.28	-4.23
	H_{M1}	5.94	4.93	-0.15	-5.90

	H_{M2}	5.94	4.91	-0.15	-5.95
Y	D_{TM}	0.56	0.72	-0.49	-7.40
	H_{M1}	4.98	3.94	-0.19	-6.49
	H_{M2}	5.11	4.04	-0.18	-6.46
Zr	D_{TM}	1.13	1.36	-0.44	-6.99
	H_{M1}	5.08	4.08	-0.18	-6.41
	H_{M2}	5.20	4.15	-0.17	-6.39
Nb	D_{TM}	2.30	2.39	-0.31	-6.86
	H_{M1}	5.29	4.30	-0.15	-6.39
	H_{M2}	5.38	4.35	-0.15	-6.37
Mo	D_{TM}	3.08	2.07	-0.02	-6.53
	H_{M1}	5.42	4.30	-0.10	-6.33
	H_{M2}	5.49	4.36	-0.10	-6.32
Ru	D_{TM}	4.34	2.80	0.00	-6.26
	H_{M1}	5.66	4.44	-0.10	-6.28
	H_{M2}	5.69	4.49	-0.10	-6.28
Rh	D_{TM}	5.04	3.00	0.04	-6.16
	H_{M1}	5.79	4.49	-0.09	-6.26
	H_{M2}	5.81	4.53	-0.09	-6.26
Pd	D_{TM}	6.20	4.01	0.00	-5.28
	H_{M1}	6.00	4.67	-0.10	-6.09
	H_{M2}	6.00	4.68	-0.10	-6.12
Ag	D_{TM}	5.94	4.88	-0.14	-6.04
	H_{M1}	5.96	4.80	-0.12	-6.24
	H_{M2}	5.96	4.79	-0.12	-6.24
Hf	D_{TM}	1.13	1.37	-0.44	-6.79
	H_{M1}	5.08	4.08	-0.18	-6.38
	H_{M2}	5.20	4.15	-0.17	-6.36
Ta	D_{TM}	1.71	1.87	-0.35	-5.73
	H_{M1}	5.19	4.19	-0.16	-6.18

	H_{M2}	5.29	4.26	-0.16	-6.19
W	D_{TM}	2.32	2.25	-0.25	-5.64
	H_{M1}	5.29	4.28	-0.14	-6.16
	H_{M2}	5.38	4.34	-0.14	-6.18
Re	D_{TM}	2.97	2.44	-0.14	-5.74
	H_{M1}	5.41	4.34	-0.12	-6.18
	H_{M2}	5.48	4.40	-0.12	-6.19
Os	D_{TM}	3.70	2.44	-0.01	-4.91
	H_{M1}	5.54	4.37	-0.10	-6.02
	H_{M2}	5.59	4.43	-0.10	-6.06
Ir	D_{TM}	4.34	2.80	0.00	-4.50
	H_{M1}	5.66	4.44	-0.10	-5.95
	H_{M2}	5.69	4.49	-0.10	-5.99
Pt	D_{TM}	5.58	3.61	0.00	-4.60
	H_{M1}	5.89	4.59	-0.10	-5.97
	H_{M2}	5.90	4.62	-0.10	-6.01
Au	D_{TM}	6.50	3.36	0.10	-4.40
	H_{M1}	6.05	4.57	-0.08	-5.93
	H_{M2}	6.04	4.60	-0.08	-5.97
